# Vi-specific serological correlates of protection for typhoid fever

**DOI:** 10.1084/jem.20201116

**Published:** 2020-11-12

**Authors:** Celina Jin, Jennifer Hill, Bronwyn M. Gunn, Wen-Han Yu, Lindsay C. Dahora, Elizabeth Jones, Mari Johnson, Malick M. Gibani, Rachel L. Spreng, S. Munir Alam, Anna Nebykova, Helene B. Juel, S. Moses Dennison, Kelly E. Seaton, Jonathan K. Fallon, Georgia D. Tomaras, Galit Alter, Andrew J. Pollard

**Affiliations:** 1 Oxford Vaccine Group, Department of Pediatrics, University of Oxford, Oxford, UK; 2 National Institute for Health Research Oxford Biomedical Research Centre, Oxford, UK; 3 Ragon Institute of Massachusetts General Hospital, Massachusetts Institute of Technology, and Harvard University, Cambridge, MA; 4 Departments of Immunology, Surgery, and Molecular Genetics and Microbiology, Duke Human Vaccine Institute, Duke University, Durham, NC

## Abstract

Typhoid Vi vaccines have been shown to be efficacious in children living in endemic regions; however, a widely accepted correlate of protection remains to be established. We applied a systems serology approach to identify Vi-specific serological correlates of protection using samples obtained from participants enrolled in an experimental controlled human infection study. Participants were vaccinated with Vi-tetanus toxoid conjugate (Vi-TT) or unconjugated Vi-polysaccharide (Vi-PS) vaccines and were subsequently challenged with *Salmonella* Typhi bacteria. Multivariate analyses identified distinct protective signatures for Vi-TT and Vi-PS vaccines in addition to shared features that predicted protection across both groups. Vi IgA quantity and avidity correlated with protection from *S.* Typhi infection, whereas higher fold increases in Vi IgG responses were associated with reduced disease severity. Targeted antibody-mediated functional responses, particularly neutrophil phagocytosis, were also identified as important components of the protective signature. These humoral markers could be used to evaluate and develop efficacious Vi-conjugate vaccines and assist with accelerating vaccine availability to typhoid-endemic regions.

## Teaser

Distinct protective humoral signatures are induced by Vi-conjugate and Vi-polysaccharide vaccines. Shared humoral features across both vaccines suggest that protection against typhoid fever is mediated through a combination of qualitative features of the polyclonal humoral response (avidity and innate immune cell functional responses) in addition to antibody quantity.

## Introduction

Typhoid fever is a systemic febrile illness that affects 9–13 million individuals globally each year (Stanaway et al., 2019). Incidence rates are highest among children living in low- and lower-middle–income countries who lack access to safe water. Antimicrobial resistance among *Salmonella* Typhi (*S.* Typhi) strains is increasing. The current fluoroquinolone-ceftriaxone extensively drug-resistant typhoid fever outbreak occurring in Pakistan has highlighted the need for urgent intervention (Klemm et al., 2018). Fortunately, several advancements toward disease control have been made in recent years. In 2018, the World Health Organization recommended the programmatic use of typhoid conjugate vaccines (TCVs) to protect children living in high-burden endemic regions (World Health Organization, 2018), and the Global Vaccine Alliance pledged $85 million to assist with the rollout of TCVs in Global Vaccine Alliance–eligible countries (The Lancet, 2018). In December 2019, the first efficacy data evaluating the only World Health Organization–prequalified TCV (Typbar-TCV) in children aged 9 months to 15 years from an endemic region were published. Vaccine efficacy was estimated at 81% (95% confidence interval [CI] 58.8–91.8) after 15-month follow-up (Shakya et al., 2019).

There is no doubt that demand for TCVs will increase in the coming years. Several TCVs in development are immunogenic and safe but lack efficacy data (Bhutta et al., 2014; Capeding et al., 2020). Vaccine-induced correlates of protection could be used to screen TCV candidates to identify efficacious vaccines, removing the need for large-scale efficacy studies and thereby accelerating the process of vaccine development and licensure. Correlates of protection have already been used to license other types of conjugate vaccines, such as the capsular group C meningococcal conjugate vaccine (Nguipdop Djomo et al., 2013) and newer pneumococcal conjugate vaccines (Jódar et al., 2003). Currently, widely accepted correlates of protection for typhoid fever do not exist. Protective thresholds of Vi IgG titers, extrapolated from Vi-polysaccharide (Vi-PS) and Vi-recombinant *Pseudomonas aeruginosa* exotoxin A carrier conjugate vaccine (Vi-rEPA; TCV with recombinant *Pseudomonas aeruginosa* exotoxin A carrier protein) efficacy trials, have been difficult to replicate owing to differences in antibody detection methods. Furthermore, limited availability of sera from the original studies has prevented bridging studies from being successfully undertaken (Szu et al., 2014).

It can be difficult to identify serological correlates of protection from large vaccine efficacy trials, as not all vaccinees are guaranteed to be exposed to the pathogen, and sera are usually not available from all participants. However, direct correlations can be made in experimental controlled human infection models (CHIMs), in which volunteers are deliberately infected with *S.* Typhi. In 2017, we published the first efficacy data for a Vi-tetanus toxoid conjugate (Vi-TT) vaccine, which was evaluated using a *S.* Typhi CHIM. Participants were randomized to vaccination with Vi-TT, unconjugated Vi-PS, or control (meningococcal ACWY conjugate) vaccines and were orally challenged with live *S.* Typhi bacteria in a bicarbonate solution ∼28 days later. Typhoid fever was diagnosed in individuals with *S*. Typhi bacteremia or clinical features of disease (persistent fever ≥38°C). We observed attack rates of 77% (24/31) in the control group, 35% (13/37) in the Vi-TT group, and 37% (13/35) in the Vi-PS group, resulting in efficacy estimates of 54.6% (95% CI 26.8–71.8) for Vi-TT and 52.0% (95% CI 23.3–70.0) for Vi-PS (Jin et al., 2017).

Logistic regression modeling of Vi IgG titers from the CHIM efficacy study found that higher Vi IgG titers reduced the probability of typhoid fever diagnosis; however, an absolute threshold of protection could not be established. More recently, we have observed that higher Vi IgA titers induced by Vi vaccination were also associated with protection; however, once again, a protective threshold was not identified (Dahora et al., 2019). The median Vi IgA concentration in protected Vi-PS vaccinees was 504 µg/ml; however, the median concentration in diagnosed Vi-TT participants was similar, at 595 µg/ml. Protected Vi-TT vaccinees had a median Vi IgA concentration of 2,118 µg/ml (Dahora et al., 2019). These preliminary findings suggest that other subpopulations of antibodies, within the polyclonal Vi antibody response, may be involved in mediating protection against *S*. Typhi infection and that Vi-TT and Vi-PS induce protective responses through different humoral mechanisms.

Here, we employed a systems serology approach (Chung and Alter, 2017), using samples obtained from Vi-TT and Vi-PS vaccinees from the *S*. Typhi CHIM efficacy study described above, to further investigate potential correlates of protection following Vi vaccination.

## Results

### Vi-TT and Vi-PS are both immunogenic vaccines

**Figure 1.**
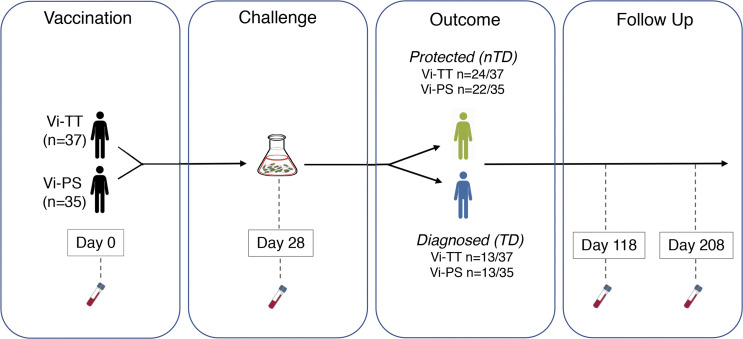
**Schematic of *S. *Typhi CHIM study design. **Participants were randomized, vaccinated with Vi-TT (*n* = 37) or Vi-PS (*n* = 35), orally challenged with live *S. *Typhi bacteria on day 28. Participants were monitored over a 14-day challenge period. Individuals with *S. *Typhi bacteremia or persistent fever (≥38°C for ≥12 h) were diagnosed with typhoid fever (TD; Vi-TT, *n* = 13/37; Vi-PS, *n* = 13/35). Participants who did not meet the diagnostic criteria for typhoid fever were classified as nTD (Vi-TT, *n* = 24/37; Vi-PS, *n* = 22/35). Vi-specific humoral responses were evaluated on day 0 (before vaccination) and days 28, 118, and 208.

Vi-specific humoral and cellular responses were evaluated using a panel of 35 assays (Table 1) in participants randomized to Vi-TT (*n* = 37) or Vi-PS (*n* = 35) vaccination. Quantitation of total Vi IgG titers in control participants demonstrated absent or low responses in most participants (Jin et al., 2017). As such, control participants were not included in these analyses. Vaccinated participants were orally challenged with *S*. Typhi bacteria on day 28 and intensively monitored over a 14-day period for evidence of typhoid infection. Typhoid-diagnosed (TD) participants were defined as individuals with *S.* Typhi detected from blood culture or those with persistent fever (≥38°C for ≥12 h). Participants who did not meet the predefined diagnostic criteria were presumed to be protected from infection (not typhoid diagnosed [nTD]; Fig. 1). Samples were collected at baseline (before vaccination), day 28 (immediately before *S.* Typhi challenge), and 118 and 208 days after vaccination (Fig. 1).

**Table 1.** Assays used to evaluate Vi-specific humoral responses

Assay	Measured Vi-specific humoral response	Vi-PS antigen (native or biotinylated)
**Antibody quantification**		
ELISA	IgG, IgG1, IgG2, IgG3, IgA, IgM	Native
BAMA	IgG1, IgG2, IgA, IgA1, IgA2	Native
	IgG1, IgG2, IgG3, IgA	Biotinylated
**Antibody avidity**		
BAMA-AI	IgG1 AI, IgA, AI IgA1 AI, IgA2 AI	Native
	IgG1 AI, IgG2 AI, IgG3 AI, IgA AI	Biotinylated
**Functional properties**		
ADCD	Complement deposition	Biotinylated
ADCP	Cellular phagocytosis (monocyte)	Biotinylated
ADNP	Neutrophil phagocytosis	Biotinylated
ADNKDA	NK cell MIP-1β release, IFNγ release, CD107a expression	Biotinylated
ADNOB	Neutrophil oxidative burst	Biotinylated
**FcR binding**		
Luminex	FcαR binding, FcγR2A binding, FcγR2B binding, FcγR3A binding, FcγR3B binding	Biotinylated

35 Vi-specific humoral responses were evaluated using different assay techniques on day 0 (before vaccination) and days 28, 118, and 208. Univariate analyses (comparing responses between time points within vaccine groups, across vaccine groups, and between diagnosed and protected participants) were performed for each individual antibody feature listed. Multivariate analyses included 34 of 35 of the antibody features listed; Vi IgG3 AI was not included in the multivariate analyses, as ≥25% of values were missing. Assays were performed using native Vi-PS or biotinylated Vi-PS.

**Figure 2.**
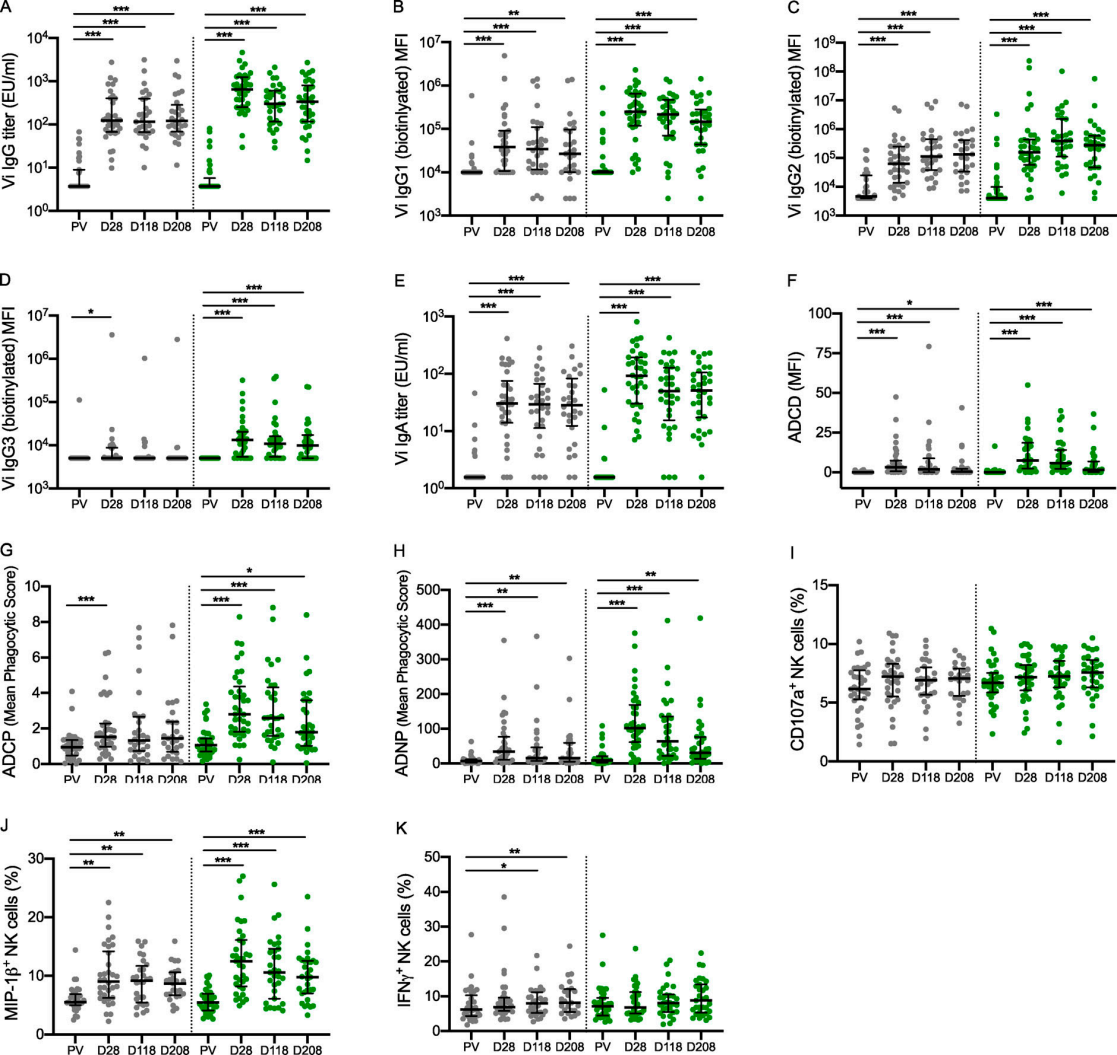
**Both Vi vaccines are immunogenic, inducing significantly higher quantities of antibodies and antibody-mediated innate cell responses after vaccination. **Comparison of prevaccination (PV) with postvaccination humoral responses (days 28, 118, and 208) in Vi-TT and Vi-PS vaccinees. **(A) **Total****Vi IgG titer. **(B) **Vi IgG1 quantity. **(C) **Vi IgG2 quantity. **(D) **Vi IgG3 quantity. **(E) **Vi IgA titer. **(F) **ADCD. **(G) **ADCP. **(H) **ADNP. **(I) **Antibody-induced CD107a expression by NK cells. **(J) **Antibody-induced MIP-1β expression by NK cells. **(K) **Antibody-induced IFNγ expression by NK cells. P values were calculated using Wilcoxon signed-rank test and adjusted for multiple testing using the Bonferroni method; *, P < 0.05; **, P < 0.01; ***, P < 0.001. Gray circles, Vi-PS; green circles, Vi-TT; number of participants included in each analysis is listed in Table S1; median and interquartile ranges are presented. Samples were run in duplicate for all assays. The mean value was calculated and used for statistical analyses.

Significantly higher Vi-specific humoral responses (antibody quantity and antibody-mediated functional activities [monocyte and neutrophil phagocytosis and complement deposition]) were induced 28 days after vaccination compared with baseline, for both Vi-TT and Vi-PS vaccinees (Fig. 2 and Table S1).

Capacity of Vi antibodies to induce innate immune effector functional activities was significantly higher 28 days following vaccination, with the exception of antibody-mediated activation of natural killer (NK) cells, as measured by CD107a expression and IFNγ release (Fig. 2, I and K). Of note, antibody-dependent neutrophil oxidative burst activity (ADNOB) was increased in comparison with baseline in Vi-TT but not Vi-PS vaccinees (Table S1 A).

Vi-specific antibody measures were sustained at levels significantly higher than baseline when assessed at 118 and 208 days for both Vi-TT and Vi-PS vaccinees, with the exception of Vi IgG3 and antibody-dependent cellular phagocytosis (ADCP), which returned to baseline levels by day 118 in Vi-PS vaccinees (Fig. 2).

### Vi-TT induces significantly higher fold increases in Vi-specific IgG responses than Vi-PS

**Figure 3.**
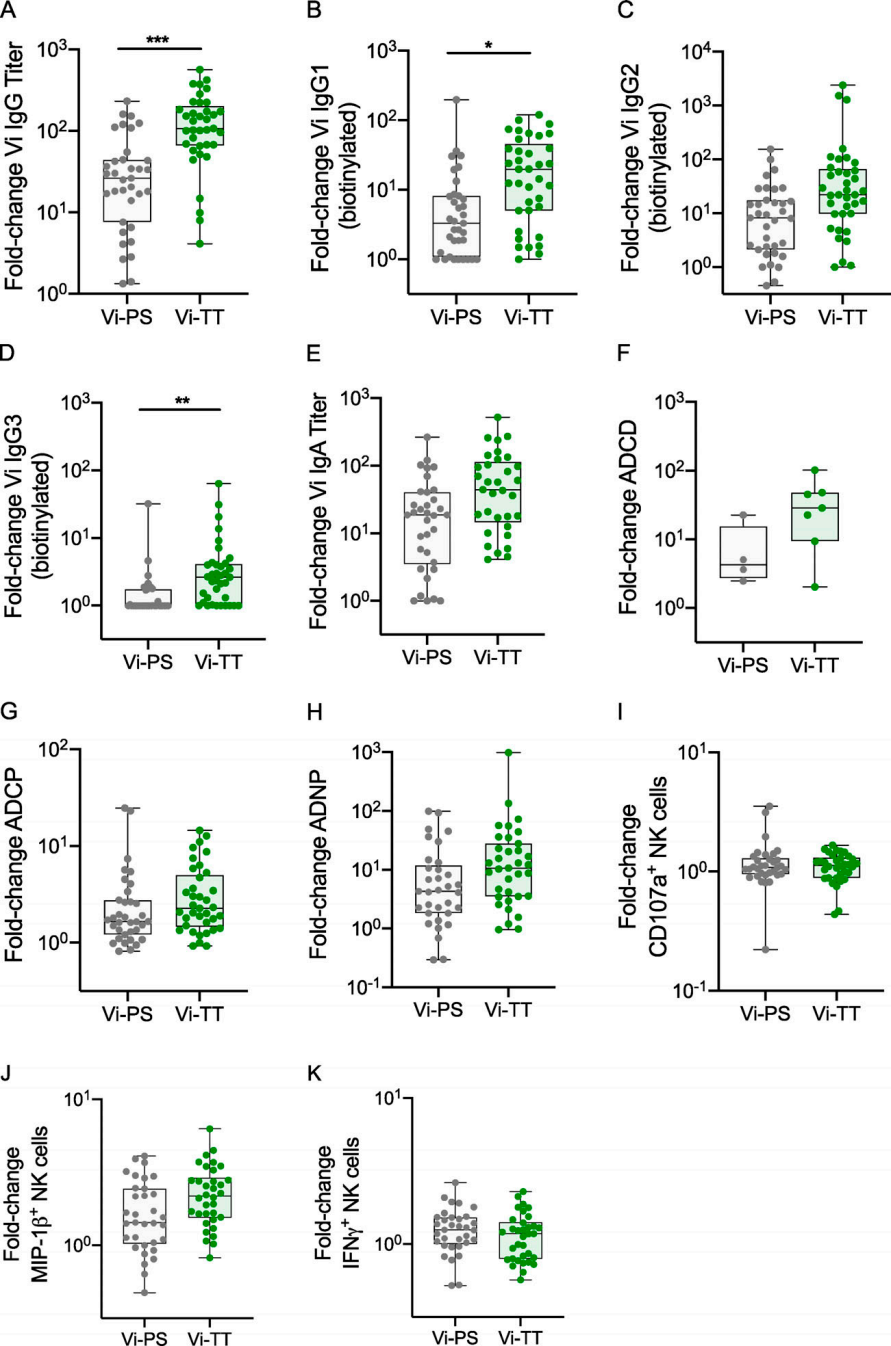
**Vi-TT induces higher-fold increases in Vi IgG responses than Vi-PS. **Comparison of fold changes in responses from baseline to day 28 between Vi-TT and Vi-PS groups. **(A) **Total****Vi IgG titer. **(B) **Vi IgG1 quantity. **(C) **Vi IgG2 quantity. **(D) **Vi IgG3 quantity. **(E) **Vi IgA titer. **(F) **ADCD. **(G) **ADCP. **(H) **ADNP. **(I) **Antibody-induced CD107a expression by NK cells. **(J) **Antibody-induced MIP-1β expression by NK cells. **(K) **Antibody-induced IFNγ expression by NK cells. P values were calculated using Mann–Whitney *U* test and adjusted for multiple testing using the Bonferroni method; *, P < 0.05; **, P < 0.01; ***, P < 0.001. Gray circles, Vi-PS; green circles, Vi-TT; number of participants included in each analysis is listed in Table S2; median and interquartile ranges are presented. Samples were run in duplicate for all assays. The mean value was calculated and used for statistical analyses.

Vi-TT induced significantly higher fold increases in total Vi IgG and IgG subclasses than Vi-PS 28 days after vaccination (Fig. 3, A, B, and D; and Table S2). However, by day 208, only Vi IgG3 responses remained significantly higher in Vi-TT versus Vi-PS vaccinees. Significantly higher fold increases in binding capacity of Fcγ receptors (FcγRs) at day 28 were also observed in Vi-TT vaccinees (Table S2).

Vi IgA1 subclass responses were higher in Vi-TT compared with Vi-PS vaccinees 28 days after vaccination; however, no significant differences in fold change of total IgA responses were observed (Fig. 3 E and Table S2). Furthermore, there was no difference in FcαR binding activity between the two vaccine groups at 28 days after vaccination. After adjusting for multiple testing, no significant differences were observed between Vi-TT and Vi-PS groups with regard to antibody avidity or measures of antibody-mediated functional activity after vaccination.

### Vi IgA titer correlates with protection from typhoid fever

**Figure S1.**
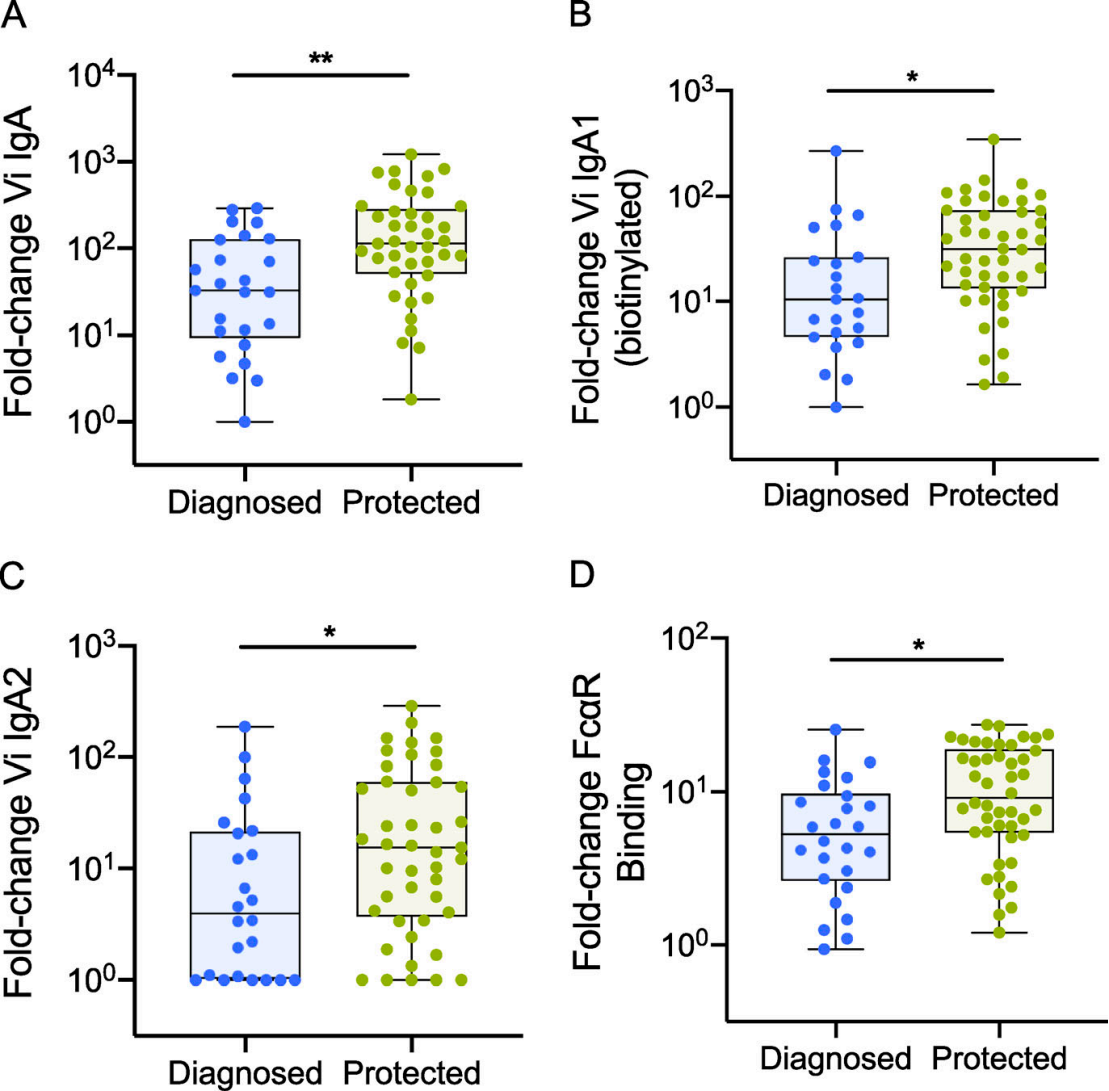
**Protected participants had higher fold rises in Vi IgA responses than diagnosed individuals (baseline to day 28). (A) **Fold change in total Vi IgA MFI. **(B) **Fold change in Vi IgA1 MFI. **(C) **Fold change in Vi IgA2 MFI. **(D) **Fold change in FcαR binding (using biotinylated Vi-PS). P values were calculated using Mann–Whitney *U* test; no adjustments for multiple testing were made; *, P < 0.05; **, P < 0.01. Median and interquartile ranges are presented.

Typhoid fever was diagnosed in 35% (13/37) of Vi-TT vaccinees, 37% (13/35) of Vi-PS vaccinees, and 77% (24/31) of control participants. To identify potential correlates of protection, the two Vi-vaccine groups were combined, and humoral responses were compared between diagnosed and protected participants for each postvaccination time point. Unadjusted univariate analyses identified higher absolute values on the day of challenge (day 28) and fold increases for most Vi IgA responses (total Vi IgA titer, IgA subclasses, and FcαR binding) in protected individuals compared with diagnosed participants (Fig. S1 and Table S3 A). Protected participants were also observed to have higher fold increases in total Vi IgG at all postvaccination time points (Table S4 A). Evaluation of functional antibody responses demonstrated significantly higher fold increases in ADNOB 28 days after vaccination in protected participants than diagnosed (P = 0.014) and a nonsignificant increase in antibody-dependent neutrophil phagocytosis (ADNP; P = 0.062; Table S4 A).

**Figure 4.**
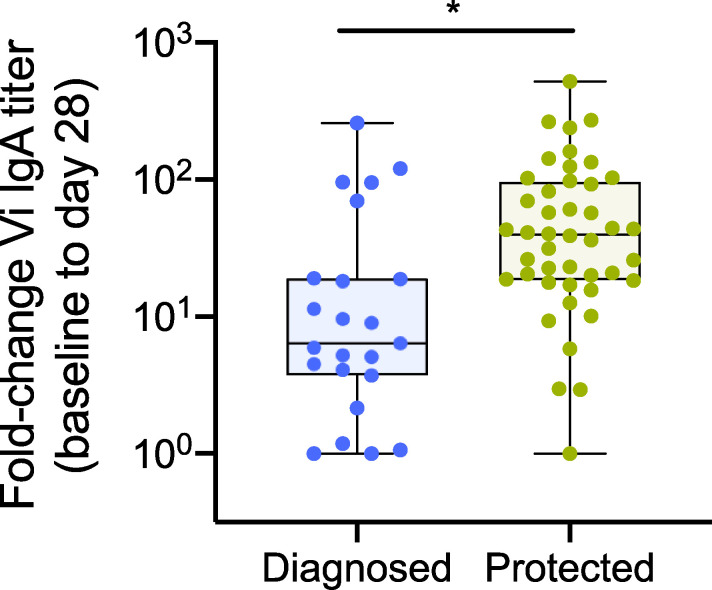
**Higher fold increase in Vi IgA titer correlates with protection from typhoid infection in Vi-vaccinated individuals challenged with *S. *Typhi. **Protected participants had significantly higher fold increases in Vi IgA titer from baseline to day 28 (day of challenge) than diagnosed individuals. P values were calculated using Mann–Whitney *U* test and adjusted for multiple testing using the Bonferroni method; *, P < 0.05. Median and interquartile ranges are presented.

Although no significant differences in absolute antibody titers were identified between diagnosed and protected participants after correcting for multiple testing (Table S3 B), individuals who were protected from typhoid infection had significantly higher fold increases in total Vi IgA titer from baseline to days 28, 118, and 208 compared with diagnosed participants (Fig. 4 and Table S4 B).

To identify differences in protective correlates between Vi-TT and Vi-PS vaccine groups, responses in diagnosed and protected individuals were compared based on vaccine allocation. After correcting for multiple testing, antibody-dependent NK cell activity (ADNKA) with IFNγ release was significantly higher in diagnosed than protected Vi-TT participants at baseline (P = 0.019; Table S5 A).

No significant differences in fold change of Vi-specific measures between diagnosed and protected individuals were observed when each vaccine group was assessed separately (Table S6). However, evaluation of differences in fold change before correcting for multiple testing identified higher fold increases in Vi IgA responses in protected individuals (Table S6). Higher fold increases in ADNOB activity and ADNKA (CD107a) on day 28 were also observed in protected individuals vaccinated with Vi-TT (unadjusted P = 0.037 and P = 0.027, respectively; Table S6 A).

### Vi IgA and IgG responses together predict protection from typhoid fever

Univariate analyses identified Vi IgA and potentially Vi IgG as mediators of protection. We hypothesized that a combination of features within the polyclonal Vi-specific humoral response were involved in mediating protection from *S*. Typhi infection. To further explore this, we used a supervised multivariate approach that combined least absolute shrinkage and selection operator (LASSO) with partial least squares discriminatory analysis (PLSDA) to define a minimal set of humoral features (Gunn et al., 2018) that, together, distinguished protected versus diagnosed vaccinees across the two vaccine arms. Given the correlated nature of the humoral immune measurements, additional features that were correlated to the LASSO-selected features were also examined to help understand the biological and potential mechanistic underpinnings of the identified correlates.

**Figure 5.**
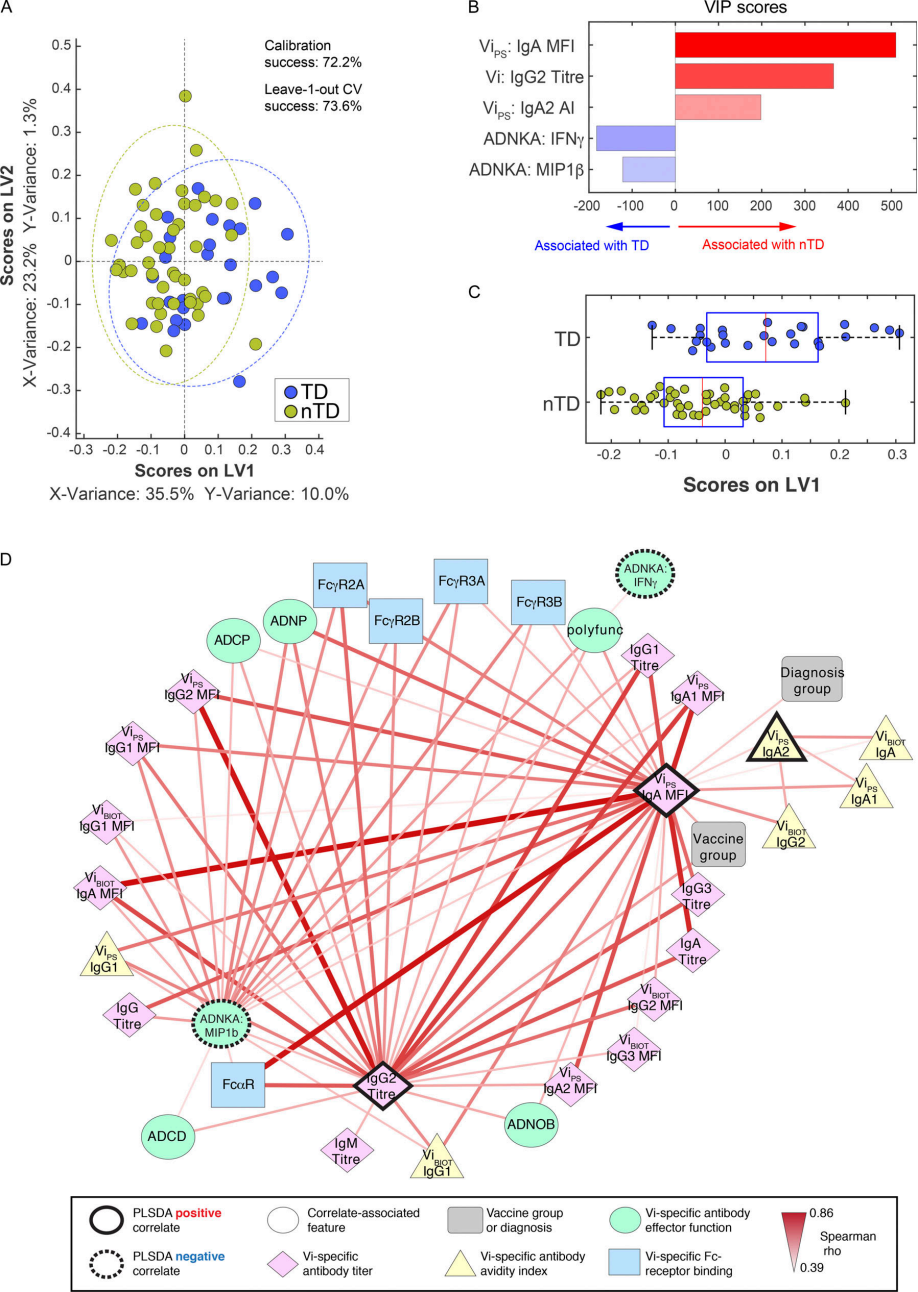
**Vi IgA and IgG responses at day 28 together predict protection from typhoid fever. (A) **LASSO feature selection followed by partial least-squares regression (PLSR) analysis defined****the five minimal antibody features needed to predict TD or nTD from *S. *Typhi challenge across both Vi-TT and Vi-PS vaccine groups. Each participant is color coded by diagnosis (blue, TD; gold, nTD) in the scoring plot, and the calibration success and leave-one-out cross validation is indicated on the plot. The features used for analysis are listed in Table 1. A total of 72 participants were included in the analysis (TD, *n* = 26; nTD, *n* = 46). **(B) **The five antibody features needed to predict TD were ranked by their VIP scores in the PLSR model. Features are color coded by the association with either diagnosis (blue bars) or no diagnosis (red bars). **(C) **Univariate analysis of LV1 scores between TD and nTD (median and interquartile ranges). **(D) **Co-correlation network analysis of antibody features associated with the LASSO-selected correlates. Each node represents the indicated antibody feature as described in the boxed legend. The connecting lines between features represent statistically significant Spearman ρ associations following Benjamini–Hochberg correction for false discovery rate (*q* < 0.01), and the strength of correlation is indicated by the weight of the connecting line. The ellipse defines the region that contains 95% of the data points distributed on the LV1 and LV2 space. Polyfunc, polyfunctionality.

Multivariate analyses identified a set of five shared features that predicted protection across both Vi-TT and Vi-PS vaccine groups at day 28: Vi IgA quantity (detected using the binding antibody multiplex assay [BAMA]; Dahora et al., 2019), Vi IgG2 titer (measured using ELISA), and IgA2 avidity were associated with protection, whereas ADNKA features (release of IFNγ and macrophage inflammatory protein-1β [MIP-1β]) were associated with infection (Fig. 5, A and B). Univariate comparisons of the latent variable (LV) scores across the most discriminatory axis, LV1, pointed to Vi IgA as the top feature that was selectively enriched among protected individuals (Fig. 5 C). Furthermore, network analysis highlighted a tightly interconnected network of features all linked to Vi IgA and IgG2, including Vi IgG1 and IgG3, and ADNOB activity (Fig. 5 D). Given the constitutive expression of both FcγRs and FcαR on neutrophils, these data point to a protective role for coordinated IgA- and IgG-mediated neutrophil activation as a potential mechanism underlying protective immunity for both vaccine groups. Moreover, the enrichment of ADNKA with other functions, including ADCP and antibody-dependent complement deposition (ADCD), among diagnosed volunteers points to a highly specialized and unique functional axis in which broad innate immune activation may be detrimental to control, whereas selective neutrophil activation may be key to protective antimicrobial activity.

**Figure S2.**
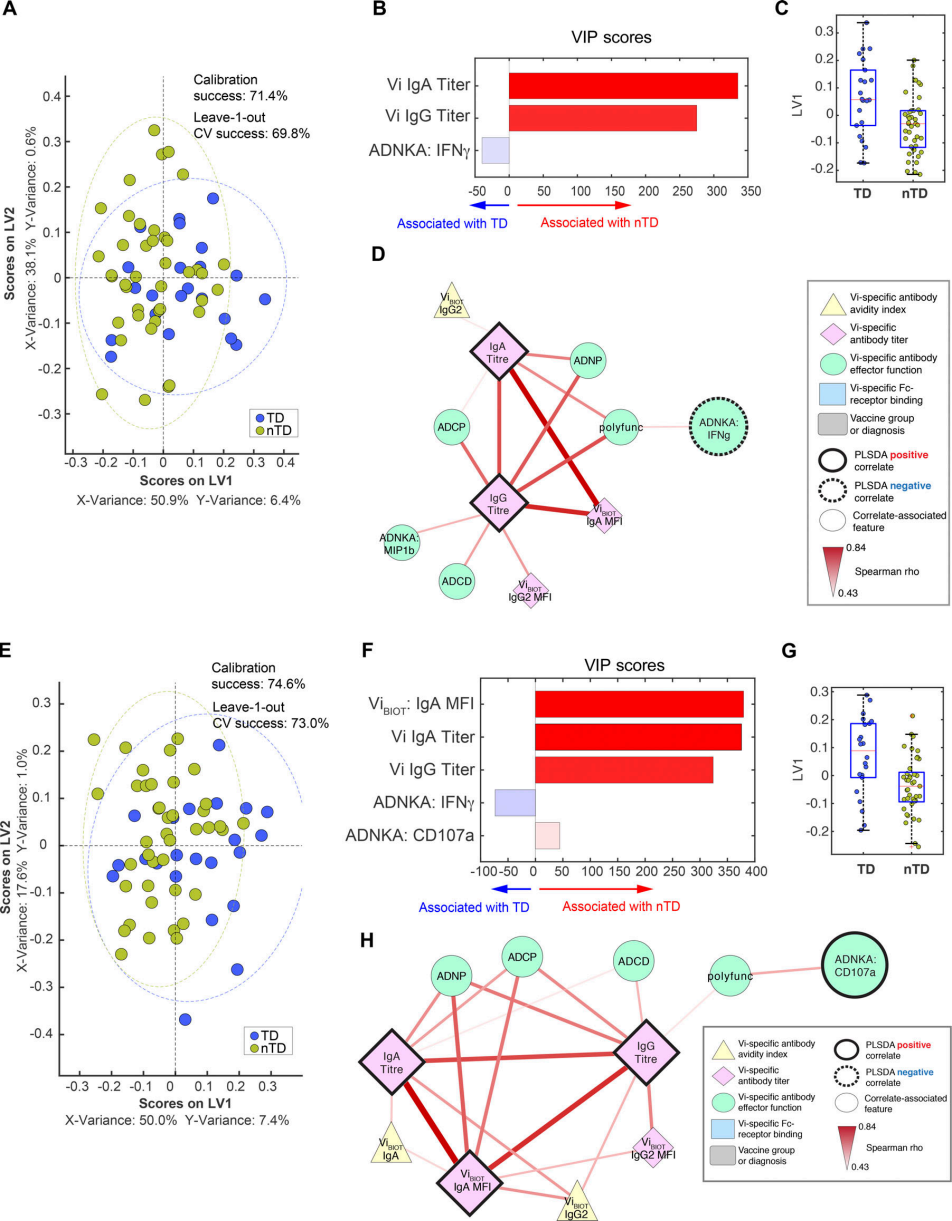
**Vi IgA and IgG remain elevated in protected participants up to 6 months after challenge.** **(A–H) **LASSO feature selection followed by partial least-squares regression (PLSR) analysis defined****the minimal antibody features needed to predict whether participants were TD or nTD from *S. *Typhi challenge across both Vi-TT and Vi-PS vaccine groups on day 118 (A–D) and day 208 (E–H) after challenge. Each participant is color coded by diagnosis (blue, TD; gold, nTD) in the scoring plot, and the calibration success and leave-one-out cross validation are indicated on the plot (A and E). The features used for analysis are listed in Table 1. A total of 72 participants were included in the analysis (TD, *n* = 26; nTD, *n* = 46). The antibody features needed to predict typhoid diagnosis were ranked by their VIP scores in the PLSR model. Features are color coded by the association with either diagnosis (blue bars) or no diagnosis (red bars; B and F). Univariate analysis of LV1 scores between TD and nTD (C and G; median and interquartile ranges). Co-correlation network analysis of antibody features associated with the LASSO-selected correlates (D and H). Each node represents the indicated antibody feature as described in the boxed legend. The connecting lines between features represent statistically significant Spearman ρ associations after Benjamini–Hochberg correction for false discovery rate (*q* < 0.01), and the strength of correlation is indicated by the weight of the connecting line. The ellipse defines the region that contains 95% of the data points distributed on the LV1 and LV2 space. Polyfunc, polyfunctionality.

Similar to the multivariate correlates identified at day 28, the Vi IgA levels measured at days 118 and 208 were elevated in protected participants, whereas ADNKA IFNγ release was a predictor of infection. While Vi IgG was not selected as a PLSDA correlate at day 28, the selection of IgG as a correlate after challenge suggests that Vi IgG may represent a marker of persistent protection in these individuals. Network analysis of the LASSO/PLSDA correlates demonstrated a concurrent increase in the ability of antibodies to induce effector functions, suggesting that long-term polyfunctional antibodies may be a signature of a protective antibody response against *S.* Typhi (Fig. S2).

While it might be hypothesized that exposure to *S.* Typhi bacteria after Vi vaccination would boost Vi humoral responses, we did not find any evidence to support this. Overall, most Vi humoral responses peaked on the day of challenge (day 28) and remained stable or waned by day 208 (Fig. 3). Previous typhoid CHIM studies also demonstrated that unvaccinated participants failed to produce Vi antibodies following oral challenge (Waddington et al., 2014). Together these observations suggest that, within the context of the CHIM, *S.* Typhi exposure did not induce boosting of humoral responses or interfere with the evaluation of serological correlates of protection at post-challenge time points.

### Vi-TT and Vi-PS vaccines induce distinct protective signatures

**Figure 6.**
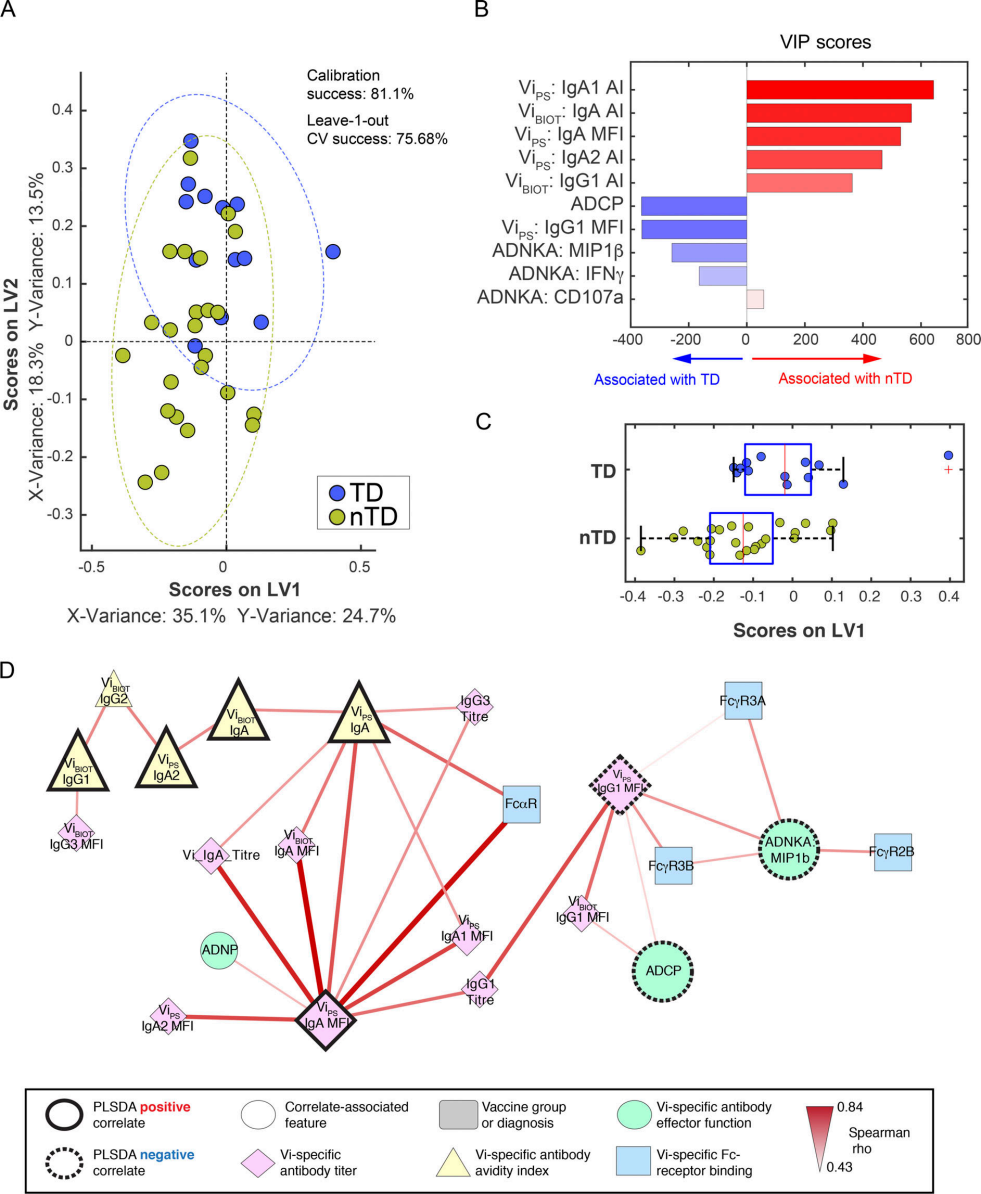
**Vi IgA, IgG, and activation of neutrophils measured on day 28 are correlates of protection mediated by Vi-TT vaccination. (A) **LASSO feature selection followed by partial least-squares regression (PLSR) analysis defined****the 10 minimal antibody features needed to predict TD or nTD from *S. *Typhi challenge following vaccination with Vi-TT. Each participant is color coded by diagnosis (blue, TD; gold, nTD) in the scoring plot, and the calibration success and leave-one-out cross validation are indicated on the plot. The features used for analysis are listed in Table 1. A total of 37 participants were included in the analysis (TD, *n* = 13; nTD, *n* = 24). **(B) **The 10 antibody features needed to predict typhoid diagnosis were ranked by their VIP scores in the PLSR model. Features are color coded by the association with either diagnosis (blue bars) or no diagnosis (red bars). **(C) **Univariate analysis of LV1 scores between TD and nTD (median and interquartile ranges). **(D) **Co-correlation network analysis of antibody features associated with the LASSO-selected correlates. Each node represents the indicated antibody feature as described in the boxed legend. The connecting lines between features represent statistically significant Spearman ρ associations after Benjamini–Hochberg correction for false discovery rate (*q* < 0.01), and the strength of correlation is indicated by the weight of the connecting line. The ellipse defines the region that contains 95% of the data points distributed on the LV1 and LV2 space.

**Figure S3.**
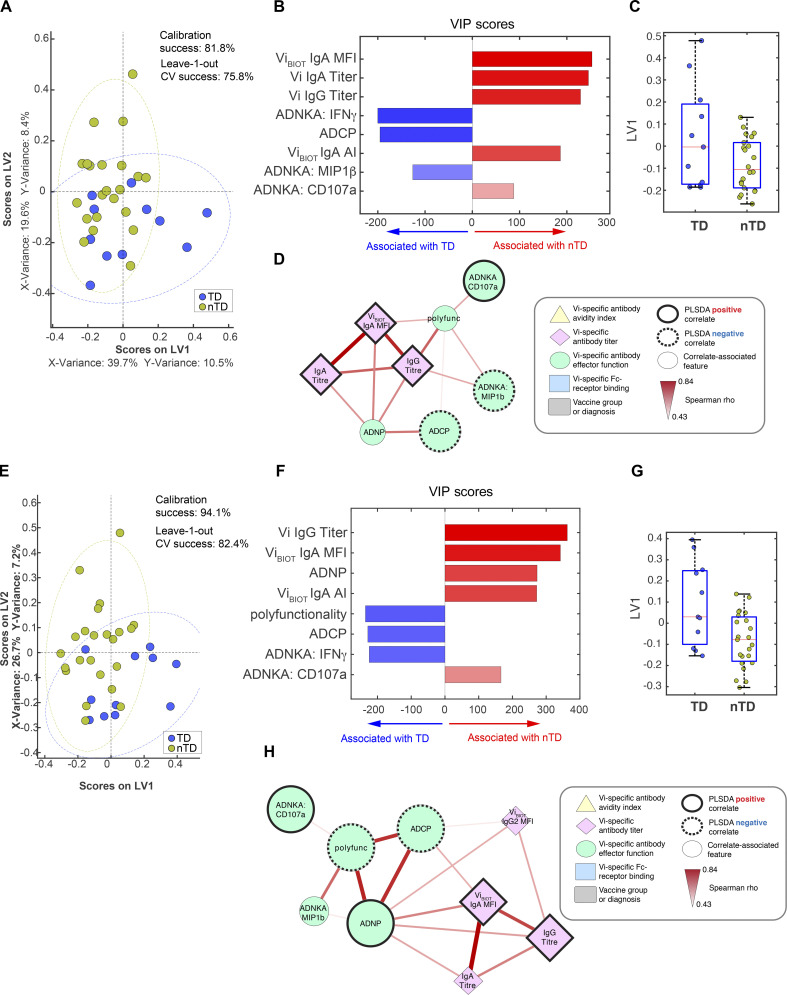
**Sustained Vi IgA, IgG, and ADNP activity mark protected participants vaccinated with Vi-TT. (A–H) **LASSO feature selection followed by partial least-squares regression (PLSR) analysis defined****the minimal antibody features measured on day 118 (A–D) and day 208 (E–H) after *S. *Typhi challenge that were needed to predict whether participants were TD or nTD from challenge after Vi-TT vaccination. Each participant is color coded by diagnosis (blue, TD; gold, nTD) in the scoring plot, and the calibration success and leave-one-out cross validation are indicated on the plot (A and E). The features used for analysis are listed in Table 1. A total of 37 participants were included in the analysis (TD, *n* = 13; nTD, *n* = 24). The antibody features needed to predict typhoid diagnosis were ranked by their VIP scores in the PLSR model. Features are color coded by the association with either diagnosis (blue bars) or no diagnosis (red bars; B and F). Univariate analysis of LV1 scores between TD and nTD (C and G; median and interquartile ranges). Co-correlation network analysis of antibody features associated with the LASSO-selected correlates (D and H). Each node represents the indicated antibody feature as described in the boxed legend. The connecting lines between features represent statistically significant Spearman ρ associations after Benjamini–Hochberg correction for false discovery rate (*q* < 0.01), and the strength of correlation is indicated by the weight of the connecting line. The ellipse defines the region that contains 95% of the data points distributed on the LV1 and LV2 space. Polyfunc, polyfunctionality.

Distinct protective signatures were identified for Vi-TT and Vi-PS when each vaccine group was evaluated separately. A set of 10 features were needed to predict protection within participants vaccinated with Vi-TT, and interestingly, protection was primarily associated with Vi IgA responses (IgA quantity and avidity) and IgG1 avidity (Fig. 6, A and B). Again, infection was associated with elevated ADCP, Vi IgG1 quantity, and MIP-1β release from NK cells (Fig. 6, A and D), which were all linked within the network analysis. Degranulation of NK cells (CD107a surface expression) was also associated with protection, suggesting that cytotoxic activity by NK cells, but not proinflammatory activities, may contribute to protection. The association of Vi IgG1 avidity with protection may suggest that a subset of Vi IgG1 antibodies capable of inducing NK cell degranulation and neutrophil activity may be protective. Protected participants continued to maintain or develop antibodies capable of inducing ADNP, as ADNP activity remained elevated in sera of protected Vi-TT vaccinees up to day 208, in concert with elevated Vi IgG and IgA titers (Fig. S3).

**Figure 7.**
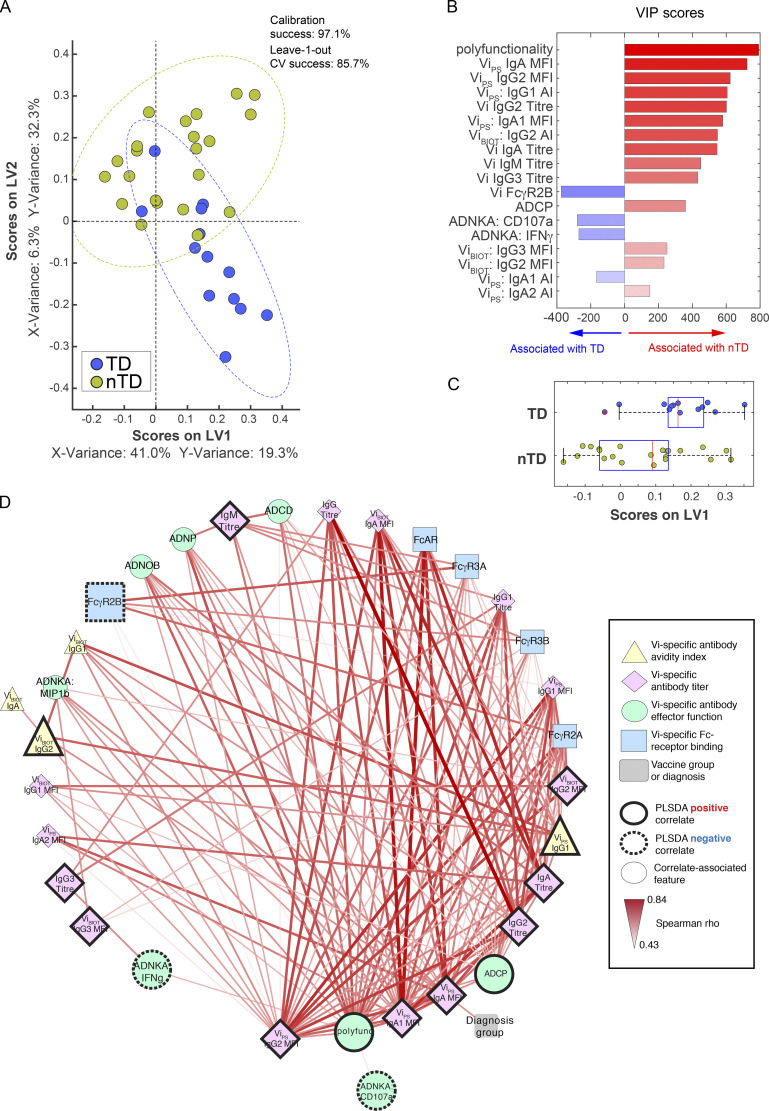
**Polyisotypic and polyfunctional responses measured on day 28 are correlates of protection mediated by Vi-PS vaccination. (A) **LASSO feature selection followed by partial least-squares regression (PLSR) analysis defined****the 18 minimal antibody features needed to predict TD or nTD from *S. *Typhi challenge after vaccination with Vi-PS. Each participant is color coded by diagnosis (blue, TD; gold, nTD) in the scoring plot, and the calibration success and leave-one-out cross validation are indicated on the plot. The features used for analysis are listed in Table 1. A total of 35 participants were included in the analysis (TD, *n* = 13; nTD, *n* = 22). **(B) **The 18 antibody features needed to predict typhoid diagnosis were ranked by their VIP scores in the PLSR model. Features are color coded by the association with either diagnosis (blue bars) or no diagnosis (red bars). **(C) **Univariate analysis of LV1 scores between TD and nTD (median and interquartile ranges). **(D) **Co-correlation network analysis of antibody features associated with the LASSO-selected correlates. Each node represents the indicated antibody feature as described in the boxed legend. The connecting lines between features represent statistically significant Spearman ρ associations after Benjamini–Hochberg correction for false discovery rate (*q* < 0.01), and the strength of correlation is indicated by the weight of the connecting line. The ellipse defines the region that contains 95% of the data points distributed on the LV1 and LV2 space. Polyfunc, polyfunctionality.

**Figure S4.**
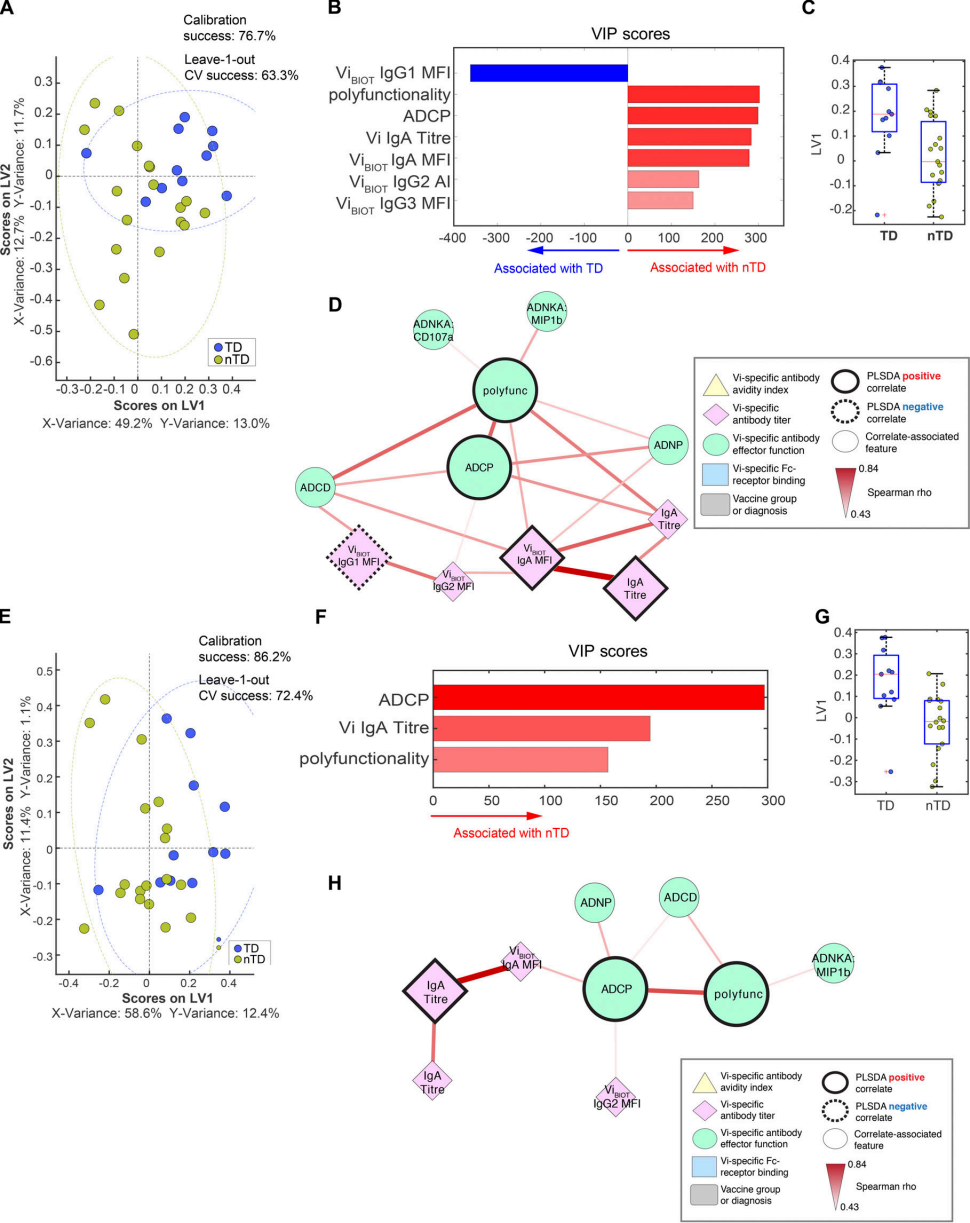
**Sustained polyisotypic and polyfunctional antibodies remain as correlates of protection for participants vaccinated with Vi-PS. (A–H) **LASSO feature selection followed by partial least-squares regression (PLSR) analysis defined****the minimal antibody features measured on day 118 (A–D) and day 208 (E–H) after *S. *Typhi challenge that were needed to predict whether participants were TD or nTD from challenge after Vi-PS vaccination. Each participant is color coded by diagnosis (blue, TD; gold, nTD) in the scoring plot, and the calibration success and leave-one-out cross validation are indicated on the plot (A and E). The features used for analysis are listed in Table 1. A total of 35 participants were included in the analysis (TD, *n* = 13; nTD, *n* = 22). The antibody features needed to predict typhoid diagnosis were ranked by their VIP scores in the PLSR model. Features are color coded by the association with either diagnosis (blue bars) or no diagnosis (red bars; B and F). Univariate analysis of LV1 scores between TD and nTD (C and G; median and interquartile ranges). Co-correlation network analysis of antibody features associated with the LASSO-selected correlates (D and H). Each node represents the indicated antibody feature as described in the boxed legend. The connecting lines between features represent statistically significant Spearman ρ associations after Benjamini–Hochberg correction for false discovery rate (*q* < 0.01), and the strength of correlation is indicated by the weight of the connecting line. The ellipse defines the region that contains 95% of the data points distributed on the LV1 and LV2 space. Polyfunc, polyfunctionality.

While distinct, similar thematic correlates were observed in Vi-PS vaccinated participants. Protection in Vi-PS vaccinees appeared to be driven by a polyisotypic Vi antibody response, marked by elevated levels of Vi-specific IgA, IgG, and IgM (Fig. 7). Furthermore, polyfunctionality (i.e., antibodies capable of inducing multiple functional responses such as complement deposition and neutrophil and cellular phagocytosis) was enriched among protected individuals (Fig. 7), highlighting the broader functional response required to drive protection following Vi-PS vaccination. Polyfunctionality remained an important signature of protective immunity up until day 208. Vi IgA and ADCP were also identified as features associated with protection in Vi-PS vaccinees at this later time point (Fig. S4).

### Higher fold increases in Vi IgG titer are associated with reduced inflammatory responses and bacterial burden in diagnosed vaccinees

**Figure 8.**
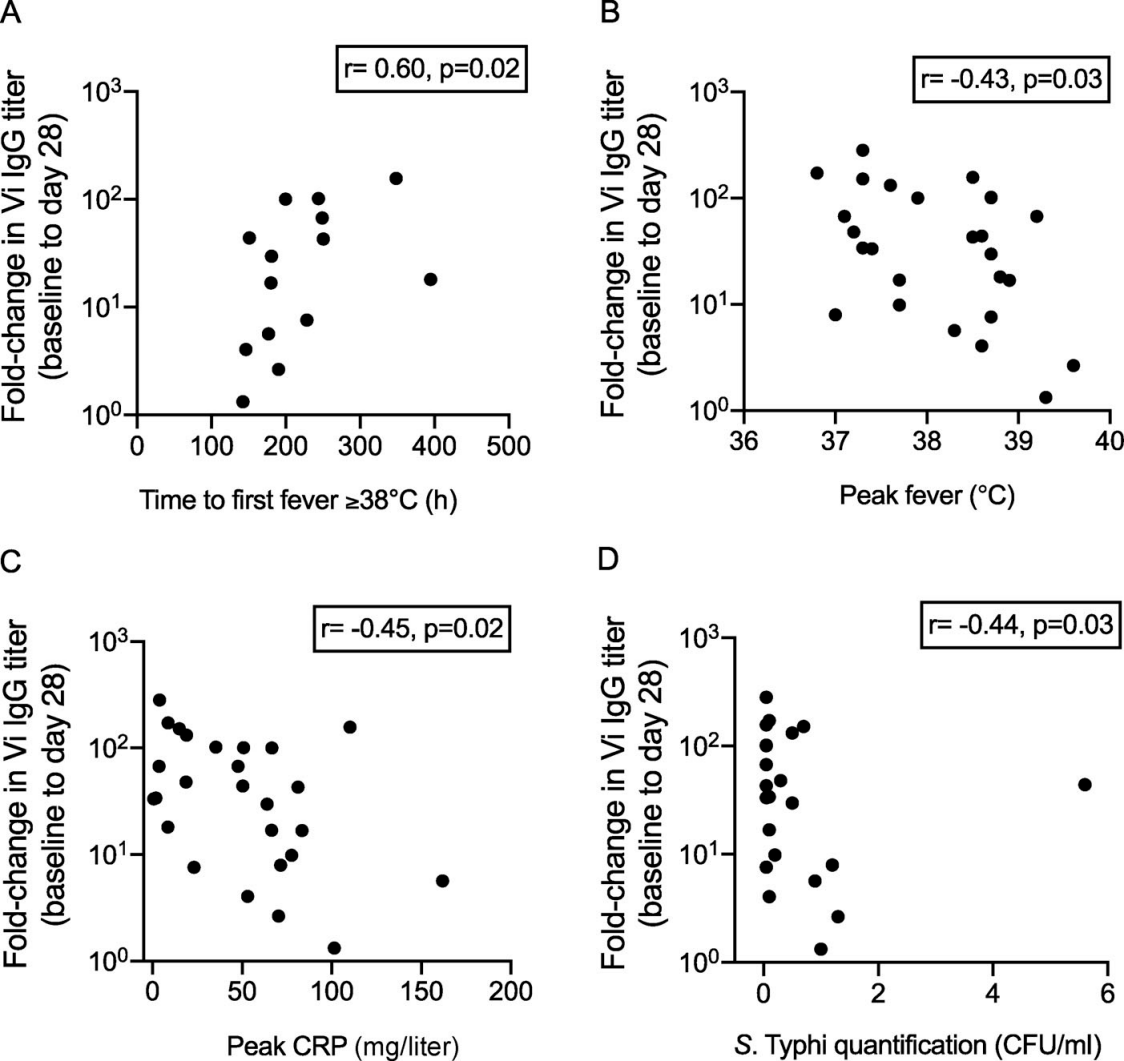
**Fold change in IgG titer from baseline to day 28 correlates with reduced inflammatory responses and bacterial burden in diagnosed participants.**(A) **Fold change in Vi IgG titer positively correlated with time to first fever ≥38°C. **(B) **Fold change in Vi IgG titer negatively correlated with peak recorded fever. **(C) **Fold change in IgG titer negatively correlated with peak CRP. **(D) **Fold change in IgG titer negatively correlated with *S. *Typhi quantification (bacterial burden measured at the time of typhoid fever diagnosis. TD participants in Vi-TT and Vi-PS vaccine groups were combined for analysis; P values were not adjusted for multiple testing.

Exploratory analyses were undertaken to investigate potential correlations between protective signatures, identified from univariate and multivariate analyses, and clinical and laboratory parameters. A positive association between fold increase in Vi IgG titer from baseline to day 28 and time to first fever ≥38°C was observed (Spearman ρ = 0.60, P = 0.02). Fold-increase in Vi IgG titer also negatively correlated with peak recorded temperature (Spearman ρ = −0.43, P = 0.03), peak C reactive protein (CRP; Spearman ρ = −0.45, P = 0.02), and *S.* Typhi bacterial burden in blood (Spearman ρ = −0.44, P = 0.03) in diagnosed participants (Fig. 8 and Table S7). No significant correlations between fold change in Vi IgA titer from baseline to day 28, clinical outcomes, or microbiological outcomes were observed. However, there were nonsignificant positive associations between fold change in Vi IgA titer and time to first fever ≥38°C (Spearman ρ = 0.55, P = 0.05) and time to first positive *S*. Typhi stool culture (Spearman ρ = 0.50, P = 0.07; Table S7).

No significant correlations between fold change in Vi IgG or Vi IgA, time to typhoid fever diagnosis, or time to detection of *S*. Typhi bacteremia were identified. However, a nonsignificant positive association between fold increase in Vi IgG and time to first positive *S*. Typhi blood culture was observed (Spearman ρ = 0.37, P = 0.07).

## Discussion

This study represents the first comprehensive evaluation of Vi-vaccine correlates of protection performed using samples obtained from an *S*. Typhi human infection model. Using a systems serology approach, we integrated a range of vaccine-induced humoral measures to evaluate co-correlate networks and identified multivariate protective humoral signatures that may have been overlooked with traditional univariate methods. Our findings suggest that protection from typhoid fever is mediated through the synergistic effects of the polyclonal antibody response, involving both qualitative antibody components (avidity and innate immune cell functional responses) and Vi IgA and IgG quantity.

Protection in Vi-PS vaccinees was associated with a generalized polyisotypic response (IgA, IgM, and all subclasses of IgG). In contrast, protection associated with Vi-TT vaccination was more selective, involving only Vi IgA responses (quantity and avidity) and Vi IgG1 avidity. These findings likely reflect differences in the underlying immune mechanisms elicited by T-independent PS vaccines (extrafollicular B cell responses) versus T-dependent glycoconjugate vaccines (germinal center formation).

Of note, protection in both vaccine groups was associated with antibody-mediated functional activity. While protective functions induced by Vi-TT vaccination were more restricted, primarily involving neutrophil phagocytosis, a broader range of functional activities, including ADNP, was observed in protected Vi-PS vaccinees. These findings suggest that neutralizing antibodies alone are insufficient to prevent *S*. Typhi infection and could account for the difficulties in identifying antibody “thresholds of protection” (Jin et al., 2017; Dahora et al., 2019). Similar observations have been made following parenteral malaria vaccination, in which different vaccine strategies (adenoviral vaccine prime with a protein boost versus protein vaccination alone) resulted in comparable protective efficacies despite the induction of different antibody titers (Ockenhouse et al., 2015).

As we have previously described, Vi IgA responses were associated with protection in both vaccine groups (Dahora et al., 2019). Although the protective role of secretory IgA has been well described for certain enteropathogenic viruses (e.g., rotavirus; Coulson et al., 1992; Matson et al., 1993), and nonenteropathogenic viruses (e.g., poliovirus; Buisman et al., 2008), few studies have evaluated IgA titers following parenteral Vi-vaccination (Chernokhvostova et al., 1969; Kossaczka et al., 1999). As such, the mechanisms by which Vi IgA mediates protection are unknown. During the early stages of infection, the periods when *S.* Typhi bacteria are extracellular and expressing the Vi-PS capsule are limited (Watson and Holden, 2010). Vi capsular expression does not occur within the gut lumen owing to the high osmolarity environment (Hu et al., 2017); as such, secretory Vi IgA or exudative IgG are unlikely to prevent *S.* Typhi invasion. We postulate that high concentrations of Vi IgA present in local mucosal sites, such as the lamina propria, Peyer’s patches, or efferent lymphatics, are responsible for opsonizing Vi-expressing extracellular *S*. Typhi, thereby preventing the establishment of infection.

Several studies have reported mechanisms of *S.* Typhi killing mediated by Vi IgG, such as complement-mediated serum bactericidal activity and phagocyte-mediated killing (Pulickal et al., 2009; Hart et al., 2016), but none have investigated the role of Vi IgA on downstream effector responses. Univariate analyses of Fc-mediated effector responses from this study have shown a significant increase in antibody-mediated complement deposition, neutrophil phagocytosis, and cellular (monocyte) phagocytosis following Vi-TT and Vi-PS vaccination. However, these functional assays were performed using sera containing polyclonal Vi antibodies and not purified Vi IgA. Co-correlate network analyses identified an association between neutrophil activity (phagocytosis and oxidative burst) and Vi IgA and IgG2 with protection. While this suggests that neutrophils are important mediators of protection, further studies are required to investigate the mechanistic interactions between Vi vaccine–induced IgA and neutrophil effector functions such as the NADPH-dependent oxidative burst, degranulation, and release of antimicrobial enzymes, and perhaps even the release of neutrophil extracellular traps.

Human challenge models are useful experimental settings for evaluating vaccine correlates of protection. They provide opportunities for intensive blood sampling and collection of clinical data in the context of timed interventions (e.g., vaccination and challenge). However, many aspects of natural *S*. Typhi infection cannot be reproduced within a challenge model setting. As such, there are differences in study population (children vs. adults), time interval between vaccination and challenge, challenge inoculum, and *S*. Typhi strain. The limitations of CHIM studies are numerous and a consequence of logistical factors, in addition to ethical and safety considerations. The sample size of this study was calculated based on efficacy outcomes and was not powered to specifically evaluate correlates of protection. This has affected the statistical interpretation of our results, as many findings were nonsignificant after adjusting for multiple testing.

The administered challenge inoculum (1–5 × 10^4^ CFU) and 14-day study design also affect the identification of correlates of protection. The administered challenge dose of *S.* Typhi was calculated to produce an attack rate of 60–75% in naive participants (Waddington et al., 2014). The typhoid fever attack rate within our control group was 77%. It is unknown whether the remaining 23% of individuals were protected from infection by the presence of preexisting immunity or innate immune responses, or whether they would have succumbed to typhoid infection if treatment were delayed. It is reasonable to assume that a proportion of nondiagnosed individuals, within both Vi vaccine groups, fall under the same category as the nondiagnosed control participants. Inclusion of this group of individuals within our analyses may have reduced the signal of certain protective humoral responses.

We recognize that some of these limitations affect the generalizability of our findings to relevant endemic settings. In particular, definitions used to identify typhoid fever cases differ between phase III efficacy trials (which identify participants with typhoid fever disease, i.e., febrile or symptomatic participants with *S*. Typhi bacteremia) and CHIM studies. Our composite definition facilitated detection of all participants with typhoid fever infection, including both those with disease (febrile participants) and those who were infected without clinical manifestations (asymptomatic *S*. Typhi bacteremia). We were unable to perform any correlate analyses using a typhoid fever disease definition, similar to that used in field efficacy trials (fever ≥38°C followed by positive *S*. Typhi culture), because of the small sample size of our study (Vi-PS diagnosed, *n* = 7/35; Vi-TT diagnosed, *n* = 2/37, using the above definition).

However, our analyses did identify an association between higher fold increases in Vi IgG titer and delayed time to first fever, lower peak temperatures, and lower CRP levels. We hypothesize that Vi IgG controls bacterial burden after *S.* Typhi are released into the bloodstream during bacteremic phases of infection, thereby reducing clinical manifestations of disease. This is supported by the observation that higher fold increases in Vi IgG titer were associated with lower numbers of *S.* Typhi bacteria detected from blood at the time of typhoid fever diagnosis. Conversely, no correlations between fold change in Vi IgA titer and surrogate clinical outcomes were observed. Vi-TT induced significantly higher Vi IgG titers than Vi-PS, which may provide an explanation for the reduction in fever and severity of symptoms observed in diagnosed Vi-TT vaccinees compared with their Vi-PS counterparts (Jin et al., 2017). Of note, no significant differences in fold increase in Vi IgA titers were observed between vaccine groups, which may explain why equivalent efficacies for Vi-TT and Vi-PS were observed in the CHIM efficacy trial, as an *S.* Typhi infection endpoint rather than typhoid fever disease endpoint was used to measure vaccine efficacy. In summary, these findings may indicate that Vi IgA responses are important for preventing *S.* Typhi infection, therefore representing a correlate of protection for infection, while Vi IgG responses reduce clinical manifestations of disease after infection has been established, supporting the concept of Vi IgG as a correlate of protection for typhoid fever disease.

It is important to recognize that the pathogenesis of typhoid fever differs from invasive disease caused by other encapsulated bacteria, for which serological correlates of protection have been successfully identified (e.g., *Haemophilus influenzae* type B, *Neisseria meningitidis*, and certain serotypes of *Streptococcus pneumoniae*). As discussed above, *S*. Typhi is primarily an intracellular pathogen. Infection results from several invasion events, and unlike other encapsulated bacteria, early bloodstream invasion with high numbers of bacteria does not occur. This may explain the complex relationship between Vi humoral responses detected in serum and protection against *S.* Typhi infection observed in our study. Murine models have also identified direct dissemination of *Salmonella* Typhimurium from the gut lumen to secondary lymphoid organs via CD18-expressing macrophages, suggesting that *Salmonella* bacteria could potentially bypass vaccine-induced antibodies altogether by remaining within protected intracellular environments (Vazquez-Torres et al., 1999).

The past few years represent a critical turning point for typhoid fever disease control. The ongoing outbreak caused by an extensively drug-resistant *S.* Typhi clone in Pakistan is a reminder of the catastrophic effects uncontrolled *S*. Typhi spread can have on resource-limited countries (Klemm et al., 2018; Rasheed et al., 2019). Although strategies to control disease burden using TCV programs are underway (Andrews et al., 2019), efforts that could assist with the acceleration of these vaccination programs should be prioritized. The findings from this study are an exciting development in TCV research and have increased our understanding of protective Vi vaccine–mediated humoral responses against *S.* Typhi infection and disease, which could in turn be used to evaluate TCVs in development and guide future vaccine design. Further studies are required, including the assessment of Vi IgA and IgG responses in children vaccinated with TCVs from typhoid-endemic settings, as responses may differ, and evaluation of antibody-mediated effector responses, in particular neutrophil activity, which will assist with elucidating mechanistic correlates of protection.

## Materials and methods

### Participant samples and study design

Samples were obtained from consenting adult volunteers participating in a phase IIb, participant-observer blinded, randomized, controlled, Vi-vaccine efficacy study conducted at the Centre for Clinical Vaccinology and Tropical Medicine (Churchill Hospital, Oxford, UK; ClinicalTrials.gov: NCT02324751), as previously described (Jin et al., 2017). Briefly, participants were randomized and vaccinated with Vi-TT (Typbar-TCV; Bharat Biotech), Vi-PS (Typhim Vi; Sanofi Pasteur), or meningococcal-ACWY conjugate vaccine (MENVEO; GlaxoSmithKline). Approximately 28 days after vaccination, participants ingested ∼10^4^ CFU of *S.* Typhi (Quailes strain). Blood and stool cultures were sampled daily over the 14-d challenge period; oral temperatures (recorded two to three times a day) and symptoms were reported for a total of 21 days after challenge. Typhoid fever was diagnosed in participants with positive *S*. Typhi bacteremia or fever ≥38°C for ≥12 h.

Humoral responses were evaluated for Vi vaccinees. Serum samples were collected before vaccination (baseline), on the day of challenge (day 28), and 4 months (day 118 ± 14) and 7 months (day 208 ± 28) after vaccination and stored at −80°C.

### Outcomes

The primary outcome of this study was to identify potential correlates of protection after Vi-TT and Vi-PS vaccination by comparing responses measured before participants were exposed to *S.* Typhi, on either day 7 after vaccination or day 28 (the day of challenge), in diagnosed versus protected participants. Secondary outcomes of this study included assessing vaccine immunogenicity, by comparing baseline and postvaccination responses, comparing responses between Vi-TT and Vi-PS groups, and assessing the persistence of protective signatures ≤208 days after vaccination. Exploratory analyses were performed to evaluate whether specific protective signatures correlated with other clinical or microbiological outcomes such as time to diagnosis, *S*. Typhi bacteremia, fever ≥38°C, *S.* Typhi stool shedding, disease severity characterized using peak CRP or temperature, or disease burden characterized as bacterial burden in blood (measured at the time of diagnosis).

### Assays used to evaluate Vi-specific responses

A collection of 35 assays was used to evaluate Vi-specific humoral and cellular responses (Table 1). Some assays were not performed for all time points owing to limited sample availability.

#### Vi antibody quantification

Vi IgG titers were measured using a commercial ELISA kit (VaccZyme; The Binding Site) according to the manufacturer’s guidelines. Vi IgG subclass titers (IgG1, IgG2, and IgG3) and isotypes IgA and IgM were assessed with Vi-coated plates and reagents supplied by the Binding Site using a protocol adapted from the commercial VaccZyme assay.

#### BAMA and avidity index (BAMA-AI)

Magnitude and avidity of polyclonal antibody responses to the Vi-PS were measured using the BAMA-AI assay as previously described (Dahora et al., 2019). Briefly, Luminex microspheres conjugated to either biotinylated (Vi_Biot_) or unbiotinylated (Vi_PS_) Vi-PS (12/244, lot 2039; NIBSC) were incubated with diluted vaccinee serum or plasma for 1 h. Immune complexes were then incubated with either PBS or a dissociative buffer (pH 3, sodium citrate) for 15 min followed by detection with fluorescent, subclass-specific secondary antibodies (anti-human IgG1, IgG2, IgG3, IgA1, and IgA2) for 30 min. FI was measured using the Bio-Plex 200 Platform. Typhoid seronegative serum samples and normal human serum (NHS; Sigma-Aldrich) were used as negative controls, and blank microspheres were used to control for nonspecific binding to beads by subtracting FI reading of blank beads from FI reading of Vi-conjugated beads. AI was calculated as

$$AI=\left[\frac{FI-\mathrm{background}\;\left(CIT\right)}{FI-\mathrm{background}\;\left(PBS\right)}\right]\times 100,$$

expressed as a percentage. The magnitude of polyclonal responses was multiplied by sample dilution factor. Fold change was calculated by the ratio of response magnitude at day 28, 118, or 208 to baseline (day 0). AI was calculated only for positive vaccine responders; criteria for positivity included mean fluorescence intensity (MFI) × dilution > 95th percentile of baseline, MFI > 100, and MFI × dilution > threefold over subject-specific baseline MFI × dilution.

#### Functional antibody responses

Methods used to measure (ADCD; Barouch et al., 2015), ADCP (Ackerman et al., 2011), and ADNP (Barouch et al., 2015) were performed as previously described. MFIs were reported after subtraction of background. Results were calculated from the mean result of sample replicates across two assay runs. Data were acquired on an S1000EXi cytometer (Stratedigm) and analyzed using FlowJo v10.6 software.

### ADCD

Heat-inactivated serum samples were diluted 1:10 and incubated at 37°C for 2 h with red fluorescent NeutrAvidin-coated microspheres (Thermo Fisher Scientific) coated with biotinylated Vi-antigen (provided by Dr. Sjoerd Rijpkema, NIBSC, Potters Bar, UK) in 96-well round-bottom plates. Guinea pig complement (Cedarlane), reconstituted in veronal buffer with 0.1% gelatin, was added and incubated at 37°C for a further 20 min. Beads were washed twice with 15 mM EDTA in PBS. FITC-conjugated goat anti–guinea pig complement C3b antibody (MP Biomedical) was added and incubated in the dark for 15 min at room temperature. Beads were washed, resuspended in PBS, and analyzed using flow cytometry. FITC MFIs were measured for each sample.

### ADCP

ADCP was assessed by quantifying phagocytosis of antibody-opsonized Vi-coated fluorescent microspheres using monocyte cell line THP-1 (IB-202; American Type Culture Collection). Serum samples were diluted 1:250 and incubated with NeutrAvidin-coated yellow-green 1-µm fluorescent microspheres coupled with biotinylated Vi-antigen. 2.5 × 10^4^ THP-1 cells (American Type Culture Collection) were added to each well and incubated overnight at 37°C. Cells were pelleted, fixed with 4% paraformaldehyde (PFA), and analyzed using flow cytometry. A phagocytic score was calculated using the mean MFI for each sample and the following formula: [(% microsphere-positive cells) × (MFI of microsphere-positive cells)]/10,000.

### ADNP

ADNP was assessed by measuring the uptake of antibody-opsonized Vi-coated fluorescent microspheres using freshly isolated neutrophils. Serum samples, diluted 1:100, were incubated with NeutrAvidin-coated yellow-green 1-µm fluorescent microspheres coupled with biotinylated Vi-antigen. Leukocytes were isolated from acid citrate dextrose–treated fresh blood from healthy anonymized donors using ammonium-chloride-potassium lysis buffer. 5 × 10^4^ leukocytes were added to each well containing antibody-opsonized beads and incubated for 1 h at 37°C. Cells were pelleted, the supernatant was removed, and cells were stained with APC-Cy7 anti-CD14 (clone MψP9; BD Biosciences) and Pacific Blue anti-CD66b (clone G10F5; BioLegend). Cells were washed, fixed with 4% PFA, and analyzed using flow cytometry. The phagocytic score was calculated from gated neutrophils (CD14^−^, CD66b^+^).

### Antibody-dependent NK cell activation

96-well ELISA plates were coated with streptavidin (5 µg/ml) at 37°C overnight. The next day, streptavidin solution was removed, and biotinylated Vi (3 µg/ml) was added to plates before incubation at 37°C for 3 h. Plates were washed with PBS, and serum samples diluted 1:10 were added to Vi-coated wells before incubation for 2 h at 37°C. After incubation, plates were washed with PBS before addition of NK cells. NK cells were isolated from buffy coats using a RosetteSep NK cell enrichment kit (Stem Cell Technologies), and a cocktail of PE-Cy5 anti-CD107a (clone H4A3), brefeldin A (Sigma-Aldrich), and GolgiStop (BD Biosciences) was added immediately before addition of NK cells to plate wells at 5 × 10^4^ cells/well. Plates were then incubated at 37°C for 5 h. After incubation, cells were transferred to a 96-well U-bottom plate containing an antibody surface stain cocktail of PE-Cy7 anti-CD56 (clone B159), APC-Cy7 anti-CD16 (clone 3G8), and A700 anti-CD3 (clone UCHT1) and incubated for 15 min. Cells were then washed before permeabilization with PermA (Life Technologies); intracellular staining for 15 min with MIP-1β-PE (clone D21-1351), IFNγ-APC (clone B27), and PermB (Life Technologies); and finally washing with PBS. Cells were analyzed using flow cytometry. NK IFNγ-, CD107a-, and MIP-1β–producing activity is represented as the proportion of NK cells (CD3, CD56, and CD16) positive for the expression of these markers. All antibodies were sourced from BD Biosciences.

### ADNOB

ADNOB was measured by addition of dihydrorhodamine 123 to freshly isolated neutrophils with antibody-opsonized red fluorescent NeutrAvidin-coated microspheres. Serum samples were heat inactivated at 56°C for 30 min and diluted 1:50 before incubation with Vi-coated red fluorescent microspheres. Leukocytes were isolated from EDTA-treated blood from healthy anonymized donors using ammonium-chloride-potassium lysis buffer. 5 × 10^4^ leukocytes were added to each well of antibody-opsonized beads, followed by 10 µl per well of dihydrorhodamine 123 (50 µM), and incubated for 1 h at 37°C. Cells were pelleted and stained as above with APC-Cy7 anti-CD14 (clone MφP9; BD Biosciences), Alexa Fluor 700 anti-CD3 (clone UCHT1; BD Biosciences), and Pacific Blue anti-CD66b (clone G10F5; BioLegend). Cells were washed, fixed with 4% PFA, and analyzed immediately using flow cytometry. The oxidative burst score was calculated from gated neutrophils (CD14^+^, CD3^−^, CD66b^+^).

#### Fc receptor (FcR) binding

Recombinant biotinylated Vi antigen was coupled to streptavidin coated MagPlex beads (Luminex). Heat-inactivated samples were diluted 1:500 in 1× PBS + 0.1% BSA + 0.05% Tween20 and incubated with antigen-coupled beads for 2 h. Beads were washed and incubated with recombinant biotinylated FcRs (activating FcγRs [FcγR2A, FcγR3A, and FcγR3B], the inhibitory FcγR receptor [FcγR2B], and FcαR, which were tetramerized via Streptavidin-PE for 1 h at room temperature. Beads were washed and analyzed on a Flexmap 3D instrument (Luminex). The median fluorescent intensity of 30 beads/region was recorded.

### Statistical analysis

Samples were run in duplicate for all assays. The mean value was calculated and used for statistical analyses. Functional antibody assays were performed twice to demonstrate assay reproducibility, and analyses were performed using the mean result of sample replicates from one assay run.

Univariate analyses comparing baseline and postvaccination measurement within Vi-vaccine groups were conducted using Wilcoxon signed-rank tests. Vaccination responses between Vi-vaccine groups (absolute values measured after vaccination or fold change from baseline), and responses between diagnosed and protected individuals, were conducted using Mann–Whitney *U* tests. P values were adjusted for multiple testing using the Bonferroni method. Adjusted P values <0.05 were considered significant. Statistical analyses were performed using Python statistical software v3.0 and GraphPad Prism v8.0.

Exploratory analyses were undertaken to investigate potential associations between protective signatures and clinical and microbiological outcomes using Spearman correlations. TD participants in Vi-TT and Vi-PS vaccine groups were combined for analysis; P values were not adjusted for multiple testing.

### Multivariate analysis

An Elastic-net/LASSO regularization with PLSDA under a fivefold repeated cross-validation framework was used to determine the combinatorial effects of multiple features together associating to the outcome (Chung et al., 2015; Gunn et al., 2018). Partial least squares (PLS) modeling is a multivariate modeling approach used to transform a high-dimensional immune parameter space into a new low-dimensional space (latent component space). While reducing the dimensionality, PLS extracts a set of orthogonal LVs that together capture the maximum covariance between all the input features and the response (challenge outcome). Each of the LVs was formulated to linearly combine all features based on the feature weights. Derived weights assist with assessing the importance of the individual features’ impact on the predictive model. The Elastic-net/LASSO method, integrating the penalty functions of LASSO and ridge regression, is used to remove irrelevant features to improve the robustness of high-dimensional modeling (Zou and Hastie, 2005). PLSDA models the covariance relationship between the selected feature variables (X) and the outcome variable (Y; Wold et al., 2001). The model is built using a repeated cross-validation framework, to minimize model overfitting driven by outliers, and is coupled with permutation tests to a statistical validation of the classification model. Importantly, the model also calculates the feature weights (variable importance in the projection [VIP] scores), a weighted sum of squares of the PLSDA variable loadings (Galindo-Prieto et al., 2014), to gain enhanced resolution of the degree to which each correlate contributes to the overall model classification.

The mean values of measurements (all samples and assays were run at least in duplicate) were calculated and carried over into the model analyses. The feature matrices were first processed and normalized before multivariate modeling. The features were removed if the missing values were >25% of total subjects or if variance was low (variance ≤ 1; Table 1). Missing values in the matrices were estimated using a *k*–nearest neighbor algorithm, a weighted average of values in *k* closest samples to the missing feature determined by Euclidean distance. The matrices were then normalized by Z-score standardization, which made each feature mean centered and unit variance scaled. To define the minimal correlates that best explained protection, a 5,000-repeated fivefold cross-validation was designed. In each repetition, the dataset was randomly divided into a training and testing dataset, while maintaining approximately the same proportions in each outcome group. A set of the correlates was identified by an Elastic-net/LASSO PLSDA model, and the goodness of fit of the model was measured by accuracy. The frequency of the feature selected among 5,000 repetitive models was calculated (Table S8) and used to rank all features. A step-forward approach was then used to determine the minimal correlates starting from the top feature. Coefficient of variation accuracy was calculated in each step, and the minimal correlates that generated the highest accuracy were selected. Because of the limited sample size and unavailability of an independent dataset, the models trained using cross-validation were not tested in an independent dataset. The customized scripts for multivariate model analysis were performed in Matlab and are available on request (Li et al., 2018).

The correlation network was constructed based on the correlation coefficients between the immunological features measured in this report. Edges between nodes were weighted based on the correlation coefficient (Spearman ρ) between the features represented by the two nodes. Using significant correlation coefficients after correcting for multiple comparisons (Benjamini–Hochberg *q* value <0.01, testing the hypothesis of zero correlation), an adjacency matrix was constructed using soft thresholding to define edge weights. The correlation networks were built using the open-access software Cytoscape v3.7.1.

### Online supplemental material

Fig. S1 demonstrates that protected participants had higher fold increases in Vi IgA responses than diagnosed individuals (baseline to day 28). Fig. S2, Fig. S3, and Fig. S4 show differences in persistent humoral signatures of protection at day 118 and day 208 in Vi-TT and Vi-PS participants combined (Fig. S2), Vi-TT vaccinees (Fig. S3), and Vi-PS vaccinees (Fig. S4).

Table S1 and Table S2 present comparisons in prevaccination and postvaccination Vi-specific humoral responses for Vi-TT (Table S1 A) and Vi-PS vaccinees (Table S1 B) and compare responses between Vi-TT and Vi-PS groups (Table S2). Table S3 and Table S4 present comparisons of VI-specific humoral responses between diagnosed and protected participants using both absolute values (Table S3) and fold change (Table S4) in responses. Table S5 (absolute values) and Table S6 (fold change) compare Vi-specific responses between diagnosed and protected participants vaccinated with Vi-TT and Vi-specific responses between diagnosed and protected Vi-PS vaccinees. Table S7 presents correlations of fold change in Vi IgA titer and Vi IgG titer in diagnosed participants with clinical and laboratory indicators of typhoid fever disease severity. Table S8 presents a list of 34 features measured at 28 days after vaccination, ranked by frequency of selection based on 5,000 repeated fivefold cross-validation models.

## Supplementary Material

Table S1 presents a comparison of prevaccination and postvaccination Vi-specific humoral responses for Vi-TT and Vi-PS vaccinees.

Table S2 compares responses between Vi-TT and Vi-PS groups.

Table S3 presents a comparison of VI-specific humoral responses between diagnosed and protected participants using absolute values.

Table S4 presents a comparison of VI-specific humoral responses between diagnosed and protected participants using fold change in responses.

Table S5 compares Vi-specific responses between diagnosed and protected participants vaccinated with Vi-TT and between diagnosed and protected Vi-PS vaccines, using absolute values.

Table S6 compares Vi-specific responses between diagnosed and protected participants vaccinated with Vi-TT and between diagnosed and protected Vi-PS vaccines, using fold change.

Table S7 presents correlations of fold change in Vi IgA titer and Vi IgG titer in diagnosed participants with clinical and laboratory indicators of typhoid fever disease severity.

Table S8 presents a list of 34 features measured at 28 days after vaccination, ranked by frequency of selection based on 5,000 repeated fivefold cross-validation models.
