# Supplementary material for: Vi-specific serological correlates of protection for typhoid fever
Source: J Exp Med. 2020 Nov 12;218(2):e20201116. doi: 10.1084/jem.20201116 (PMC7668386; doi:10.1084/jem.20201116)
Supplement: Table S1 — presents a comparison of prevaccination and postvaccination Vi-specific humoral responses for Vi-TT and Vi-PS vaccinees. [file JEM_20201116_TableS1.docx]

**Table S1 A. Assessment of vaccine immunogenicity in Vi-TT vaccinees**

Comparisons of baseline and postvaccination (days 28, 118, 208) measurements were performed using Wilcoxon-rank signed tests. Presented P values were adjusted for multiple testing using the Bonferroni correction method (non-significant P values >0.05 were rounded to two decimal places). NA used to denote where testing was unable to be performed due to equivalent responses. Bolded P values are statistically significant.

|  | **Baseline** | | **Day 28** | | | **Day 118** | | | **Day 208** | | |
| --- | --- | --- | --- | --- | --- | --- | --- | --- | --- | --- | --- |
|  | ***n*** | **Median (IQR)** | ***n*** | **Median (IQR)** | **P value** | ***n*** | **Median (IQR)** | **P value** | ***n*** | **Median (IQR)** | **P value** |
| **Antibody quantification** |  |  |  |  |  |  |  |  |  |  |  |
| IgG titer | 37 | 3.7 (3.7-3.7) | 37 | 641.1 (255.5-1224.2) | **< 0.001** | 33 | 300.4 (122.0-601.8) | **< 0.001** | 34 | 336.0 (117.7-723.2) | **< 0.001** |
| IgG1 titer | - | - | 37 | 90.7 (44.1-182.8) | - | - | - | - | - | - | - |
| IgG1 MFI | 37 | 10000 (10000-10000) | 37 | 1036400 (508950-1405100) | **< 0.001** | - | - | - | - | - | - |
| IgG1 MFI (biotinylated) | 37 | 10000 (10000-10000) | 37 | 249000 (123400-644425) | **< 0.001** | 32 | 217650 (78639-452763) | **< 0.001** | 32 | 146972 (44588-264750) | **< 0.001** |
| IgG2 titer | - | - | 37 | 47.2 (21.0-120.3) | - | - | - | - | - | - | - |
| IgG2 MFI | 37 | 5000 (5000-5000) | 37 | 1118750 (799000-3135375) | **< 0.001** | - | - | - | - | - | - |
| IgG2 MFI (biotinylated) | 37 | 4000 (4000-8640) | 37 | 159270 (61120-430960) | **< 0.001** | 31 | 391910 (123870-1523000) | **< 0.001** | 31 | 278940 (53205-579265) | **< 0.001** |
| IgG3 titer | - | - | 37 | 122.0 (61.0-293.6) | - | - | - | - | - | - | - |
| IgG3 MFI (biotinylated) | 37 | 5000 (5000-5000) | 37 | 13138 (5538-20025) | **< 0.001** | 32 | 10771 (5872-15563) | **< 0.001** | 32 | 9906 (5000-16325) | **< 0.001** |
| IgA titer | 33 | 1.6 (1.6-1.6) | 36 | 91.9 (31.8-187.8) | **< 0.001** | 33 | 49.7 (18.7-122.4) | **< 0.001** | 33 | 50.8 (17.8-98.9) | **< 0.001** |
| IgA MFI | 37 | 5000 (5000-5000) | 32 | 904063 (334372-2209400) | **< 0.001** | - | - | - | - | - | - |
| IgA MFI (biotinylated) | 37 | 7800 (5000-21800) | 37 | 1045088 (539250-5020800) | **< 0.001** | 32 | 754300 (280303-1544075) | **< 0.001** | 32 | 585013 (263590-1104903) | **< 0.001** |
| IgA1 MFI | 35 | 20000 (20000-20000) | 35 | 987700 (316725-1803050) | **< 0.001** | - | - | - | - | - | - |
| IgA2 MFI | 36 | 20000 (20000-20000) | 36 | 255200 (68138-1086750) | **< 0.001** | - | - | - | - | - | - |
| IgM titer | 33 | 1.6 (1.6-1.6) | 35 | 62.8 (33.4-228.7) | **< 0.001** | - | - | - | - | - | - |
| **Antibody avidity** |  |  |  |  |  |  |  |  |  |  |  |
| IgG1 AI | - | - | 37 | 71.0 (56.0-95.0) | - | - | - | - | - | - | - |
| IgG1 AI (biotinylated) | - | - | 36 | 28.5 (16.8-46.5) | - | 30 | 33.0 (22.0-53.5) | - | 31 | 30.0 (17.5-48.0) | - |
| IgG2 AI (biotinylated) | - | - | 36 | 45.5 (25.8-63.0) | - | 32 | 37.5 (24.5-48.3) | - | 31 | 43.0 (25.0-56.0) | - |
| IgG3 AI (biotinylated) | - | - | 30 | 36.0 (26.3-46.3) | - | 23 | 38.0 (21.5-47.5) | - | 22 | 35.0 (25.3-48.3) | - |
| IgA AI | - | - | 27 | 20.0 (14.0-33.0) | - | - | - | - | - | - | - |
| IgA AI (biotinylated) | - | - | 37 | 41.0 (25.0-58.0) | - | 31 | 40.0 (26.0-54.0) | - | 32 | 42.0 (26.0-57.0) | - |
| IgA1 AI | - | - | 35 | 18.0 (12.0-30.5) | - | - | - | - | - | - | - |
| IgA2 AI | - | - | 35 | 14.0 (5.0-27.0) | - | - | - | - | - | - | - |
| **Functional properties** |  |  |  |  |  |  |  |  |  |  |  |
| ADCD (biotinylated) | 37 | 0.0 (0.0-0.0) | 37 | 7.4 (2.4-18.0) | **< 0.001** | 31 | 5.8 (2.8-14.0) | **< 0.001** | 31 | 1.4 (0.0-6.2) | **0.001** |
| ADCP (biotinylated) | 37 | 1.1 (0.7-1.4) | 37 | 2.8 (1.9-4.1) | **< 0.001** | 32 | 2.6 (1.6-4.1) | **< 0.001** | 32 | 1.8 (1.1-3.6) | **0.049** |
| ADNP (biotinylated) | 37 | 8.7 (2.9-19.7) | 37 | 101.8 (62.8-162.3) | **< 0.001** | 32 | 63.7 (24.1-132.2) | **< 0.001** | 32 | 30.5 (15.2-73.7) | **0.004** |
| ADNOB (biotinylated) | 37 | 110.0 (10.0-192.5) | 33 | 988.0 (-112.0-1480.0) | **0.043** | - | - | - | - | - | - |
| ADNKA CD107a (biotinylated) | 36 | 6.7 (5.9-7.5) | 35 | 7.2 (6.1-8.2) | 1 | 31 | 7.2 (6.4-8.4) | 1 | 31 | 7.6 (6.3-8.5) | 0.28 |
| ADNKA MIP-1$\beta$ (biotinylated) | 36 | 5.4 (4.3-6.9) | 35 | 12.5 (8.4-15.9) | **< 0.001** | 31 | 10.6 (6.8-14.4) | **< 0.001** | 31 | 9.8 (7.1-12.6) | **< 0.001** |
| ADNKA IFN$\gamma$ (biotinylated) | 36 | 7.1 (4.4-9.5) | 35 | 6.8 (5.1-10.8) | 1 | 31 | 8.1 (6.0-10.8) | 0.13 | 31 | 8.8 (5.5-13.2) | 0.07 |
| **Fc receptor binding** |  |  |  |  |  |  |  |  |  |  |  |
| Fc$\alpha$R (biotinylated) | 37 | 1378 (1138-2114) | 37 | 16417 (9032-30565) | **< 0.001** | - | - | - | - | - | - |
| Fc$\gamma$R2A binding (biotinylated) | 37 | 5143 (2905-7369) | 37 | 29324 (19931-36939) | **< 0.001** | - | - | - | - | - | - |
| Fc$\gamma$R2B binding (biotinylated) | 37 | 2101 (1464-2706) | 37 | 6213 (3588-9932) | **< 0.001** | - | - | - | - | - | - |
| Fc$\gamma$R3A binding (biotinylated) | 37 | 1491 (1192-2417) | 37 | 13620 (8886-18970) | **< 0.001** | - | - | - | - | - | - |
| Fc$\gamma$R3B binding (biotinylated) | 37 | 1188 (854-1665) | 37 | 9452 (4847-14685) | **< 0.001** | - | - | - | - | - | - |

**Table S1 B. Assessment of vaccine immunogenicity in Vi-PS vaccinees**

Comparisons of baseline and postvaccination (days 28, 118, 208) measurements were performed using Wilcoxon-rank signed tests. Presented P values were adjusted for multiple testing using the Bonferroni correction method (non-significant P values >0.05 were rounded to two decimal places). NA used to denote where testing was unable to be performed due to equivalent responses.

|  | **Baseline** | | **Day 28** | | | **Day 118** | | | **Day 208** | | |
| --- | --- | --- | --- | --- | --- | --- | --- | --- | --- | --- | --- |
|  | ***n*** | **Median (IQR)** | ***n*** | **Median (IQR)** | **P value** | ***n*** | **Median (IQR)** | **P value** | ***n*** | **Median (IQR)** | **P value** |
| **Antibody quantification** |  |  |  |  |  |  |  |  |  |  |  |
| IgG titer | 35 | 3.7 (3.7-8.6) | 35 | 123.4 (67.0-359.4) | **< 0.001** | 29 | 115.3 (68.4-384.8) | **< 0.001** | 29 | 120.3 (74.0-243.3) | **< 0.001** |
| IgG1 titer | - | - | 35 | 13.5 (6.1-27.2) | - | - | - | - | - | - | - |
| IgG1 MFI | 35 | 10000 (10000-10000) | 35 | 134700 (27250-396338) | **< 0.001** | - | - | - | - | - | - |
| IgG1 MFI (biotinylated) | 35 | 10000 (10000-10000) | 35 | 38150 (12988-87813) | **< 0.001** | 30 | 34647 (12755-91705) | **< 0.001** | 27 | 26719 (10463-91350) | **0.007** |
| IgG2 titer | - | - | 35 | 12.3 (6.6-39.3) | - | - | - | - | - | - | - |
| IgG2 MFI | 35 | 50000 (50000-50000) | 35 | 276875 (50000-1195063) | **< 0.001** | - | - | - | - | - | - |
| IgG2 MFI (biotinylated) | 35 | 4630 (4000-21405) | 35 | 62480 (17575-225435) | **< 0.001** | 30 | 113870 (39348-430245) | **< 0.001** | 27 | 136400 (34160-407165) | **< 0.001** |
| IgG3 titer | - | - | 35 | 22.4 (15.1-44.3) | - | - | - | - | - | - | - |
| IgG3 MFI (biotinylated) | 35 | 5000 (5000-5000) | 35 | 5000 (5000-8606) | **0.043** | 30 | 5000 (5000-5000) | 0.39 | 27 | 5000 (5000-5000) | 1 |
| IgA titer | 34 | 1.6 (1.6-1.6) | 34 | 30.2 (14.3-63.8) | **< 0.001** | 30 | 29.2 (12.4-60.2) | **< 0.001** | 28 | 28.3 (14.0-69.6) | **< 0.001** |
| IgA MFI | 35 | 5000 (5000-5000) | 34 | 275638 (139147-582784) | **< 0.001** | - | - | - | - | - | - |
| IgA MFI (biotinylated) | 35 | 8975 (5000-18119) | 34 | 471419 (185487-666156) | **< 0.001** | 30 | 396225 (199363-647706) | **< 0.001** | 27 | 366225 (178332-813856) | **< 0.001** |
| IgA1 MFI | 34 | 20000 (20000-20000) | 34 | 308225 (115663-522913) | **< 0.001** | - | - | - | - | - | - |
| IgA2 MFI | 34 | 20000 (20000-20000) | 33 | 160750 (20000-492800) | **< 0.001** | - | - | - | - | - | - |
| IgM titer | 34 | 1.6 (1.6-1.6) | 34 | 37.4 (16.2-71.8) | **< 0.001** | - | - | - | - | - | - |
| **Antibody avidity** |  |  |  |  |  |  |  |  |  |  |  |
| IgG1 AI | - | - | 30 | 29.0 (18.5-42.8) | - | - | - | - | - | - | - |
| IgG1 AI (biotinylated) | - | - | 27 | 8.0 (1.0-34.0) | - | 24 | 12.0 (3.0-21.8) | - | 21 | 12.0 (3.0-24.0) | - |
| IgG2 AI (biotinylated) | - | - | 34 | 26.0 (10.8-48.0) | - | 30 | 24.0 (11.0-52.5) | **-** | 26 | 26.0 (8.3-46.3) | **-** |
| IgG3 AI (biotinylated) | - | - | 13 | 12.0 (4.0-19.0) | - | 6 | 6.0 (5.3-32.3) | - | 4 | 20.0 (3.3-37.5) | - |
| IgA AI | - | - | 24 | 16.5 (9.0-26.3) | - | - | - | - | - | - | - |
| IgA AI (biotinylated) | - | - | 34 | 37.5 (21.5-47.0) | - | 30 | 34.0 (18.5-42.8) | **-** | 27 | 36.0 (22.5-44.0) | **-** |
| IgA1 AI | - | - | 33 | 14.0 (7.0-20.0) | - | - | - | - | - | - | - |
| IgA2 AI | - | - | 24 | 12.5 (6.5-19.3) | - | - | - | - | - | - | - |
| **Functional properties** |  |  |  |  |  |  |  |  |  |  |  |
| ADCD (biotinylated) | 35 | 0.0 (0.0-0.0) | 35 | 3.3 (0.7-6.7) | **< 0.001** | 28 | 1.8 (0.3-7.2) | **< 0.001** | 27 | 0.4 (0.0-2.2) | **0.014** |
| ADCP (biotinylated) | 35 | 1.0 (0.5-1.3) | 35 | 1.5 (1.0-2.2) | **< 0.001** | 30 | 1.3 (0.8-2.6) | 0.2 | 27 | 1.4 (0.8-2.4) | 0.17 |
| ADNP (biotinylated) | 35 | 7.3 (2.5-11.0) | 35 | 34.2 (11.1-69.2) | **< 0.001** | 30 | 15.5 (7.3-43.3) | **0.036** | 27 | 15.7 (7.9-58.8) | **0.037** |
| ADNOB (biotinylated) | 34 | 129.5 (23.6-249.5) | 32 | -141.0 (-340.8-1093.0) | 1 | - | - | - | - | - | - |
| ADNKA CD107a (biotinylated) | 33 | 6.2 (5.4-7.6) | 34 | 7.2 (5.6-8.2) | 0.69 | 28 | 6.9 (5.7-7.8) | 1 | 25 | 7.1 (5.8-7.8) | 0.42 |
| ADNKA MIP-1$\beta$ (biotinylated) | 33 | 5.5 (5.2-6.7) | 34 | 9.0 (6.4-13.4) | **0.004** | 28 | 9.2 (5.8-11.3) | **0.002** | 25 | 8.7 (6.8-10.5) | **0.009** |
| ADNKA IFN$\gamma$ (biotinylated) | 33 | 6.2 (4.4-9.3) | 34 | 6.9 (5.8-9.4) | 0.13 | 28 | 7.9 (5.3-10.3) | **0.011** | 25 | 8.1 (5.9-11.8) | **0.005** |
| **Fc receptor binding** |  |  |  |  |  |  |  |  |  |  |  |
| Fc$\alpha$R (biotinylated) | 35 | 1393 (1101-2220) | 35 | 8257 (4858-12609) | **< 0.001** | - | - | - | - | - | - |
| Fc$\gamma$R2A binding (biotinylated) | 35 | 4897 (3686-6043) | 35 | 11720 (6964-21829) | **< 0.001** | - | - | - | - | - | - |
| Fc$\gamma$R2B binding (biotinylated) | 35 | 1887 (1407-2498) | 35 | 2594 (1845-3897) | 0.23 | - | - | - | - | - | - |
| Fc$\gamma$R3A binding (biotinylated) | 35 | 1454 (1177-2464) | 35 | 4422 (1985-6417) | **< 0.001** | - | - | - | - | - | - |
| Fc$\gamma$R3B binding (biotinylated) | 35 | 1081 (901-1515) | 35 | 2619 (1344-3897) | **< 0.001** | - | - | - | - | - | - |
