# Supplementary material for: Vi-specific serological correlates of protection for typhoid fever
Source: J Exp Med. 2020 Nov 12;218(2):e20201116. doi: 10.1084/jem.20201116 (PMC7668386; doi:10.1084/jem.20201116)
Supplement: Table S2 — compares responses between Vi-TT and Vi-PS groups. [file JEM_20201116_TableS2.docx]

**Table S2. Comparison of fold-change in Vi-specific measures between Vi-TT and Vi-PS groups**

Fold change in Vi-specific measures from baseline were calculated for each of the postvaccination time points. Comparisons of fold change between vaccine groups were performed using Mann Whitney *U* tests. Presented P values were adjusted for multiple testing using the Bonferroni correction method (nonsignificant P values >0.05 were rounded to two decimal places). Bolded P values are statistically significant.

|  | **Day 28** | | | | | **Day 118** | | | | | **Day 208** | | | | |
| --- | --- | --- | --- | --- | --- | --- | --- | --- | --- | --- | --- | --- | --- | --- | --- |
|  | **Vi-TT** | | **Vi-PS** | | **P value** | **Vi-TT** | | **Vi-PS** | | **P value** | **Vi-TT** | | **Vi-PS** | | **P value** |
|  | ***n*** | **Median**  **(IQR)** | ***n*** | **Median (IQR)** |  | ***n*** | **Median**  **(IQR)** | ***n*** | **Median (IQR)** |  | ***n*** | **Median**  **(IQR)** | ***n*** | **Median (IQR)** |  |
| **Antibody quantification** |  |  |  |  |  |  |  |  |  |  |  |  |  |  |  |
| IgG titer | 37 | 107.0 (67.3-182.7) | 35 | 26.2 (10.8-43.5) | **< 0.001** | 33 | 68.6 (27.0-118.3) | 29 | 24.5 (13.6-63.4) | 0.45 | 34 | 46.1 (23.4-101.8) | 29 | 26.1 (14.6-57.2) | 1 |
| IgG1 titer | - | - | - | - | - | - | - | - | - | - | - | - | - | - | - |
| IgG1 MFI | 37 | 72.4 (48.4-136.4) | 35 | 11.3 (2.7-29.1) | **< 0.001** | - | - | - | - | - | - | - | - | - | - |
| IgG1 MFI (biotinylated) | 37 | 19.7 (5.0-39.4) | 35 | 3.3 (1.2-8.1) | **0.011** | 32 | 12.2 (5.9-25.3) | 30 | 2.3 (1.2-5.7) | **0.017** | 32 | 9.3 (3.0-19.0) | 27 | 2.2 (1.0-5.2) | 0.05 |
| IgG2 titer | - | - | - | - | - | - | - | - | - | - | - | - | - | - | - |
| IgG2 MFI | 37 | 21.9 (16.0-52.4) | 35 | 5.5 (1.0-23.9) | **0.019** | - | - | - | - | - | - | - | - | - | - |
| IgG2 MFI (biotinylated) | 37 | 22.0 (9.8-62.1) | 35 | 8.2 (2.3-17.1) | 0.06 | 31 | 51.8 (13.1-105.4) | 30 | 15.2 (4.4-36.7) | 0.21 | 31 | 27.9 (8.2-73.2) | 27 | 13.1 (2.5-34.7) | 1 |
| IgG3 titer | - | - | - | - | - | - | - | - | - | - | - | - | - | - | - |
| IgG3 MFI (biotinylated) | 37 | 2.6 (1.1-4.0) | 35 | 1.0 (1.0-1.7) | **0.001** | 32 | 2.2 (1.2-3.1) | 30 | 1.0 (1.0-1.0) | **< 0.001** | 32 | 2.0 (1.0-3.3) | 27 | 1.0 (1.0-1.0) | **< 0.001** |
| IgA titer | 33 | 44.3 (17.0-103.2) | 34 | 18.7 (4.1-38.0) | 0.21 | 31 | 26.0 (7.3-53.3) | 29 | 17.0 (3.1-33.0) | 1 | 31 | 24.1 (6.3-51.8) | 28 | 18.1 (3.6-39.5) | 1 |
| IgA MFI | 32 | 134.5 (47.2-343.0) | 34 | 54.8 (13.8-116.6) | 0.10 | - | - | - | - | - | - | - | - | - | - |
| IgA MFI (biotinylated) | 37 | 127.8 (37.1-568.6) | 34 | 41.9 (13.6-86.3) | 0.09 | 32 | 111.8 (32.2-184.9) | 30 | 39.4 (10.5-66.4) | 0.69 | 32 | 62.1 (12.2-152.6) | 27 | 43.0 (10.5-73.7) | 1 |
| IgA1 MFI | 35 | 44.3 (13.5-82.1) | 34 | 15.4 (5.2-26.1) | **0.037** | - | - | - | - | - | - | - | - | - | - |
| IgA2 MFI | 36 | 12.8 (3.4-54.3) | 33 | 8.0 (1.0-24.6) | 1 | - | - | - | - | - | - | - | - | - | - |
| IgM titer | 32 | 24.1 (11.9-92.9) | 34 | 9.9 (5.4-22.1) | **0.041** | - | - | - | - | - | - | - | - | - | - |
| **Functional properties** |  |  |  |  |  |  |  |  |  |  |  |  |  |  |  |
| ADCD (biotinylated) | 8 | 25.6 (7.5-45.9) | 4 | 4.3 (3.3-9.4) | 1 | 7 | 11.7 (4.8-21.4) | 4 | 2.2 (1.4-7.1) | 1 | 7 | 4.3 (1.8-9.8) | 3 | 2.7 (2.0-7.3) | 1 |
| ADCP (biotinylated) | 37 | 2.3 (1.5-4.7) | 35 | 1.7 (1.3-2.7) | 1 | 32 | 2.0 (1.6-3.6) | 30 | 1.7 (0.8-3.0) | 1 | 32 | 1.5 (0.9-3.0) | 27 | 2.2 (0.9-4.6) | 1 |
| ADNP (biotinylated) | 35 | 10.6 (3.5-25.5) | 33 | 4.3 (1.8-10.6) | 1 | 30 | 6.6 (2.8-22.4) | 28 | 3.0 (0.9-12.1) | 0.91 | 30 | 3.2 (1.0-9.2) | 25 | 4.0 (0.9-8.5) | 1 |
| ADNOB (biotinylated) | 33 | -0.5 (-9.0-12.7) | 31 | -0.7 (-3.4-6.5) | 1 | - | - | - | - | - | - | - | - | - | - |
| ADNKA CD107a (biotinylated) | 34 | 1.1 (0.9-1.3) | 33 | 1.1 (1.0-1.3) | 1 | 31 | 1.1 (0.9-1.3) | 28 | 1.0 (0.9-1.3) | 1 | 31 | 1.2 (1.0-1.4) | 25 | 1.1 (1.0-1.3) | 1 |
| ADNKA MIP-1$\beta$ (biotinylated) | 34 | 2.2 (1.6-2.8) | 33 | 1.4 (1.0-2.4) | 0.62 | 31 | 2.0 (1.5-2.8) | 28 | 1.4 (1.2-1.8) | 0.52 | 31 | 1.7 (1.1-2.6) | 25 | 1.5 (1.1-1.7) | 1 |
| ADNKA IFN$\gamma$ (biotinylated) | 34 | 1.2 (0.8-1.4) | 33 | 1.3 (1.0-1.5) | 1 | 31 | 1.3 (1.0-1.6) | 28 | 1.3 (1.0-1.5) | 1 | 31 | 1.2 (0.9-1.7) | 25 | 1.3 (1.1-1.6) | 1 |
| **Fc Receptor binding** |  |  |  |  |  |  |  |  |  |  |  |  |  |  |  |
| Fc$\alpha$R binding (biotinylated) | 37 | 11.0 (6.2-17.0) | 35 | 5.0 (2.7-10.6) | 0.06 | - | - | - | - | - | - | - | - | - | - |
| Fc$\gamma$R2A binding (biotinylated) | 37 | 5.0 (4.1-7.3) | 35 | 2.6 (1.5-3.9) | **< 0.001** | - | - | - | - | - | - | - | - | - | - |
| Fc$\gamma$R2B binding (biotinylated) | 37 | 3.0 (1.9-5.7) | 35 | 1.3 (0.9-1.7) | **< 0.001** | - | - | - | - | - | - | - | - | - | - |
| Fc$\gamma$R3A binding (biotinylated) | 37 | 7.8 (5.2-9.6) | 35 | 2.2 (1.2-4.4) | **< 0.001** | - | - | - | - | - | - | - | - | - | - |
| Fc$\gamma$R3B binding (biotinylated) | 37 | 7.1 (3.3-11.4) | 35 | 1.9 (1.1-3.0) | **< 0.001** | - | - | - | - | - | - | - | - | - | - |
