# Supplementary material for: Vi-specific serological correlates of protection for typhoid fever
Source: J Exp Med. 2020 Nov 12;218(2):e20201116. doi: 10.1084/jem.20201116 (PMC7668386; doi:10.1084/jem.20201116)
Supplement: Table S3 — presents a comparison of VI-specific humoral responses between diagnosed and protected participants using absolute values. [file JEM_20201116_TableS3.docx]

**Table S3 A. Comparison of Vi-specific measures between diagnosed and protected individuals without P value adjustment**

Comparisons of Vi-specific measures between diagnosed and protected individuals were performed using Mann Whitney *U* tests. Presented P values are unadjusted for multiple testing (non-significant P values >0.05 were rounded to two decimal places). Bolded P values are statistically significant.

|  | **Baseline** | | | | | **Day 28** | | | | | **Day 118** | | | | | **Day 208** | | | | |
| --- | --- | --- | --- | --- | --- | --- | --- | --- | --- | --- | --- | --- | --- | --- | --- | --- | --- | --- | --- | --- |
|  | **Diagnosed** | | **Protected** | | **P value** | **Diagnosed** | | **Protected** | | **P value** | **Diagnosed** | | **Protected** | | **P value** | **Diagnosed** | | **Protected** | | **P value** |
|  | **n** | **Median (IQR)** | **n** | **Median (IQR)** |  | **n** | **Median (IQR)** | **n** | **Median (IQR)** |  | **n** | **Median (IQR)** | **n** | **Median (IQR)** |  | **n** | **Median (IQR)** | **n** | **Median (IQR)** |  |
| **Antibody quantification** |  |  |  |  |  |  |  |  |  |  |  |  |  |  |  |  |  |  |  |  |
| IgG titer | 26 | 3.7 (3.7-8.0) | 46 | 3.7 (3.7-6.6) | 0.70 | 26 | 160.9 (83.2-461.3) | 46 | 434.5 (140.3-853.5) | 0.05 | 21 | 110.3 (69.9-234.5) | 41 | 291.3 (104.6-571.9) | 0.06 | 22 | 118.1 (57.7-233.0) | 41 | 250.4 (96.5-662.9) | **0.017** |
| IgG1 titer | - | - | - | - | - | 26 | 21.0 (10.0-84.5) | 46 | 44.4 (22.5-113.3) | 0.22 | - | - | - | - | - | - | - | - | - | - |
| IgG1 MFI | 26 | 10000 (10000-10000 | 46 | 10000 (10000-10000) | 0.11 | 26 | 348075 (50275-1221119) | 46 | 568000 (132019-1139656) | 0.53 | - | - | - | - | - | - | - | - | - | - |
| IgG1 MFI (biotinylated) | 26 | 10000 (10000-21194) | 46 | 10000 (10000-10000) | **0.012** | 26 | 123788 (32294-313844) | 46 | 116325 (19889-382194) | 0.86 | 21 | 102975 (31981-475450) | 41 | 82575 (14313-260150) | 0.52 | 21 | 49963 (29850-293025) | 38 | 89488 (12914-208188) | 0.54 |
| IgG2 titer | - | - | - | - | - | 26 | 16.1 (6.7-36.0) | 46 | 37.5 (15.3-93.4) | **0.020** | - | - | - | - | - | - | - | - | - | - |
| IgG2 MFI | 26 | 50000 (50000-50000) | 46 | 50000 (50000-50000) | 0.53 | 26 | 523000 (73281-1063563) | 46 | 1039688 (240688-2103625) | 0.09 | - | - | - | - | - | - | - | - | - | - |
| IgG2 MFI (biotinylated) | 26 | 4000 (4000-12338) | 46 | 4000 (4000-11073) | 0.68 | 26 | 91870 (37930-245358) | 46 | 110955 (37908-403940) | 0.52 | 21 | 244780 (49590-492970) | 40 | 253185 (78805-703980) | 0.62 | 21 | 207570 (33700-417950) | 37 | 264120 (48530-711070) | 0.45 |
| IgG3 titer | - | - | - | - | - | 26 | 30.9 (17.0-98.2) | 46 | 67.3 (31.0-157.7) | 0.11 | - | - | - | - | - | - | - | - | - | - |
| IgG3 MFI (biotinylated) | 26 | 5000 (5000-5000) | 46 | 5000 (5000-5000) | 1 | 26 | 5063 (5000-10791) | 46 | 8050 (5000-14844) | 0.30 | 21 | 5000 (5000-12938) | 41 | 6113 (5000-12150) | 0.45 | 21 | 5000 (5000-8763) | 38 | 5000 (5000-12244) | 0.32 |
| IgA titer | 23 | 1.6 (1.6-1.6) | 44 | 1.6 (1.6-1.6) | 0.48 | 26 | 28.7 (8.6-120.6) | 44 | 63.5 (30.7-160.4) | **0.020** | 22 | 14.2 (4.7-61.6) | 41 | 42.8 (25.3-111.1) | **0.016** | 21 | 17.4 (5.8-73.8) | 40 | 49.1 (22.0-99.2) | **0.018** |
| IgA MFI | 26 | 5000 (5000-5038) | 46 | 5000 (5000-5000) | 0.20 | 25 | 194825 (57350-701475) | 41 | 568213 (288500-1542800) | **0.004** | - | - | - | - | - | - | - | - | - | - |
| IgA MFI (biotinylated) | 26 | 11050 (5066-29622) | 46 | 7313 (5000-16831) | 0.13 | 26 | 440275 (115850-817726) | 45 | 724150 (496300-3344900) | **0.035** | 21 | 335912 (62925-713650) | 41 | 592375 (364562-1134788) | 0.06 | 21 | 316662 (54838-639375) | 38 | 668238 (321781-1097975) | **0.021** |
| IgA1 MFI | 23 | 20000 (20000-20000) | 46 | 20000 (20000-20000) | 0.16 | 23 | 215450 (97950-769975) | 46 | 630650 (276500-1468263) | **0.030** | - | - | - | - | - | - | - | - | - | - |
| IgA2 MFI | 24 | 20000 (20000-20000) | 46 | 20000 (20000-20000) | 1 | 24 | 79850 (21200-418275) | 45 | 310400 (80850-1195450) | **0.032** | - | - | - | - | - | - | - | - | - | - |
| IgM titer | 23 | 1.6 (1.6-7.4) | 44 | 1.6 (1.6-4.8) | 0.37 | 26 | 47.8 (23.6-135.9) | 43 | 45.7 (26.5-130.9) | 0.93 | - | - | - | - | - | - | - | - | - | - |
| **Antibody avidity** |  |  |  |  |  |  |  |  |  |  |  |  |  |  |  |  |  |  |  |  |
| IgG1 AI | - | - | - | - | - | 23 | 57.0 (28.5-72.5) | 44 | 53.5 (33.3-82.3) | 0.68 | - | - | - | - | - | - | - | - | - | - |
| IgG1 AI (biotinylated) | - | - | - | - | - | 24 | 10.0 (2.8-36.3) | 39 | 28.0 (13.5-47.0) | 0.07 | 20 | 15.5 (3.0-42.5) | 34 | 24.5 (14.0-41.8) | 0.34 | 19 | 18.0 (5.0-48.5) | 33 | 23.0 (12.0-37.0) | 0.63 |
| IgG2 AI (biotinylated) | - | - | - | - | - | 26 | 26.0 (11.3-53.5) | 44 | 40.0 (22.0-56.8) | 0.10 | 21 | 25.0 (11.0-44.0) | 41 | 33.0 (13.0-51.0) | 0.33 | 21 | 26.0 (7.0-49.0) | 36 | 41.0 (25.0-52.3) | 0.15 |
| IgG3 AI (biotinylated) | - | - | - | - | - | 13 | 21.0 (12.0-40.0) | 30 | 29.5 (23.3-46.3) | 0.17 | 9 | 25.0 (16.0-44.0) | 20 | 37.0 (18.8-45.5) | 0.92 | 8 | 31.0 (15.0-45.3) | 18 | 35.0 (26.3-41.8) | 0.74 |
| IgA AI | - | - | - | - | - | 21 | 14.0 (12.0-24.0) | 30 | 21.0 (14.5-30.8) | 0.16 | - | - | - | - | - | - | - | - | - | - |
| IgA AI (biotinylated) | - | - | - | - | - | 26 | 29.5 (23.0-45.5) | 45 | 41.0 (28.0-54.0) | 0.08 | 21 | 34.0 (20.0-48.0) | 40 | 36.0 (26.0-48.8) | 0.45 | 21 | 34.0 (22.0-45.0) | 38 | 40.5 (26.5-54.5) | 0.25 |
| IgA1 AI | - | - | - | - | - | 22 | 15.0 (7.3-17.8) | 46 | 17.5 (11.0-23.0) | 0.17 | - | - | - | - | - | - | - | - | - | - |
| IgA2 AI | - | - | - | - | - | 18 | 7.5 (5.3-18.8) | 41 | 15.0 (5.0-27.0) | 0.14 | - | - | - | - | - | - | - | - | - | - |
| **Functional properties** |  |  |  |  |  |  |  |  |  |  |  |  |  |  |  |  |  |  |  |  |
| ADCD (biotinylated) | 26 | 0.0 (0.0-0.2) | 46 | 0.0 (0.0-0.0) | 0.08 | 26 | 3.3 (1.0-12.9) | 46 | 5.6 (1.6-15.0) | 0.34 | 20 | 1.7 (0.3-9.0) | 39 | 4.9 (1.8-12.3) | 0.09 | 20 | 0.6 (0.0-3.4) | 38 | 1.4 (0.0-3.9) | 0.49 |
| ADCP (biotinylated) | 26 | 0.9 (0.4-1.4) | 46 | 1.0 (0.8-1.4) | 0.45 | 26 | 1.8 (0.9-3.8) | 46 | 2.3 (1.6-3.8) | 0.28 | 21 | 1.5 (0.8-3.6) | 41 | 1.9 (1.3-3.8) | 0.29 | 21 | 1.2 (0.9-3.4) | 38 | 1.7 (1.2-2.8) | 0.38 |
| ADNP (biotinylated) | 26 | 8.0 (3.9-18.6) | 46 | 7.6 (2.5-13.3) | 0.57 | 26 | 45.4 (19.8-93.9) | 46 | 91.2 (34.1-146.7) | 0.09 | 21 | 19.9 (13.6-70.8) | 41 | 40.6 (14.4-109.1) | 0.40 | 21 | 15.7 (10.5-44.5) | 38 | 31.6 (13.5-76.9) | 0.13 |
| ADNOB (biotinylated) | 25 | 129.0 (18.5-249.5) | 46 | 113.5 (12.1-192.9) | 0.88 | 24 | -111.3 (-210.1-1051.5) | 41 | 451.5 (-336.5-1480.0) | 0.53 | - | - | - | - | - | - | - | - | - | - |
| ADNKA CD107a (biotinylated) | 26 | 6.4 (5.7-7.2) | 43 | 6.6 (5.4-7.7) | 0.70 | 24 | 6.9 (5.7-8.1) | 45 | 7.4 (6.0-8.6) | 0.34 | 21 | 7.0 (5.5-7.5) | 38 | 7.0 (6.3-8.5) | 0.29 | 21 | 7.2 (5.8-8.2) | 35 | 7.3 (6.0-8.3) | 0.80 |
| ADNKA MIP-1$\beta$ (biotinylated) | 26 | 5.6 (5.2-6.7) | 43 | 5.5 (4.1-6.9) | 0.37 | 24 | 9.3 (7.8-15.5) | 45 | 10.2 (6.9-13.9) | 0.92 | 21 | 8.8 (6.4-11.8) | 38 | 9.9 (5.2-13.8) | 0.82 | 21 | 9.1 (6.9-10.5) | 35 | 9.3 (6.7-12.3) | 0.91 |
| ADNKA IFN$\gamma$ (biotinylated) | 26 | 8.0 (5.9-11.6) | 43 | 6.0 (4.2-7.9) | **0.027** | 24 | 9.1 (6.5-12.9) | 45 | 6.2 (5.1-9.1) | **0.009** | 21 | 8.7 (6.6-11.6) | 38 | 7.5 (5.2-10.1) | 0.20 | 21 | 11.4 (7.3-12.9) | 35 | 6.8 (4.9-11.9) | **0.036** |
| **Fc receptor binding** |  |  |  |  |  |  |  |  |  |  |  |  |  |  |  |  |  |  |  |  |
| Fc$\alpha$R (biotinylated) | 26 | 1431 (1206-2261) | 46 | 1353 (1096-1981) | 0.29 | 26 | 7663 (4549-16095) | 46 | 13798 (8280-22721) | **0.047** | - | - | - | - | - | - | - | - | - | - |
| Fc$\gamma$R2A binding (biotinylated) | 26 | 5488 (3721-7303) | 46 | 4711 (2791-6482) | 0.12 | 26 | 15345 (10286-28030) | 46 | 22161 (11098-34907) | 0.22 | - | - | - | - | - | - | - | - | - | - |
| Fc$\gamma$R2B binding (biotinylated) | 26 | 2369 (1780-2801) | 46 | 1844 (1356-2589) | 0.06 | 26 | 3751 (2555-6246) | 46 | 3748 (2147-7322) | 0.81 | - | - | - | - | - | - | - | - | - | - |
| Fc$\gamma$R3A binding (biotinylated) | 26 | 1965 (1334-2649) | 46 | 1404 (1133-2332) | **0.036** | 26 | 6737 (3182-16119) | 46 | 7647 (2960-16099) | 0.92 | - | - | - | - | - | - | - | - | - | - |
| Fc$\gamma$R3B binding (biotinylated) | 26 | 1351 (1059-1971) | 46 | 1005 (850-1488) | **0.017** | 26 | 3929 (2067-9217) | 46 | 4740 (1840-12322) | 0.71 | - | - | - | - | - | - | - | - | - | - |

**Table S3 B. Comparison of Vi-specific measures between diagnosed and protected individuals**

Comparisons of Vi-specific measures between diagnosed and protected individuals were performed using Mann Whitney U tests. Presented P values were adjusted for multiple testing using the Bonferroni correction method (non-significant P values >0.05 were rounded to two decimal places).

|  | **Baseline** | | | | | **Day 28** | | | | | **Day 118** | | | | | **Day 208** | | | | |
| --- | --- | --- | --- | --- | --- | --- | --- | --- | --- | --- | --- | --- | --- | --- | --- | --- | --- | --- | --- | --- |
|  | **Diagnosed** | | **Protected** | | **P value** | **Diagnosed** | | **Protected** | | **P value** | **Diagnosed** | | **Protected** | | **P value** | **Diagnosed** | | **Protected** | | **P value** |
|  | **n** | **Median (IQR)** | **n** | **Median (IQR)** |  | **n** | **Median (IQR)** | **n** | **Median (IQR)** |  | **n** | **Median (IQR)** | **n** | **Median (IQR)** |  | **n** | **Median (IQR)** | **n** | **Median (IQR)** |  |
| **Antibody quantification** |  |  |  |  |  |  |  |  |  |  |  |  |  |  |  |  |  |  |  |  |
| IgG titer | 26 | 3.7 (3.7-8.0) | 46 | 3.7 (3.7-6.6) | 1 | 26 | 160.9 (83.2-461.3) | 46 | 434.5 (140.3-853.5) | 1 | 21 | 110.3 (69.9-234.5) | 41 | 291.3 (104.6-571.9) | 0.88 | 22 | 118.1 (57.7-233.0) | 41 | 250.4 (96.5-662.9) | 0.27 |
| IgG1 titer | - | - | - | - | - | 26 | 21.0 (10.0-84.5) | 46 | 44.4 (22.5-113.3) | 1 | - | - | - | - | - | - | - | - | - | - |
| IgG1 MFI | 26 | 10000 (10000-10000 | 46 | 10000 (10000-10000) | 1 | 26 | 348075 (50275-1221119) | 46 | 568000 (132019-1139656) | 1 | - | - | - | - | - | - | - | - | - | - |
| IgG1 MFI (biotinylated) | 26 | 10000 (10000-21194) | 46 | 10000 (10000-10000) | 0.37 | 26 | 123788 (32294-313844) | 46 | 116325 (19889-382194) | 1 | 21 | 102975 (31981-475450) | 41 | 82575 (14313-260150) | 1 | 21 | 49963 (29850-293025) | 38 | 89488 (12914-208188) | 1 |
| IgG2 titer | - | - | - | - | - | 26 | 16.1 (6.7-36.0) | 46 | 37.5 (15.3-93.4) | 0.72 | - | - | - | - | - | - | - | - | - | - |
| IgG2 MFI | 26 | 50000 (50000-50000) | 46 | 50000 (50000-50000) | 1 | 26 | 523000 (73281-1063563) | 46 | 1039688 (240688-2103625) | 1 | - | - | - | - | - | - | - | - | - | - |
| IgG2 MFI (biotinylated) | 26 | 4000 (4000-12338) | 46 | 4000 (4000-11073) | 1 | 26 | 91870 (37930-245358) | 46 | 110955 (37908-403940) | 1 | 21 | 244780 (49590-492970) | 40 | 253185 (78805-703980) | 1 | 21 | 207570 (33700-417950) | 37 | 264120 (48530-711070) | 1 |
| IgG3 titer | - | - | - | - | - | 26 | 30.9 (17.0-98.2) | 46 | 67.3 (31.0-157.7) | 1 | - | - | - | - | - | - | - | - | - | - |
| IgG3 MFI (biotinylated) | 26 | 5000 (5000-5000) | 46 | 5000 (5000-5000) | 1 | 26 | 5063 (5000-10791) | 46 | 8050 (5000-14844) | 1 | 21 | 5000 (5000-12938) | 41 | 6113 (5000-12150) | 1 | 21 | 5000 (5000-8763) | 38 | 5000 (5000-12244) | 1 |
| IgA titer | 23 | 1.6 (1.6-1.6) | 44 | 1.6 (1.6-1.6) | 1 | 26 | 28.7 (8.6-120.6) | 44 | 63.5 (30.7-160.4) | 0.73 | 22 | 14.2 (4.7-61.6) | 41 | 42.8 (25.3-111.1) | 0.25 | 21 | 17.4 (5.8-73.8) | 40 | 49.1 (22.0-99.2) | 0.28 |
| IgA MFI | 26 | 5000 (5000-5038) | 46 | 5000 (5000-5000) | 1 | 25 | 194825 (57350-701475) | 41 | 568213 (288500-1542800) | 0.14 | - | - | - | - | - | - | - | - | - | - |
| IgA MFI (biotinylated) | 26 | 11050 (5066-29622) | 46 | 7313 (5000-16831) | 1 | 26 | 440275 (115850-817726) | 45 | 724150 (496300-3344900) | 1 | 21 | 335912 (62925-713650) | 41 | 592375 (364562-1134788) | 0.91 | 21 | 316662 (54838-639375) | 38 | 668238 (321781-1097975) | 0.34 |
| IgA1 MFI | 23 | 20000 (20000-20000) | 46 | 20000 (20000-20000) | 1 | 23 | 215450 (97950-769975) | 46 | 630650 (276500-1468263) | 1 | - | - | - | - | - | - | - | - | - | - |
| IgA2 MFI | 24 | 20000 (20000-20000) | 46 | 20000 (20000-20000) | 1 | 24 | 79850 (21200-418275) | 45 | 310400 (80850-1195450) | 1 | - | - | - | - | - | - | - | - | - | - |
| IgM titer | 23 | 1.6 (1.6-7.4) | 44 | 1.6 (1.6-4.8) | 1 | 26 | 47.8 (23.6-135.9) | 43 | 45.7 (26.5-130.9) | 1 | - | - | - | - | - | - | - | - | - | - |
| **Antibody avidity** |  |  |  |  |  |  |  |  |  |  |  |  |  |  |  |  |  |  |  |  |
| IgG1 AI | - | - | - | - | - | 23 | 57.0 (28.5-72.5) | 44 | 53.5 (33.3-82.3) | 1 | - | - | - | - | - | - | - | - | - | - |
| IgG1 AI (biotinylated) | - | - | - | - | - | 24 | 10.0 (2.8-36.3) | 39 | 28.0 (13.5-47.0) | 1 | 20 | 15.5 (3.0-42.5) | 34 | 24.5 (14.0-41.8) | 1 | 19 | 18.0 (5.0-48.5) | 33 | 23.0 (12.0-37.0) | 1 |
| IgG2 AI (biotinylated) | - | - | - | - | - | 26 | 26.0 (11.3-53.5) | 44 | 40.0 (22.0-56.8) | 1 | 21 | 25.0 (11.0-44.0) | 41 | 33.0 (13.0-51.0) | 1 | 21 | 26.0 (7.0-49.0) | 36 | 41.0 (25.0-52.3) | 1 |
| IgG3 AI (biotinylated) | - | - | - | - | - | 13 | 21.0 (12.0-40.0) | 30 | 29.5 (23.3-46.3) | 1 | 9 | 25.0 (16.0-44.0) | 20 | 37.0 (18.8-45.5) | 1 | 8 | 31.0 (15.0-45.3) | 18 | 35.0 (26.3-41.8) | 1 |
| IgA AI | - | - | - | - | - | 21 | 14.0 (12.0-24.0) | 30 | 21.0 (14.5-30.8) | 1 | - | - | - | - | - | - | - | - | - | - |
| IgA AI (biotinylated) | - | - | - | - | - | 26 | 29.5 (23.0-45.5) | 45 | 41.0 (28.0-54.0) | 1 | 21 | 34.0 (20.0-48.0) | 40 | 36.0 (26.0-48.8) | 1 | 21 | 34.0 (22.0-45.0) | 38 | 40.5 (26.5-54.5) | 1 |
| IgA1 AI | - | - | - | - | - | 22 | 15.0 (7.3-17.8) | 46 | 17.5 (11.0-23.0) | 1 | - | - | - | - | - | - | - | - | - | - |
| IgA2 AI | - | - | - | - | - | 18 | 7.5 (5.3-18.8) | 41 | 15.0 (5.0-27.0) | 1 | - | - | - | - | - | - | - | - | - | - |
| **Functional properties** |  |  |  |  |  |  |  |  |  |  |  |  |  |  |  |  |  |  |  |  |
| ADCD (biotinylated) | 26 | 0.0 (0.0-0.2) | 46 | 0.0 (0.0-0.0) | 1 | 26 | 3.3 (1.0-12.9) | 46 | 5.6 (1.6-15.0) | 1 | 20 | 1.7 (0.3-9.0) | 39 | 4.9 (1.8-12.3) | 1 | 20 | 0.6 (0.0-3.4) | 38 | 1.4 (0.0-3.9) | 1 |
| ADCP (biotinylated) | 26 | 0.9 (0.4-1.4) | 46 | 1.0 (0.8-1.4) | 1 | 26 | 1.8 (0.9-3.8) | 46 | 2.3 (1.6-3.8) | 1 | 21 | 1.5 (0.8-3.6) | 41 | 1.9 (1.3-3.8) | 1 | 21 | 1.2 (0.9-3.4) | 38 | 1.7 (1.2-2.8) | 1 |
| ADNP (biotinylated) | 26 | 8.0 (3.9-18.6) | 46 | 7.6 (2.5-13.3) | 1 | 26 | 45.4 (19.8-93.9) | 46 | 91.2 (34.1-146.7) | 1 | 21 | 19.9 (13.6-70.8) | 41 | 40.6 (14.4-109.1) | 1 | 21 | 15.7 (10.5-44.5) | 38 | 31.6 (13.5-76.9) | 1 |
| ADNOB (biotinylated) | 25 | 129.0 (18.5-249.5) | 46 | 113.5 (12.1-192.9) | 1 | 24 | -111.3 (-210.1-1051.5) | 41 | 451.5 (-336.5-1480.0) | 1 | - | - | - | - | - | - | - | - | - | - |
| ADNKA CD107a (biotinylated) | 26 | 6.4 (5.7-7.2) | 43 | 6.6 (5.4-7.7) | 1 | 24 | 6.9 (5.7-8.1) | 45 | 7.4 (6.0-8.6) | 1 | 21 | 7.0 (5.5-7.5) | 38 | 7.0 (6.3-8.5) | 1 | 21 | 7.2 (5.8-8.2) | 35 | 7.3 (6.0-8.3) | 1 |
| ADNKA MIP-1$\beta$ (biotinylated) | 26 | 5.6 (5.2-6.7) | 43 | 5.5 (4.1-6.9) | 1 | 24 | 9.3 (7.8-15.5) | 45 | 10.2 (6.9-13.9) | 1 | 21 | 8.8 (6.4-11.8) | 38 | 9.9 (5.2-13.8) | 1 | 21 | 9.1 (6.9-10.5) | 35 | 9.3 (6.7-12.3) | 1 |
| ADNKA IFN$\gamma$ (biotinylated) | 26 | 8.0 (5.9-11.6) | 43 | 6.0 (4.2-7.9) | 0.80 | 24 | 9.1 (6.5-12.9) | 45 | 6.2 (5.1-9.1) | 0.34 | 21 | 8.7 (6.6-11.6) | 38 | 7.5 (5.2-10.1) | 1 | 21 | 11.4 (7.3-12.9) | 35 | 6.8 (4.9-11.9) | 0.57 |
| **Fc receptor binding** |  |  |  |  |  |  |  |  |  |  |  |  |  |  |  |  |  |  |  |  |
| Fc$\alpha$R (biotinylated) | 26 | 1431 (1206-2261) | 46 | 1353 (1096-1981) | 1 | 26 | 7663 (4549-16095) | 46 | 13798 (8280-22721) | 1 | - | - | - | - | - | - | - | - | - | - |
| Fc$\gamma$R2A binding (biotinylated) | 26 | 5488 (3721-7303) | 46 | 4711 (2791-6482) | 1 | 26 | 15345 (10286-28030) | 46 | 22161 (11098-34907) | 1 | - | - | - | - | - | - | - | - | - | - |
| Fc$\gamma$R2B binding (biotinylated) | 26 | 2369 (1780-2801) | 46 | 1844 (1356-2589) | 1 | 26 | 3751 (2555-6246) | 46 | 3748 (2147-7322) | 1 | - | - | - | - | - | - | - | - | - | - |
| Fc$\gamma$R3A binding (biotinylated) | 26 | 1965 (1334-2649) | 46 | 1404 (1133-2332) | 1 | 26 | 6737 (3182-16119) | 46 | 7647 (2960-16099) | 1 | - | - | - | - | - | - | - | - | - | - |
| Fc$\gamma$R3B binding (biotinylated) | 26 | 1351 (1059-1971) | 46 | 1005 (850-1488) | 0.51 | 26 | 3929 (2067-9217) | 46 | 4740 (1840-12322) | 1 | - | - | - | - | - | - | - | - | - | - |
