# Supplementary material for: Vi-specific serological correlates of protection for typhoid fever
Source: J Exp Med. 2020 Nov 12;218(2):e20201116. doi: 10.1084/jem.20201116 (PMC7668386; doi:10.1084/jem.20201116)
Supplement: Table S4 — presents a comparison of VI-specific humoral responses between diagnosed and protected participants using fold change in responses. [file JEM_20201116_TableS4.docx]

**Table S4 A. Comparison of fold change in Vi-specific measures between diagnosed and protected individuals without P value adjustment**

Fold change using baseline values were calculated for each of the postvaccination time points. Comparisons of fold change between diagnosed and protected individuals were performed using Mann Whitney *U* tests. Presented P values are unadjusted for multiple testing (non-significant P values >0.05 were rounded to two decimal places). Bolded P values are statistically significant.

|  | **Day 28** | | | | | **Day 118** | | | | | **Day 208** | | | | |
| --- | --- | --- | --- | --- | --- | --- | --- | --- | --- | --- | --- | --- | --- | --- | --- |
|  | **Diagnosed** | | **Protected** | | **P value** | **Diagnosed** | | **Protected** | | **P value** | **Diagnosed** | | **Protected** | | **P value** |
|  | ***n*** | **Median (IQR)** | ***n*** | **Median (IQR)** |  | ***n*** | **Median (IQR)** | ***n*** | **Median (IQR)** |  | ***n*** | **Median (IQR)** | ***n*** | **Median (IQR)** |  |
| **Antibody quantification** |  |  |  |  |  |  |  |  |  |  |  |  |  |  |  |
| IgG titer | 26 | 38.5 (11.6-100.9) | 46 | 88.8 (27.2-162.8) | **0.033** | 21 | 22.8 (14.9-38.4) | 41 | 68.8 (20.8-118.3) | **0.012** | 22 | 19.9 (12.7-34.3) | 41 | 56.2 (24.6-141.2) | **0.002** |
| IgG1 titer | - | - | - | - | - | - | - | - | - | - | - | - | - | - | - |
| IgG1 MFI | 26 | 17.2 (4.9-82.3) | 46 | 50.1 (13.2-104.6) | 0.18 | - | - | - | - | - | - | - | - | - | - |
| IgG1 MFI (biotinylated) | 26 | 6.6 (2.2-13.0) | 46 | 8.3 (1.9-30.7) | 0.55 | 21 | 4.8 (2.1-10.5) | 41 | 6.7 (1.4-20.5) | 0.64 | 21 | 3.3 (2.2-9.3) | 38 | 5.8 (1.3-14.9) | 0.51 |
| IgG2 titer | - | - | - | - | - | - | - | - | - | - | - | - | - | - | - |
| IgG2 MFI | 26 | 10.5 (1.5-19.3) | 46 | 20.8 (4.5-42.1) | 0.08 | - | - | - | - | - | - | - | - | - | - |
| IgG2 MFI (biotinylated) | 26 | 14.0 (2.9-40.0) | 46 | 15.3 (4.9-34.6) | 0.47 | 21 | 14.4 (7.2-62.9) | 40 | 27.5 (11.8-84.9) | 0.43 | 21 | 13.1 (4.9-47.7) | 37 | 24.2 (7.4-68.0) | 0.44 |
| IgG3 titer | - | - | - | - | - | - | - | - | - | - | - | - | - | - | - |
| IgG3 MFI (biotinylated) | 26 | 1.0 (1.0-2.2) | 46 | 1.6 (1.0-3.0) | 0.30 | 21 | 1.0 (1.0-2.6) | 41 | 1.2 (1.0-2.4) | 0.46 | 21 | 1.0 (1.0-1.8) | 38 | 1.0 (1.0-2.4) | 0.33 |
| IgA titer | 23 | 6.4 (3.9-18.8) | 44 | 39.6 (18.6-93.8) | **< 0.001** | 21 | 5.1 (2.4-20.5) | 39 | 26.0 (13.4-51.9) | **0.003** | 20 | 6.0 (2.5-22.8) | 39 | 24.6 (12.1-51.3) | **0.003** |
| IgA MFI | 25 | 32.4 (11.0-125.3) | 41 | 113.6 (53.4-267.0) | **0.003** | - | - | - | - | - | - | - | - | - | - |
| IgA MFI (biotinylated) | 26 | 29.6 (9.5-110.1) | 45 | 105.9 (38.5-507.3) | **0.005** | 21 | 11.12 (5.7-57.4) | 41 | 64.1 (31.4-199.1) | **0.005** | 21 | 7.8 (4.4-62.1) | 38 | 73.7 (29.3-182.9) | **0.002** |
| IgA1 MFI | 23 | 10.5 (4.9-25.3) | 46 | 31.5 (13.8-72.7) | **0.008** | - | - | - | - | - | - | - | - | - | - |
| IgA2 MFI | 24 | 4.0 (1.1-20.9) | 45 | 15.5 (4.0-59.8) | **0.032** | - | - | - | - | - | - | - | - | - | - |
| IgM titer | 23 | 9.4 (6.0-56.8) | 43 | 19.0 (10.1-35.9) | 0.17 | - | - | - | - | - | - | - | - | - | - |
| **Functional properties** |  |  |  |  |  |  |  |  |  |  |  |  |  |  |  |
| ADCD (biotinylated) | 7 | 9.3 (4.3-35.2) | 5 | 22.6 (2.5-28.6) | 0.87 | 6 | 5.3 (1.8-24.5) | 5 | 11.7 (2.1-12.7) | 0.93 | 6 | 2.1 (1.4-7.3) | 4 | 7.6 (3.7-11.2) | 0.34 |
| ADCP (biotinylated) | 26 | 2.1 (1.6-2.8) | 46 | 1.9 (1.3-3.9) | 0.52 | 21 | 2.3 (1.4-5.0) | 41 | 1.9 (1.0-3.1) | 0.41 | 21 | 1.7 (0.9-5.2) | 38 | 1.9 (0.8-3.5) | 0.67 |
| ADNP (biotinylated) | 25 | 3.6 (2.3-10.6) | 43 | 8.7 (3.8-33.1) | 0.06 | 20 | 2.5 (1.0-8.5) | 38 | 5.9 (2.6-22.4) | **0.047** | 20 | 1.6 (0.9-4.2) | 35 | 6.2 (1.3-13.0) | 0.06 |
| ADNOB (biotinylated) | 23 | -1.6 (-9.6- -0.5) | 41 | 1.1 (-2.9-13.0) | **0.015** | - | - | - | - | - | - | - | - | - | - |
| ADNKA CD107a (biotinylated) | 24 | 1.1 (0.9-1.2) | 43 | 1.1 (1.0-1.3) | 0.54 | 21 | 1.0 (0.9-1.2) | 38 | 1.1 (0.9-1.3) | 0.34 | 21 | 1.1 (1.0-1.3) | 35 | 1.1 (0.9-1.3) | 0.76 |
| ADNKA MIP-1$\beta$ (biotinylated) | 24 | 1.6 (1.4-2.5) | 43 | 2.2 (1.2-2.9) | 0.42 | 21 | 1.7 (1.2-2.0) | 38 | 1.8 (1.2-2.8) | 0.31 | 21 | 1.6 (1.1-1.9) | 35 | 1.6 (1.1-2.2) | 0.48 |
| ADNKA IFN$\gamma$ (biotinylated) | 24 | 1.3 (1.0-1.5) | 43 | 1.2 (0.8-1.4) | 0.356 | 21 | 1.1 (1.0-1.6) | 38 | 1.3 (1.0-1.6) | 0.56 | 21 | 1.4 (1.1-1.7) | 35 | 1.2 (1.0-1.7) | 0.32 |
| **Fc Receptor binding** |  |  |  |  |  |  |  |  |  |  |  |  |  |  |  |
| Fc$\alpha$R (biotinylated) | 26 | 5.3 (2.8-9.2) | 46 | 9.1 (5.5-18.1) | **0.009** | - | - | - | - | - | - | - | - | - | - |
| Fc$\gamma$R2A binding (biotinylated) | 26 | 3.5 (1.9-4.6) | 46 | 4.6 (2.8-7.0) | **0.030** | - | - | - | - | - | - | - | - | - | - |
| Fc$\gamma$R2B binding (biotinylated) | 26 | 1.7 (1.3-2.7) | 46 | 2.0 (1.3-3.6) | 0.24 | - | - | - | - | - | - | - | - | - | - |
| Fc$\gamma$R3A binding (biotinylated) | 26 | 4.1 (1.6-6.7) | 46 | 5.2 (2.5-8.9) | 0.19 | - | - | - | - | - | - | - | - | - | - |
| Fc$\gamma$R3B binding (biotinylated) | 26 | 2.6 (1.3-7.8) | 46 | 4.0 (2.2-8.2) | 0.17 | - | - | - | - | - | - | - | - | - | - |

**Table S4 B. Comparison of fold-change in Vi-specific measures between diagnosed and protected individuals**

Fold-change in Vi-specific measures from baseline were calculated for each of the postvaccination time points. Comparisons of fold-change between diagnosed and protected individuals were performed using Mann Whitney U tests. Presented P values were adjusted for multiple testing using the Bonferroni correction method (non-significant P values >0.05 were rounded to two decimal places).

|  | **Day 28** | | | | | **Day 118** | | | | | **Day 208** | | | | |
| --- | --- | --- | --- | --- | --- | --- | --- | --- | --- | --- | --- | --- | --- | --- | --- |
|  | **Diagnosed** | | **Protected** | | **P value** | **Diagnosed** | | **Protected** | | **P value** | **Diagnosed** | | **Protected** | | **P value** |
|  | ***n*** | **Median (IQR)** | ***n*** | **Median (IQR)** |  | ***n*** | **Median (IQR)** | ***n*** | **Median (IQR)** |  | ***n*** | **Median (IQR)** | ***n*** | **Median (IQR)** |  |
| **Antibody quantification** |  |  |  |  |  |  |  |  |  |  |  |  |  |  |  |
| IgG titer | 26 | 38.5 (11.6-100.9) | 46 | 88.8 (27.2-162.8) | 1 | 21 | 22.8 (14.9-38.4) | 41 | 68.8 (20.8-118.3) | 0.17 | 22 | 19.9 (12.7-34.3) | 41 | 56.2 (24.6-141.2) | **0.023** |
| IgG1 titer | - | - | - | - | - | - | - | - | - | - | - | - | - | - | - |
| IgG1 MFI | 26 | 17.2 (4.9-82.3) | 46 | 50.1 (13.2-104.6) | 1 | - | - | - | - | - | - | - | - | - | - |
| IgG1 MFI (biotinylated) | 26 | 6.6 (2.2-13.0) | 46 | 8.3 (1.9-30.7) | 1 | 21 | 4.8 (2.1-10.5) | 41 | 6.7 (1.4-20.5) | 1 | 21 | 3.3 (2.2-9.3) | 38 | 5.8 (1.3-14.9) | 1 |
| IgG2 titer | - | - | - | - | - | - | - | - | - | - | - | - | - | - | - |
| IgG2 MFI | 26 | 10.5 (1.5-19.3) | 46 | 20.8 (4.5-42.1) | 1 | - | - | - | - | - | - | - | - | - | - |
| IgG2 MFI (biotinylated) | 26 | 14.0 (2.9-40.0) | 46 | 15.3 (4.9-34.6) | 1 | 21 | 14.4 (7.2-62.9) | 40 | 27.5 (11.8-84.9) | 1 | 21 | 13.1 (4.9-47.7) | 37 | 24.2 (7.4-68.0) | 1 |
| IgG3 titer | - | - | - | - | - | - | - | - | - | - | - | - | - | - | - |
| IgG3 MFI (biotinylated) | 26 | 1.0 (1.0-2.2) | 46 | 1.6 (1.0-3.0) | 1 | 21 | 1.0 (1.0-2.6) | 41 | 1.2 (1.0-2.4) | 1 | 21 | 1.0 (1.0-1.8) | 38 | 1.0 (1.0-2.4) | 1 |
| IgA titer | 23 | 6.4 (3.9-18.8) | 44 | 39.6 (18.6-93.8) | **0.021** | 21 | 5.1 (2.4-20.5) | 39 | 26.0 (13.4-51.9) | **0.037** | 20 | 6.0 (2.5-22.8) | 39 | 24.6 (12.1-51.3) | **0.049** |
| IgA MFI | 25 | 32.4 (11.0-125.3) | 41 | 113.6 (53.4-267.0) | 0.08 | - | - | - | - | - | - | - | - | - | - |
| IgA MFI (biotinylated) | 26 | 29.6 (9.5-110.1) | 45 | 105.9 (38.5-507.3) | 0.14 | 21 | 11.12 (5.7-57.4) | 41 | 64.1 (31.4-199.1) | 0.07 | 21 | 7.8 (4.4-62.1) | 38 | 73.7 (29.3-182.9) | **0.021** |
| IgA1 MFI | 23 | 10.5 (4.9-25.3) | 46 | 31.5 (13.8-72.7) | 0.23 | - | - | - | - | - | - | - | - | - | - |
| IgA2 MFI | 24 | 4.0 (1.1-20.9) | 45 | 15.5 (4.0-59.8) | 0.97 | - | - | - | - | - | - | - | - | - | - |
| IgM titer | 23 | 9.4 (6.0-56.8) | 43 | 19.0 (10.1-35.9) | 1 | - | - | - | - | - | - | - | - | - | - |
| **Functional properties** |  |  |  |  |  |  |  |  |  |  |  |  |  |  |  |
| ADCD (biotinylated) | 7 | 9.3 (4.3-35.2) | 5 | 22.6 (2.5-28.6) | 1 | 6 | 5.3 (1.8-24.5) | 5 | 11.7 (2.1-12.7) | 1 | 6 | 2.1 (1.4-7.3) | 4 | 7.6 (3.7-11.2) | 1 |
| ADCP (biotinylated) | 26 | 2.1 (1.6-2.8) | 46 | 1.9 (1.3-3.9) | 1 | 21 | 2.3 (1.4-5.0) | 41 | 1.9 (1.0-3.1) | 1 | 21 | 1.7 (0.9-5.2) | 38 | 1.9 (0.8-3.5) | 1 |
| ADNP (biotinylated) | 25 | 3.6 (2.3-10.6) | 43 | 8.7 (3.8-33.1) | 1 | 20 | 2.5 (1.0-8.5) | 38 | 5.9 (2.6-22.4) | 0.66 | 20 | 1.6 (0.9-4.2) | 35 | 6.2 (1.3-13.0) | 0.78 |
| ADNOB (biotinylated) | 23 | -1.6 (-9.6- -0.5) | 41 | 1.1 (-2.9-13.0) | 0.45 | - | - | - | - | - | - | - | - | - | - |
| ADNKA CD107a (biotinylated) | 24 | 1.1 (0.9-1.2) | 43 | 1.1 (1.0-1.3) | 1 | 21 | 1.0 (0.9-1.2) | 38 | 1.1 (0.9-1.3) | 1 | 21 | 1.1 (1.0-1.3) | 35 | 1.1 (0.9-1.3) | 1 |
| ADNKA MIP-1$\beta$ (biotinylated) | 24 | 1.6 (1.4-2.5) | 43 | 2.2 (1.2-2.9) | 1 | 21 | 1.7 (1.2-2.0) | 38 | 1.8 (1.2-2.8) | 1 | 21 | 1.6 (1.1-1.9) | 35 | 1.6 (1.1-2.2) | 1 |
| ADNKA IFN$\gamma$ (biotinylated) | 24 | 1.3 (1.0-1.5) | 43 | 1.2 (0.8-1.4) | 1 | 21 | 1.1 (1.0-1.6) | 38 | 1.3 (1.0-1.6) | 1 | 21 | 1.4 (1.1-1.7) | 35 | 1.2 (1.0-1.7) | 1 |
| **Fc receptor binding** |  |  |  |  |  |  |  |  |  |  |  |  |  |  |  |
| Fc$\alpha$R (biotinylated) | 26 | 5.3 (2.8-9.2) | 46 | 9.1 (5.5-18.1) | 0.28 | - | - | - | - | - | - | - | - | - | - |
| Fc$\gamma$R2A binding (biotinylated) | 26 | 3.5 (1.9-4.6) | 46 | 4.6 (2.8-7.0) | 0.89 | - | - | - | - | - | - | - | - | - | - |
| Fc$\gamma$R2B binding (biotinylated) | 26 | 1.7 (1.3-2.7) | 46 | 2.0 (1.3-3.6) | 1 | - | - | - | - | - | - | - | - | - | - |
| Fc$\gamma$R3A binding (biotinylated) | 26 | 4.1 (1.6-6.7) | 46 | 5.2 (2.5-8.9) | 1 | - | - | - | - | - | - | - | - | - | - |
| Fc$\gamma$R3B binding (biotinylated) | 26 | 2.6 (1.3-7.8) | 46 | 4.0 (2.2-8.2) | 1 | - | - | - | - | - | - | - | - | - | - |
