# Supplementary material for: Vi-specific serological correlates of protection for typhoid fever
Source: J Exp Med. 2020 Nov 12;218(2):e20201116. doi: 10.1084/jem.20201116 (PMC7668386; doi:10.1084/jem.20201116)
Supplement: Table S5 — compares Vi-specific responses between diagnosed and protected participants vaccinated with Vi-TT and between diagnosed and protected Vi-PS vaccines, using absolute values. [file JEM_20201116_TableS5.docx]

**Table S5 A. Comparison of Vi-specific measures between diagnosed and protected individuals following Vi-TT vaccination**

Comparisons of Vi-specific measures between diagnosed and protected individuals were performed using Mann Whitney *U* tests. Presented P values have been adjusted for multiple testing using the Bonferroni correction method (non-significant P values >0.05 were rounded to two decimal places).

|  | **Baseline** | | | | | **Day 28** | | | | | **Day 118** | | | | | **Day 208** | | | | |
| --- | --- | --- | --- | --- | --- | --- | --- | --- | --- | --- | --- | --- | --- | --- | --- | --- | --- | --- | --- | --- |
|  | **Diagnosed** | | **Protected** | | **P value** | **Diagnosed** | | **Protected** | | **P value** | **Diagnosed** | | **Protected** | | **P value** | **Diagnosed** | | **Protected** | | **P value** |
|  | ***n*** | **Median (IQR)** | ***n*** | **Median (IQR)** |  | ***n*** | **Median (IQR)** | ***n*** | **Median (IQR)** |  | ***n*** | **Median (IQR)** | ***n*** | **Median (IQR)** |  | ***n*** | **Median (IQR)** | ***n*** | **Median (IQR)** |  |
| **Antibody quantification** |  |  |  |  |  |  |  |  |  |  |  |  |  |  |  |  |  |  |  |  |
| IgG titer | 13 | 3.7 (3.7-8.1) | 24 | 3.7 (3.7-3.7) | 1 | 13 | 489.0 (249.2-1046.3) | 24 | 658.6 (291.2-1229.4) | 1 | 11 | 200.5 (87.8-579.2) | 22 | 310.2 (224.5-597.7) | 1 | 11 | 118.8 (70.4-583.5) | 23 | 348.3 (176.7-829.9) | 1 |
| IgG1 titer | - | - | - | - | - | 13 | 90.7 (43.0-259.3) | 24 | 90.7 (44.5-171.3) | 1 | - | - | - | - | - | - | - | - | - | - |
| IgG1 MFI | 13 | 10000 (10000-10000) | 24 | 10000 (10000-10000) | 0.48 | 13 | 1213900 (573775-1492775) | 24 | 722050 (437224-1351444) | 1 | - | - | - | - | - | - | - | - | - | - |
| IgG1 MFI (biotinylated) | 13 | 10000 (10000-49450) | 24 | 10000 (10000-10000) | 0.87 | 13 | 262600 (152450-696700) | 24 | 238175 (96169-613079) | 1 | 10 | 454463 (178538-543433) | 22 | 1935689 (62327-321869) | 1 | 11 | 162950 (87675-499650) | 21 | 123925 (44725-224250) | 1 |
| IgG2 titer | - | - | - | - | - | 13 | 40.3 (18.1-75.7) | 24 | 48.3 (23.7-141.7) | 1 | - | - | - | - | - | - | - | - | - | - |
| IgG2 MFI | 13 | 50000 (50000-50000) | 24 | 50000 (50000-50000) | 1 | 13 | 963625 (689000-3135375) | 24 | 1329000 (853563-2979594) | 1 | - | - | - | - | - | - | - | - | - | - |
| IgG2 MFI (biotinylated) | 13 | 4000 (4000-4050) | 24 | 4490 (4000-13503) | 1 | 13 | 159270 (61120-248380) | 24 | 176900 (78358-492748) | 1 | 10 | 409210 (98388-754078) | 21 | 346760 (136310-2248500) | 1 | 11 | 306810 (39390-571170) | 20 | 275475 (64878-605028) | 1 |
| IgG3 titer | - | - | - | - | - | 13 | 106.5 (48.4-223.4) | 24 | 126.0 (67.4-296.6) | 1 | - | - | - | - | - | - | - | - | - | - |
| IgG3 MFI (biotinylated) | 13 | 5000 (5000-5000) | 24 | 5000 (5000-5000) | - | 13 | 10850 (5000-19938) | 24 | 13638 (6556-20281) | 1 | 10 | 13050 (6306-18316) | 22 | 10406 (6444-14984) | 1 | 11 | 8763 (5000-16188) | 21 | 10213 (5000-15888) | 1 |
| IgA titer | 10 | 1.6 (1.6-1.6) | 23 | 1.6 (1.6-1.6) | 1 | 13 | 88.3 (28.2-148.6) | 23 | 94.4 (57.9-201.6) | 1 | 11 | 36.8 (8.1-74.7) | 22 | 59.5 (25.2-130.0) | 1 | 11 | 31.5 (9.4-75.5) | 22 | 61.4 (23.6-122.5) | 1 |
| IgA MFI | 13 | 5000 (5000-5000) | 24 | 5000 (5000-5000) | 1 | 12 | 663913 (198084-1067200) | 20 | 1037319 (438191-2923300) | 1 | - | - | - | - | - | - | - | - | - | - |
| IgA MFI (biotinylated) | 13 | 15150 (5000-40162) | 24 | 6069 (5000-12528) | 1 | 13 | 809138 (395875-4919100) | 24 | 2319650 (554100-5393025) | 1 | 10 | 686038 (104756-1669497) | 22 | 839588 (397338-1404797) | 1 | 11 | 476725 (58032-879538) | 21 | 763125 (318712-1101600) | 1 |
| IgA1 MFI | 11 | 20000 (20000-20000) | 24 | 20000 (20000-20000) | 1 | 11 | 457350 (172650-1406325) | 24 | 1151250 (490888-1811013) | 1 | - | - | - | - | - | - | - | - | - | - |
| IgA2 MFI | 12 | 20000 (20000-20000) | 24 | 20000 (20000-20000) | - | 12 | 185825 (38425-456400) | 24 | 283250 (104413-1675625) | 1 | - | - | - | - | - | - | - | - | - | - |
| IgM titer | 10 | 1.6 (1.6-1.6) | 23 | 1.6 (1.6-3.2) | 1 | 13 | 114.9 (55.0-224.3) | 22 | 58.6 (29.9-215.9) | 1 | - | - | - | - | - | - | - | - | - | - |
| **Antibody avidity** |  |  |  |  |  |  |  |  |  |  |  |  |  |  |  |  |  |  |  |  |
| IgG1 AI | - | - | - | - | - | 13 | 69.0 (57.0-77.0) | 24 | 74.5 (54.8-99.3) | 1 | - | - | - | - | - | - | - | - | - | - |
| IgG1 AI (biotinylated) | - | - | - | - | - | 12 | 25.0 (8.8-36.5) | 24 | 32.0 (20.0-51.0) | 1 | 9 | 37.0 (20.0-56.0) | 21 | 28.0 (22.0-49.0) | 1 | 10 | 37.0 (11.3-51.8) | 21 | 28.0 (19.0-47.0) | 1 |
| IgG2 AI (biotinylated) | - | - | - | - | - | 13 | 27.0 (25.0-56.0) | 23 | 48.0 (30.0-65.5) | 1 | 10 | 39.0 (23.5-43.5) | 22 | 34.5 (27.8-49.0) | 1 | 11 | 37.0 (21.0-50.0) | 20 | 46.0 (27.3-60.3) | 1 |
| IgG3 AI (biotinylated) | - | - | - | - | - | 9 | 35.0 (21.0-40.0) | 21 | 37.0 (27.0-48.0) | 1 | 7 | 42.0 (22.5-50.0) | 16 | 37.0 (22.0-47.3) | 1 | 7 | 39.0 (20.0-47.5) | 15 | 34.0 (26.5-45.5) | 1 |
| IgA AI | - | - | - | - | - | 11 | 15.0 (12.5-27.0) | 16 | 24.5 (17.5-38.5) | 1 | - | - | - | - | - | - | - | - | - | - |
| IgA AI (biotinylated) | - | - | - | - | - | 13 | 27.0 (23.0-46.0) | 24 | 46.0 (32.0-64.3) | 1 | 10 | 34.0 (22.5-52.8) | 21 | 44.0 (27.0-58.0) | 1 | 11 | 30.0 (20.0-50.5) | 21 | 45.0 (28.0-60.0) | 1 |
| IgA1 AI | - | - | - | - | - | 11 | 16.0 (8.5-17.5) | 24 | 20.0 (12.8-32.8) | 1 | - | - | - | - | - | - | - | - | - | - |
| IgA2 AI | - | - | - | - | - | 11 | 6.0 (4.5-16.0) | 24 | 20.0 (5.0-30.8) | 1 | - | - | - | - | - | - | - | - | - | - |
| **Functional properties** |  |  |  |  |  |  |  |  |  |  |  |  |  |  |  |  |  |  |  |  |
| ADCD (biotinylated) | 13 | 0.0 (0.0-0.9) | 24 | 0.0 (0.0-0.0) | 1 | 13 | 7.7 (2.2-20.1) | 24 | 7.4 (2.8-16.9) | 1 | 10 | 6.1 (1.6-15.8) | 21 | 5.8 (4.2-13.9) | 1 | 11 | 0.8 (0.0-6.9) | 20 | 1.5 (0.4-5.3) | 1 |
| ADCP (biotinylated) | 13 | 1.3 (1.0-1.8) | 24 | 0.8 (0.7-1.4) | 1 | 13 | 3.2 (2.0-5.2) | 24 | 2.7 (1.7-3.8) | 1 | 10 | 3.1 (2.1-5.3) | 22 | 2.4 (1.6-3.9) | 1 | 11 | 1.9 (1.1-3.8) | 21 | 1.8 (1.0-2.9) | 1 |
| ADNP (biotinylated) | 13 | 12.6 (4.2-23.5) | 24 | 8.0 (2.4-14.5) | 1 | 13 | 89.5 (50.6-108.0) | 24 | 111.2 (66.6-183.7) | 1 | 10 | 73.5 (20.4-129.5) | 22 | 63.2 (28.4-135.4) | 1 | 11 | 27.2 (8.5-51.4) | 21 | 32.8 (19.6-86.4) | 1 |
| ADNOB (biotinylated) | 13 | 33.0 (-55.5-138.5) | 24 | 121.8 (31.8-192.6) | 1 | 11 | 743.5 (-180.8-1449.8) | 22 | 988.8 (102.5-1469.0) | 1 | - | - | - | - | - | - | - | - | - | - |
| ADNKA CD107a (biotinylated) | 13 | 6.8 (6.3-8.2) | 23 | 6.6 (5.9-7.3) | 1 | 11 | 6.6 (5.9-7.5) | 24 | 7.8 (6.5-9.0) | 1 | 10 | 7.4 (5.7-7.5) | 21 | 7.2 (6.5-9.0) | 1 | 11 | 7.7 (7.0-8.6) | 20 | 7.3 (6.3-8.4) | 1 |
| ADNKA MIP-1$\beta$ (biotinylated) | 13 | 5.9 (4.7-7.7) | 23 | 5.3 (3.9-6.5) | 1 | 11 | 14.5 (9.4-19.4) | 24 | 11.7 (8.1-13.6) | 1 | 10 | 10.9 (7.7-15.4) | 21 | 10.6 (5.3-14.0) | 1 | 11 | 10.4 (6.4-12.8) | 20 | 9.0 (7.2-12.3) | 1 |
| ADNKA IFN$\gamma$ (biotinylated) | 13 | 8.3 (7.5-12.1) | 23 | 5.1 (4.0-7.4) | **0.019** | 11 | 11.2 (8.7-14.2) | 24 | 5.6 (5.0-8.5) | 0.341 | 10 | 11.2 (8.5-13.1) | 21 | 7.4 (5.1-9.6) | 0.30 | 11 | 12.4 (9.6-13.9) | 20 | 6.4 (4.8-10.2) | 0.18 |
| **Fc receptor binding** |  |  |  |  |  |  |  |  |  |  |  |  |  |  |  |  |  |  |  |  |
| Fc$\alpha$R (biotinylated) | 13 | 1440 (1186-2173) | 24 | 1361 (1064-1975) | 1 | 13 | 13023 (5319-21398) | 24 | 17866 (9837-31007) | 1 | - | - | - | - | - | - | - | - | - | - |
| Fc$\gamma$R2A binding (biotinylated) | 13 | 5236 (3714-6457) | 24 | 4916 (2673-7391) | 1 | 13 | 27220 (21389-31111) | 24 | 30899 (19918-39032) | 1 | - | - | - | - | - | - | - | - | - | - |
| Fc$\gamma$R2B binding (biotinylated) | 13 | 2403 (1868-2514) | 24 | 1768 (1241-2747) | 1 | 13 | 6257 (3220-7636) | 24 | 5952 (3695-10585) | 1 | - | - | - | - | - | - | - | - | - | - |
| Fc$\gamma$R3A binding (biotinylated) | 13 | 2073 (1335-2545) | 24 | 1400 (1084-2191) | 1 | 13 | 14486 (9692-17983) | 24 | 13213 (8739-19334) | 1 | - | - | - | - | - | - | - | - | - | - |
| Fc$\gamma$R3B binding (biotinylated) | 13 | 1472 (920-1665) | 24 | 1020 (817-1572) | 1 | 13 | 7929 (5021-14224) | 24 | 9741 (4637-14718) | 1 | - | - | - | - | - | - | - | - | - | - |

**Table S5 B. Comparison of Vi-specific measures between diagnosed and protected individuals following Vi-PS vaccination**

Comparisons of Vi-specific measures between diagnosed and protected individuals were performed using Mann Whitney *U* tests. Presented P values have been adjusted for multiple testing using the Bonferroni correction method (non-significant P values >0.05 were rounded to two decimal places).

|  | **Baseline** | | | | | **Day 28** | | | | | **Day 118** | | | | | **Day 208** | | | | |
| --- | --- | --- | --- | --- | --- | --- | --- | --- | --- | --- | --- | --- | --- | --- | --- | --- | --- | --- | --- | --- |
|  | **Diagnosed** | | **Protected** | | **P value** | **Diagnosed** | | **Protected** | | **P value** | **Diagnosed** | | **Protected** | | **P value** | **Diagnosed** | | **Protected** | | **P value** |
|  | ***n*** | **Median (IQR)** | ***n*** | **Median (IQR)** |  | ***n*** | **Median (IQR)** | ***n*** | **Median (IQR)** |  | ***n*** | **Median (IQR)** | ***n*** | **Median (IQR)** |  | ***n*** | **Median (IQR)** | ***n*** | **Median (IQR)** |  |
| **Antibody quantification** |  |  |  |  |  |  |  |  |  |  |  |  |  |  |  |  |  |  |  |  |
| IgG titer | 13 | 3.7 (3.7-3.7) | 22 | 3.7 (3.7-8.8) | 1 | 13 | 89.4 (62.8-126.0) | 22 | 144.8 (80.1-559.8) | 1 | 10 | 87.2 (68.8-126.8) | 19 | 168.5 (68.1-525.3) | 1 | 11 | 104.3 (59.1-148.1) | 18 | 165.6 (90.7-525.8) | 1 |
| IgG1 titer | - | - | - | - | - | 13 | 10.4 (7.2-14.2) | 22 | 22.5 (5.3-39.3) | 1 | - | - | - | - | - | - | - | - | - | - |
| IgG1 MFI | 13 | 10000 (10000-10000) | 22 | 10000 (10000-10000) | 1 | 13 | 54400 (10000-163750) | 22 | 150888 (47275-815869) | 1 | - | - | - | - | - | - | - | - | - | - |
| IgG1 MFI (biotinylated) | 13 | 10000 (10000-12125) | 22 | 10000 (10000-10000) | 1 | 13 | 35425 (26625-73125) | 22 | 40888 (10000-168256) | 1 | 11 | 37313 (26516-53141) | 19 | 17925 (10000-135981) | 1 | 10 | 31319 (23189-48389) | 17 | 14725 (10000-96750) | 1 |
| IgG2 titer | - | - | - | - | - | 13 | 9.4 (3.0-15.8) | 22 | 17.9 (8.7-65.1) | 0.74 | - | - | - | - | - | - | - | - | - | - |
| IgG2 MFI | 13 | 50000 (50000-50000) | 22 | 50000 (50000-50000) | 1 | 13 | 253000 (50000-513250) | 22 | 480125 (67375-1541969) | 1 | - | - | - | - | - | - | - | - | - | - |
| IgG2 MFI (biotinylated) | 13 | 5100 (4000-24940) | 22 | 4000 (4000-10370) | 1 | 13 | 86240 (11760-201750) | 22 | 49815 (21568-229408) | 1 | 11 | 216150 (52535-306000) | 19 | 109260 (38195-498580) | 1 | 10 | 148125 (33930-350240) | 17 | 136400 (48530-711070) | 1 |
| IgG3 titer | - | - | - | - | - | 13 | 19.1 (15.5-22.4) | 22 | 32.0 (14.9-61.1) | 1 | - | - | - | - | - | - | - | - | - | - |
| IgG3 MFI (biotinylated) | 13 | 5000 (5000-5000) | 22 | 5000 (5000-5000) | 1 | 13 | 5000 (5000-5225) | 22 | 5000 (5000-8678) | 1 | 11 | 5000 (5000-5000) | 19 | 5000 (5000-5000) | 1 | 10 | 5000 (5000-5000) | 17 | 5000 (5000-5000) | 1 |
| IgA titer | 13 | 1.6 (1.6-1.6) | 21 | 1.6 (1.6-1.6) | 1 | 13 | 14.1 (5.8-29.2) | 21 | 36.0 (28.7-108.8) | 0.74 | 11 | 12.3 (3.8-18.6) | 19 | 38.5 (25.5-70.8) | 0.20 | 10 | 13.2 (3.8-19.2) | 18 | 37.3 (23.3-83.0) | 0.28 |
| IgA MFI | 13 | 5000 (5000-5050) | 22 | 5000 (5000-5000) | 1 | 13 | 156200 (28438-194825) | 21 | 411575 (239588-875363) | 0.10 | - | - | - | - | - | - | - | - | - | - |
| IgA MFI (biotinylated) | 13 | 10838 (5262-11600) | 22 | 8513 (5000-18354) | 1 | 13 | 292012 (67950-446725) | 21 | 536388 (297612-724150) | 0.90 | 11 | 231188 (51500-396225) | 19 | 454200 (347669-1030394) | 0.93 | 10 | 255275 (71026-346228) | 17 | 502400 (330988-1087100) | 0.85 |
| IgA1 MFI | 12 | 20000 (20000-21700) | 22 | 20000 (20000-20000) | 1 | 12 | 211025 (79963-380588) | 22 | 367325 (148300-783738) | 1 | - | - | - | - | - | - | - | - | - | - |
| IgA2 MFI | 12 | 20000 (20000-20000) | 22 | 20000 (20000-20000) | - | 12 | 53675 (20000-161200) | 21 | 310400 (48600-526100) | 1 | - | - | - | - | - | - | - | - | - | - |
| IgM titer | 13 | 1.6 (1.6-7.6) | 21 | 1.6 (1.6-5.2) | 1 | 13 | 36.9 (15.0-46.3) | 21 | 37.9 (18.2-89.2) | 1 | - | - | - | - | - | - | - | - | - | - |
| **Antibody avidity** |  |  |  |  |  |  |  |  |  |  |  |  |  |  |  |  |  |  |  |  |
| IgG1 AI | - | - | - | - | - | 10 | 27.0 (20.5-37.5) | 20 | 31.5 (17.5-43.0) | 1 | - | - | - | - | - | - | - | - | - | - |
| IgG1 AI (biotinylated) | - | - | - | - | - | 12 | 3.0 (0.0-17.5) | 15 | 20.0 (3.5-34.0) | 1 | 11 | 4.0 (2.5-15.0) | 13 | 15.0 (3.0-24.0) | 1 | 9 | 9.0 (2.0-18.0) | 12 | 15.0 (6.8-26.0) | 1 |
| IgG2 AI (biotinylated) | - | - | - | - | - | 13 | 20.0 (5.0-36.0) | 21 | 32.0 (22.0-50.0) | 1 | 11 | 18.0 (7.5-41.0) | 19 | 29.0 (12.5-52.0) | 1 | 10 | 14.5 (7.0-43.3) | 16 | 30.5 (22.3-44.8) | 1 |
| IgG3 AI (biotinylated) | - | - | - | - | - | 4 | 4.0 (2.5-6.8) | 9 | 18.0 (6.0-28.0) | 1 | 2 | 6.0 (6.0-6.0) | 4 | 23.0 (5.0-42.0) | 1 | 1 | 4.0 (4.0-4.0) | 3 | 36.0 (18.5-39.0) | 1 |
| IgA AI | - | - | - | - | - | 10 | 13.0 (9.3-23.0) | 14 | 18.5 (9.5-29.0) | 1 | - | - | - | - | - | - | - | - | - | - |
| IgA AI (biotinylated) | - | - | - | - | - | 13 | 30.0 (23.0-41.0) | 21 | 39.0 (19.0-48.0) | 1 | 11 | 34.0 (18.5-41.5) | 19 | 34.0 (20.0-44.0) | 1 | 10 | 35.0 (23.0-43.3) | 17 | 38.0 (21.0-44.0) | 1 |
| IgA1 AI | - | - | - | - | - | 11 | 14.0 (7.5-19.5) | 22 | 13.5 (6.5-19.8) | 1 | - | - | - | - | - | - | - | - | - | - |
| IgA2 AI | - | - | - | - | - | 7 | 14.0 (6.5-20.0) | 17 | 11.0 (7.0-18.0) | 1 | - | - | - | - | - | - | - | - | - | - |
| **Functional properties** |  |  |  |  |  |  |  |  |  |  |  |  |  |  |  |  |  |  |  |  |
| ADCD (biotinylated) | 13 | 0.0 (0.0-0.0) | 22 | 0.0 (0.0-0.0) | 1 | 13 | 2.8 (0.6-3.9) | 22 | 3.7 (1.1-11.4) | 1 | 10 | 1.3 (0.1-2.2) | 18 | 2.3 (0.9-10.2) | 1 | 9 | 0.0 (0.0-1.5) | 18 | 0.6 (0.0-2.2) | 1 |
| ADCP (biotinylated) | 13 | 0.5 (0.2-0.9) | 22 | 1.2 (0.9-1.4) | 0.10 | 13 | 0.9 (0.6-1.5) | 22 | 1.9 (1.5-3.5) | 0.19 | 11 | 0.8 (0.4-1.0) | 19 | 1.8 (1.3-2.8) | 0.14 | 10 | 0.9 (0.3-1.1) | 17 | 1.6 (1.3-2.4) | 0.41 |
| ADNP (biotinylated) | 13 | 7.1 (2.3-8.8) | 22 | 7.4 (2.8-11.1) | 1 | 13 | 20.2 (12.7-43.5) | 22 | 38.4 (11.0-111.4) | 1 | 11 | 14.9 (9.4-20.0) | 19 | 28.5 (7.0-71.2) | 1 | 10 | 13.3 (11.0-20.5) | 17 | 30.4 (5.0-66.9) | 1 |
| ADNOB (biotinylated) | 12 | 170.0 (107.9-258.8) | 22 | 93.8 (5.8-203.4) | 1 | 13 | -144.0 (-248.0-227.0) | 19 | -14.0 (-412.3-1554.8) | 1 | - | - | - | - | - | - | - | - | - | - |
| ADNKA CD107a (biotinylated) | 13 | 6.0 (5.6-7.2) | 20 | 6.8 (5.2-8.0) | 1 | 13 | 7.3 (5.8-8.5) | 21 | 7.2 (5.6-7.7) | 1 | 11 | 7.0 (5.1-7.6) | 17 | 6.8 (5.9-7.8) | 1 | 10 | 7.0 (5.5-7.6) | 15 | 7.3 (5.8-8.1) | 1 |
| ADNKA MIP-1$\beta$ (biotinylated) | 13 | 5.5 (5.3-6.3) | 20 | 5.9 (4.4-7.4) | 1 | 13 | 8.2 (7.5-9.4) | 21 | 10.2 (6.3-15.0) | 1 | 11 | 7.0 (6.2-9.8) | 17 | 9.7 (5.2-13.6) | 1 | 10 | 8.1 (7.1-9.1) | 15 | 9.3 (6.5-11.8) | 1 |
| ADNKA IFN$\gamma$ (biotinylated) | 13 | 5.9 (4.3-8.5) | 20 | 6.5 (4.7-9.9) | 1 | 13 | 8.2 (6.4-9.5) | 21 | 6.8 (5.8-9.2) | 1 | 11 | 7.3 (5.3-9.2) | 17 | 8.6 (5.7-11.8) | 1 | 10 | 8.2 (6.3-11.4) | 15 | 8.1 (5.5-12.3) | 1 |
| **Fc receptor binding** |  |  |  |  |  |  |  |  |  |  |  |  |  |  |  |  |  |  |  |  |
| Fc$\alpha$R (biotinylated) | 13 | 1423 (1267-2523) | 22 | 1336 (1100-1959) | 1 | 13 | 5531 (3715-8508) | 22 | 9117 (5546-18796) | 1 | - | - | - | - | - | - | - | - | - | - |
| Fc$\gamma$R2A binding (biotinylated) | 13 | 5741 (3741-9635) | 22 | 4409 (3320-5509) | 1 | 13 | 10968 (6538-14015) | 22 | 15560 (7203-25855) | 1 | - | - | - | - | - | - | - | - | - | - |
| Fc$\gamma$R2B binding (biotinylated) | 13 | 2309 (1771-2897) | 22 | 1871 (1367-2323) | 1 | 13 | 2791 (1926-4274) | 22 | 2281 (1806-3705) | 1 | - | - | - | - | - | - | - | - | - | - |
| Fc$\gamma$R3A binding (biotinylated) | 13 | 1731 (1334-2684) | 22 | 1404 (1173-2258) | 1 | 13 | 4034 (1862-5115) | 22 | 4429 (2121-7128) | 1 | - | - | - | - | - | - | - | - | - | - |
| Fc$\gamma$R3B binding (biotinylated) | 13 | 1182 (1081-2154) | 22 | 989 (862-1406) | 0.59 | 13 | 2221 (1384-3237) | 22 | 2718 (1334-4602) | 1 | - | - | - | - | - | - | - | - | - | - |
