# Supplementary material for: Vi-specific serological correlates of protection for typhoid fever
Source: J Exp Med. 2020 Nov 12;218(2):e20201116. doi: 10.1084/jem.20201116 (PMC7668386; doi:10.1084/jem.20201116)
Supplement: Table S6 — compares Vi-specific responses between diagnosed and protected participants vaccinated with Vi-TT and between diagnosed and protected Vi-PS vaccines, using fold change. [file JEM_20201116_TableS6.docx]

**Table S6 A. Comparison of fold-change in Vi-specific measures between diagnosed and protected individuals following Vi-TT vaccination**

Fold change using baseline values were calculated for each of the postvaccination time points. Comparisons of responses between diagnosed and protected individuals were performed using Mann Whitney *U* tests. Both unadjusted and adjusted P values, using the Bonferroni correction method, are presented (non-significant P values >0.05 were rounded to two decimal places). Bolded P values are statistically significant.

|  | **Day 28** | | | | | | **Day 118** | | | | | | **Day 208** | | | | | |
| --- | --- | --- | --- | --- | --- | --- | --- | --- | --- | --- | --- | --- | --- | --- | --- | --- | --- | --- |
|  | **Diagnosed** | | **Protected** | | **P value** | | **Diagnosed** | | **Protected** | | **P value** | | **Diagnosed** | | **Protected** | | **P value** | |
|  | ***n*** | **Median (IQR)** | ***n*** | **Median (IQR)** | **Unadjusted** | **Adjusted** | ***n*** | **Median (IQR)** | ***n*** | **Median (IQR)** | **Unadjusted** | **Adjusted** | ***n*** | **Median (IQR)** | ***n*** | **Median (IQR)** | **Unadjusted** | **Adjusted** |
| **Antibody quantification** |  |  |  |  |  |  |  |  |  |  |  |  |  |  |  |  |  |  |
| IgG titer | 13 | 101.1 (67.3-151.4) | 24 | 144.1 (68.1-223.7) | 0.19 | 1 | 11 | 29.8 (17.7-61.4) | 22 | 75.9 (34.1-149.2) | **0.034** | 0.48 | 11 | 29.3 (13.8-38.9) | 23 | 67.1 (39.5-179.5) | **0.022** | 0.31 |
| IgG1 titer | - | - | - | - | - | - | - | - | - | - | - | - | - | - | - | - | - | - |
| IgG1 MFI | 13 | 90.5 (48.4-149.3) | 24 | 72.2 (43.7-135.1) | 0.99 | 1 | - | - | - | - | - | - | - | - | - | - | - | - |
| IgG1 MFI (biotinylated) | 13 | 12.4 (6.7-33.1) | 24 | 21.4 (4.4-55.0) | 0.74 | 1 | 10 | 11.9 (5.6-23.1) | 22 | 12.8 (6.1-25.8) | 0.92 | 1 | 11 | 7.3 (2.6-14.1) | 21 | 9.9 (3.2-19.4) | 0.61 | 1 |
| IgG2 titer |  |  |  |  | - | - | - | - | - | - | - | - | - | - | - | - | - | - |
| IgG2 MFI | 13 | 19.2 (13.8-26.3) | 24 | 26.6 (17.1-53.1) | 0.36 | 1 | - | - | - | - | - | - | - | - | - | - | - | - |
| IgG2 MFI (biotinylated) | 13 | 22.0 (13.5-54.9) | 24 | 24.0 (9.8-90.7) | 0.84 | 1 | - | - | - | - | - | - | - | - | - | - | - | - |
| IgG3 titer | - | - | - | - | - | - | - | - | - | - | - | - | - | - | - | - | - | - |
| IgG3 MFI (biotinylated) | 13 | 2.2 (1.0-4.0) | 24 | 2.7 (1.3-4.1) | 0.58 | 1 | 10 | 2.6 (1.3-3.7) | 22 | 2.1 (1.3-3.0) | 0.87 | 1 | 11 | 1.8 (1.0-3.2) | 21 | 2.0 (1.0-3.2) | 0.69 | 1 |
| IgA titer | 10 | 12.2 (5.3-57.0) | 23 | 57.3 (28.5-129.0) | **0.027** | 0.81 | 10 | 5.2 (3.5-29.0) | 21 | 33.7 (12.0-84.8) | **0.026** | 0.37 | 10 | 6.0 (3.8-28.0) | 21 | 37.4 (12.1-61.9) | **0.027** | 0.37 |
| IgA MFI | 12 | 97.8 (26.3-154.9) | 20 | 207.5 (87.6-584.7) | **0.041** | 1 | - | - | - | - | - | - | - | - | - | - | - | - |
| IgA MFI (biotinylated) | 9 | 3.0 (1.5-10.0) | 14 | 2.1 (1.7-3.7) | **0.019** | 0.58 | 7 | 2.2 (1.5-3.5) | 12 | 1.7 (1.2-3.8) | **0.014** | 0.19 | 8 | 2.3 (1.8-5.3 | 14 | 2.0 (1.5-3.5) | **0.003** | **0.047** |
| IgA1 MFI | 11 | 10.8 (6.2-59.7) | 24 | 56.0 (24.5-90.6) | **0.038** | 1 | - | - | - | - | - | - | - | - | - | - | - | - |
| IgA2 MFI | 12 | 9.3 (1.9-22.8) | 24 | 14.2 (5.2-83.8) | 0.16 | 1 | - | - | - | - | - | - | - | - | - | - | - | - |
| IgM titer | 10 | 56.8 (6.7-129.8) | 22 | 21.4 (13.2-47.9) | 0.98 | 1 | - | - | - | - | - | - | - | - | - | - | - | - |
| **Functional properties** |  |  |  |  |  |  |  |  |  |  |  |  |  |  |  |  |  |  |
| ADCD (biotinylated) | 5 | 22.6 (9.3-47.8) | 3 | 28.6 (15.3-36.9) | 1 | 1 | 4 | 18.8 (6.0-44.4) | 3 | 11.7 (6.9-12.2) | 0.86 | 1 | 4 | 5.12 (1.2-15.0) | 3 | 4.3 (3.2-7.6) | 0.86 | 1 |
| ADCP (biotinylated) | 13 | 2.3 (1.6-2.8) | 24 | 2.5 (1.5-6.0) | 0.76 | 1 | 10 | 2.2 (1.5-4.4) | 22 | 2.0 (1.7-3.2) | 0.98 | 1 | 11 | 1.1 (0.9-4.1) | 21 | 2.1 (0.9-2.9) | 0.78 | 1 |
| ADNP (biotinylated) | 13 | 10.3 (3.5-15.5) | 22 | 12.6 (5.2-34.1) | 0.35 | 1 | 10 | 5.7 (1.5-12.9) | 20 | 9.7 (3.5-24.6) | 0.30 | 1 | 11 | 1.2 (1.0-4.8) | 19 | 3.9 (1.3-11.0) | 0.30 | 1 |
| ADNOB (biotinylated) | 11 | -9.0 (-11.3-  -0.9) | 22 | 2.9 (-2.4-13.6) | **0.037** | 1 | - | - | - | - | - | - | - | - | - | - | - | - |
| ADNKA CD107a (biotinylated) | 11 | 0.98 (0.9-1.1) | 23 | 1.2 (1.0-1.4) | **0.027** | 0.82 | 10 | 1.0 (0.9-1.2) | 21 | 1.1 (1.0-1.3) | 0.46 | 1 | 11 | 1.1 (1.0-1.4) | 20 | 1.2 (0.9-1.4) | 0.76 | 1 |
| ADNKA MIP-1$\beta$ (biotinylated) | 11 | 2.1 (1.6-2.8) | 23 | 2.2 (1.6-3.0) | 0.97 | 1 | 10 | 2.1 (1.4-2.6) | 21 | 1.9 (1.6-2.9) | 0.66 | 1 | 11 | 1.6 (1.0-2.3) | 20 | 1.8 (1.4-2.7) | 0.44 | 1 |
| ADNKA IFN$\gamma$ (biotinylated) | 11 | 1.1 (0.9-1.3) | 23 | 1.2 (0.8-1.4) | 0.71 | 1 | 10 | 1.1 (1.0-1.5) | 21 | 1.3 (1.0-1.7) | 0.46 | 1 | 11 | 1.2 (1.1-1.5) | 20 | 1.1 (0.9-1.7) | 0.76 | 1 |
| **Fc receptor binding** |  |  |  |  |  |  |  |  |  |  |  |  |  |  |  |  |  |  |
| Fc$\alpha$R (biotinylated) | 13 | 7.8 (5.9-11.0) | 24 | 16.0 (7.2-20.3) | 0.05 | 1 | - | - | - | - | - | - | - | - | - | - | - | - |
| Fc$\gamma$R2A binding (biotinylated) | 13 | 4.2 (4.1-5.7) | 24 | 5.4 (4.5-7.5) | 0.18 | 1 | - | - | - | - | - | - | - | - | - | - | - | - |
| Fc$\gamma$R2B binding (biotinylated) | 13 | 2.7 (1.8-3.2) | 24 | 3.2 (2.1-6.5) | 0.27 | 1 | - | - | - | - | - | - | - | - | - | - | - | - |
| Fc$\gamma$R3A binding (biotinylated) | 13 | 6.9 (5.2-8.3) | 24 | 7.9 (5.1-9.8) | 0.67 | 1 | - | - | - | - | - | - | - | - | - | - | - | - |
| Fc$\gamma$R3B binding (biotinylated) | 13 | 8.1 (2.8-9.9) | 24 | 6.7 (3.7-11.4) | 0.89 | 1 | - | - | - | - | - | - | - | - | - | - | - | - |

**Table S6 B. Comparison of fold-change in Vi-specific measures between diagnosed and protected individuals following Vi-PS vaccination**

Fold change using baseline values were calculated for each of the postvaccination time points. Comparisons of responses between diagnosed and protected individuals were performed using Mann Whitney *U* tests. Both unadjusted and adjusted P values, using the Bonferroni correction method, are presented (non-significant P values >0.05 were rounded to two decimal places).

|  | **Day 28** | | | | | | **Day 118** | | | | | | **Day 208** | | | | | |
| --- | --- | --- | --- | --- | --- | --- | --- | --- | --- | --- | --- | --- | --- | --- | --- | --- | --- | --- |
|  | **Diagnosed** | | **Protected** | | **P value** | | **Diagnosed** | | **Protected** | | **P value** | | **Diagnosed** | | **Protected** | | **P value** | |
|  | ***n*** | **Median (IQR)** | ***n*** | **Median (IQR)** | **Unadjusted** | **Adjusted** | ***n*** | **Median (IQR)** | ***n*** | **Median (IQR)** | **Unadjusted** | **Adjusted** | ***n*** | **Median (IQR)** | ***n*** | **Median (IQR)** | **Unadjusted** | **Adjusted** |
| **Antibody quantification** |  |  |  |  |  |  |  |  |  |  |  |  |  |  |  |  |  |  |
| IgG Titre | 13 | 17.0 (5.7-33.3) | 22 | 32.3 (18.2-110.1) | 0.05 | 1 | 10 | 18.7 (12.0-29.1) | 19 | 44.0 (15.2-101.5) | 0.12 | 1 | 11 | 15.2 (10.9-31.6) | 18 | 44.8 (24.1-82.3) | **0.029** | 0.41 |
| IgG1 Titre | - | - | - | - | - | - | - | - | - | - | - | - | - | - | - | - | - | - |
| IgG1 MFI | 13 | 5.4 (1.0-15.0) | 22 | 15.1 (4.7-53.1) | 0.06 | 1 | - | - | - | - | - | - | - | - | - | - | - | - |
| IgG1 MFI (biotinylated) | 13 | 2.7 (1.9-6.6) | 22 | 4.1 (1.0-16.7) | 0.71 | 1 | 11 | 2.4 (1.8-4.3) | 19 | 1.8 (1.0-11.2) | 0.97 | 1 | 10 | 2.4 (1.5-4.1) | 17 | 1.5 (1.0-9.7) | 0.94 | 1 |
| IgG2 Titre | - | - | - | - | - | - | - | - | - | - | - | - | - | - | - | - | - | - |
| IgG2 MFI | 13 | 5.1 (1.0-10.3) | 22 | 9.6 (1.3-29.3) | 0.13 | 1 | - | - | - | - | - | - | - | - | - | - | - | - |
| IgG2 MFI (biotinylated) | 13 | 7.4 (2.4-14.6) | 22 | 9.7 (2.2-25.7) | 0.46 | 1 | 11 | 10.7 (5.5-16.1) | 19 | 21.6 (4.6-48.1) | 0.25 | 1 | 10 | 9.4 (2.8-15.8) | 17 | 24.2 (2.9-40.2) | 0.20 | 1 |
| IgG3 Titre | - | - | - | - | - | - | - | - | - | - | - | - | - | - | - | - | - | - |
| IgG3 MFI (biotinylated) | 13 | 1.0 (1.0-1.0) | 22 | 1.0 (1.0-1.7) | 0.60 | 1 | 11 | 1.0 (1.0-1.0) | 19 | 1.0 (1.0-1.0) | 0.69 | 1 | 10 | 1.0 (1.0-1.0) | 17 | 1.0 (1.0-1.0) | 0.18 | 1 |
| IgA Titre | 13 | 5.2 (1.2-11.4) | 21 | 23.0 (18.4-41.0) | **0.014** | 0.42 | 11 | 5.1 (1.7-11.9) | 18 | 24.33 (16.6-38.5) | **0.033** | 0.46 | 10 | 6.3 (1.3-12.3) | 18 | 22.6 (13.2-40.0) | **0.042** | 0.58 |
| IgA MFI | 13 | 15.4 (4.7-39.0) | 21 | 82.3 (39.0-147.6) | **0.007** | 0.21 | - | - | - | - | - | - | - | - | - | - | - | - |
| IgA MFI (biotinylated) | 13 | 22.2 (8.4-54.7) | 21 | 58.0 (36.4-107.3) | 0.05 | 1 | 11 | 11.1 (6.9-50.3) | 19 | 45.9 (21.9-83.7) | 0.13 | 1 | 10 | 20.2 (7.0-56.3) | 17 | 56.1 (24.3-95.8) | 0.13 | 1 |
| IgA1 MFI | 12 | 7.3 (4.0-19.0) | 22 | 18.4 (7.0-39.2) | 0.13 | 1 | - | - | - | - | - | - | - | - | - | - | - | - |
| IgA2 MFI | 12 | 2.7 (1.0-8.1) | 21 | 15.5 (2.4-26.3) | 0.10 | 1 | - | - | - | - | - | - | - | - | - | - | - | - |
| IgM Titre | 13 | 8.8 (5.4-10.5) | 21 | 12.0 (5.4-28.8) | 0.17 | 1 | - | - | - | - | - | - | - | - | - | - | - | - |
| **Functional properties** |  |  |  |  |  |  |  |  |  |  |  |  |  |  |  |  |  |  |
| ADCD (biotinylated) | 2 | 4.3 (4.0-4.7) | 2 | 12.5 (7.5-17.5) | 0.70 | 1 | 2 | 2.2 (1.8-2.6) | 2 | 10.3 (5.8-14.9) | 0.70 | 1 | 2 | 2.0 (1.7-2.3) | 1 | 12.0 (12.0-12.0) | 0.54 | 1 |
| ADCP (biotinylated) | 13 | 1.8 (1.6-2.7) | 22 | 1.6 (1.1-2.4) | 0.28 | 1 | 11 | 2.23 (1.2-5.2) | 19 | 1.4 (0.8-2.9) | 0.32 | 1 | 10 | 2.6 (1.4-4.9) | 17 | 1.5 (0.8-4.1) | 0.47 | 1 |
| ADNP (biotinylated) | 12 | 2.3 (1.7-4.5) | 21 | 6.8 (2.6-30.1) | 0.09 | 1 | 10 | 1.6 (0.8-3.6) | 18 | 4.7 (2.0-20.5) | 0.11 | 1 | 9 | 1.7 (0.9-3.3) | 16 | 7.8 (1.8-27.2) | 0.12 | 1 |
| ADNOB (biotinylated) | 12 | -1.2 (-2.5--0.1) | 19 | 0.0 (-3.7-9.7) | 0.28 | 1 | - | - | - | - | - | - | - | - | - | - | - | - |
| ADNKA CD107a (biotinylated) | 13 | 1.1 (1.0-1.4) | 20 | 1.0 (0.9-1.2) | 0.17 | 1 | 11 | 1.0 (0.9-1.2) | 17 | 1.1 (0.9-1.4) | 0.64 | 1 | 10 | 1.1 (1.0-1.3) | 15 | 1.1 (0.9-1.2) | 0.42 | 1 |
| ADNKA MIP-1$\beta$ (biotinylated) | 13 | 1.4 (1.1-1.7) | 20 | 1.9 (1.0-2.9) | 0.59 | 1 | 11 | 1.4 (1.1-1.7) | 17 | 1.5 (1.2-2.5) | 0.34 | 1 | 10 | 1.5 (1.2-1.7) | 15 | 1.5 (1.0-1.9) | 0.8 | 1 |
| ADNKA IFN$\gamma$ (biotinylated) | 13 | 1.4 (1.2-1.5) | 20 | 1.1 (1.0-1.4) | 0.14 | 1 | 11 | 1.2 (1.0-1.6) | 17 | 1.3 (1.1-1.5) | 0.85 | 1 | 10 | 1.5 (1.3-1.8) | 15 | 1.23 (1.1-1.4) | 0.19 | 1 |
| **Fc Receptor binding** |  |  |  |  |  |  |  |  |  |  |  |  |  |  |  |  |  |  |
| Fc$\alpha$R (biotinylated) | 13 | 3.7 (1.5-4.8) | 22 | 6.7 (3.4-12.6) | 0.06 | 1 | - | - | - | - | - | - | - | - | - | - | - | - |
| Fc$\gamma$R2A binding (biotinylated) | 13 | 1.8 (1.0-2.8) | 22 | 3.3 (1.7-4.8) | **0.028** | 0.80 | - | - | - | - | - | - | - | - | - | - | - | - |
| Fc$\gamma$R2B binding (biotinylated) | 13 | 1.3 (0.9-1.7) | 22 | 1.4 (1.0-1.9) | 0.46 | 1 | - | - | - | - | - | - | - | - | - | - | - | - |
| Fc$\gamma$R3A binding (biotinylated) | 13 | 1.5 (0.9-3.6) | 22 | 2.8 (1.4-5.2) | 0.10 | 1 | - | - | - | - | - | - | - | - | - | - | - | - |
| Fc$\gamma$R3B binding (biotinylated) | 13 | 1.2 (1.1-1.9) | 22 | 2.4 (1.5-4.9) | 0.08 | 1 | - | - | - | - | - | - | - | - | - | - | - | - |
