# Supplementary material for: Vi-specific serological correlates of protection for typhoid fever
Source: J Exp Med. 2020 Nov 12;218(2):e20201116. doi: 10.1084/jem.20201116 (PMC7668386; doi:10.1084/jem.20201116)
Supplement: Table S7 — presents correlations of fold change in Vi IgA titer and Vi IgG titer in diagnosed participants with clinical and laboratory indicators of typhoid fever disease severity. [file JEM_20201116_TableS7.docx]

**Table S7. Correlations of fold change in Vi IgA titer and Vi IgG titer in diagnosed participants with clinical and laboratory indicators of typhoid fever disease severity**

Spearman rho correlations were used to evaluate associations between fold change in Vi IgA titer and Vi IgG titer (baseline to day 28), and clinical or laboratory outcomes in participants diagnosed with typhoid fever (total *n* = 26; Vi-PS *n* = 13, Vi-TT *n* = 13). P values were not adjusted for multiple testing. Bolded P values are statistically significant.

|  | **Fold-change IgA titer**  (baseline to day 28) | **Fold-change IgG titer**  (baseline to day 28) |
| --- | --- | --- |
| **Time to diagnosis (h)** | *r* = 0.26 P = 0.23 | *r* = 0.29  P = 0.15 |
| **Time to first positive blood culture (h)** | *r* = 0.19 P = 0.41 | *r* = 0.37 P = 0.07 |
| **Time to first fever ≥38°C (h)** | *r* = 0.55 P = 0.05 | ***r* = 0.60 P = 0.02** |
| **Time to first positive stool culture (h)** | *r* = 0.50 P = 0.07 | *r* = 0.04 P = 0.88 |
| **Peak recorded temperature (°C)** | *r* = -0.22 P = 0.31 | ***r* = -0.43 P = 0.03** |
| **Peak CRP (mg/liter)** | *r* = -0.06 P = 0.78 | ***r* = -0.45 P = 0.02** |
| ***S*. Typhi quantification (CFU/ml)** | *r* = -0.36 P = 0.11 | ***r* = -0.44 P = 0.03** |
