# Supplementary material for: Vi-specific serological correlates of protection for typhoid fever
Source: J Exp Med. 2020 Nov 12;218(2):e20201116. doi: 10.1084/jem.20201116 (PMC7668386; doi:10.1084/jem.20201116)
Supplement: Table S8 — presents a list of 34 features measured at 28 days after vaccination, ranked by frequency of selection based on 5,000 repeated fivefold cross-validation models. [file JEM_20201116_TableS8.docx]

**Table S8.** **List of 34 features measured at 28 days after vaccination, ranked by frequency of selection based on 5,000 repeated fivefold cross-validation models**

Features in bold indicate minimal selected antibody features needed to predict nTD (red text) or TD (blue text).

| **Vi-TT and Vi-PS (Fig. 5)** | | |  | **Vi-TT (Fig. 6)** | | |  | **Vi-PS (Fig. 7)** | | |
| --- | --- | --- | --- | --- | --- | --- | --- | --- | --- | --- |
| Day 28 | | |  | Day 28 | | |  | Day 28 | | |
| Ranked Features | Frequency | CV Accuracy |  | Ranked Features | Frequency | CV Accuracy |  | Ranked Features | Frequency | CV Accuracy |
| **ViPS_IgA_MFI** | **4983** | **68.056** |  | **ADNKA_IFNg** | **4550** | **67.568** |  | **ViPS_IgA_MFI** | **4997** | **74.286** |
| **ADNKA_IFNg** | **4554** | **72.222** |  | **ADNKA_CD107a** | **4082** | **67.378** |  | **ADCP** | **4974** | **77.143** |
| **ViPS_IgA2_AI** | **2741** | **72.222** |  | **ViPS_IgA2_AI** | **2962** | **70.270** |  | **FcgR2B_Vi** | **4865** | **74.286** |
| **Vi_IgG2_Titre** | **2420** | **72.222** |  | **Vi_BIOT_IgA_AI** | **2941** | **70.270** |  | **Polyfunctionality** | **4536** | **82.857** |
| **ADNKA_MIP1b** | **1738** | **73.611** |  | **Vi_BIOT_IgG1_AI** | **2596** | **67.568** |  | **Vi_IgG2_Titre** | **4382** | **77.143** |
| Vi_BIOT_IgG2_AI | 1704 | 73.611 |  | **ADNKA_MIP1b** | **2570** | **70.270** |  | **Vi_BIOT_IgG2_MFI** | **4035** | **82.857** |
| Vi_BIOT_IgG1_MFI | 1115 | 72.222 |  | **ViPS_IgA_MFI** | **2332** | **72.973** |  | **ViPS_IgG1_AI** | **3482** | **77.143** |
| Polyfunctionality | 829 | 72.222 |  | **ViPS_IgA1_AI** | **1891** | **67.568** |  | **ViPS_IgA1_AI** | **3011** | **80.000** |
| FcgR2B_Vi | 756 | 72.222 |  | **ADCP** | **1824** | **72.973** |  | **ADNKA_IFNg** | **2896** | **74.286** |
| ViPS_IgG1_AI | 722 | 68.056 |  | **ViPS_IgG1_MFI** | **1241** | **75.676** |  | **Vi_IgM_Titre** | **2547** | **82.857** |
| ADNKA_CD107a | 670 | 69.444 |  | Vi_BIOT_IgG2_AI | 1314 | 72.973 |  | **ADNKA_CD107a** | **2174** | **82.857** |
| ViPS_IgA2_MFI | 398 | 68.056 |  | Vi_BIOT_IgG2_MFI | 844 | 75.676 |  | **Vi_BIOT_IgG2_AI** | **2090** | **82.857** |
| ADCP | 412 | 68.056 |  | Vi_IgM_Titre | 777 | 64.865 |  | **Vi_IgG3_Titre** | **1985** | **80.000** |
| Vi_BIOT_IgA_AI | 382 | 66.667 |  | ViPS_IgA2_MFI | 707 | 67.568 |  | **ViPS_IgA2_AI** | **1948** | **80.000** |
| Vi_IgM_Titre | 386 | 66.667 |  | Polyfunctionality | 562 | 64.865 |  | **Vi_IgA_Titre** | **1398** | **77.143** |
| Vi_IgA_Titre | 352 | 68.056 |  | FcgR3A_Vi | 481 | 64.865 |  | **Vi_BIOT_IgG3_MFI** | **1113** | **77.143** |
| ADNOB | 316 | 72.222 |  | ADNOB | 485 | 64.865 |  | **ViPS_IgG2_MFI** | **1122** | **80.000** |
| ADCD | 285 | 69.444 |  | Vi_IgG3_Titre | 341 | 70.270 |  | **ViPS_IgA1_MFI** | **1073** | **85.714** |
| FcgR3A_Vi | 201 | 68.056 |  | ViPS_IgG1_AI | 340 | 67.568 |  | Vi_BIOT_IgG1_MFI | 1009 | 77.143 |
| Vi_BIOT_IgG3_MFI | 113 | 66.667 |  | Vi_BIOT_IgG3_MFI | 325 | 64.865 |  | ADNKA_MIP1b | 1007 | 80.000 |
| ViPS_IgA1_AI | 118 | 66.667 |  | Vi_BIOT_IgA_MFI | 318 | 64.865 |  | ADNP | 853 | 82.857 |
| Vi_IgG3_Titre | 73 | 69.444 |  | Vi_IgG_Titre | 271 | 64.865 |  | Vi_BIOT_IgA_AI | 765 | 74.286 |
| Vi_IgG1_Titre | 72 | 69.444 |  | ViPS_IgG2_MFI | 272 | 72.973 |  | ADNOB | 769 | 77.143 |
| Vi_BIOT_IgG2_MFI | 51 | 68.056 |  | ADCD | 274 | 70.270 |  | ViPS_IgA2_MFI | 620 | 74.286 |
| Vi_BIOT_IgG1_AI | 45 | 69.444 |  | Vi_IgG1_Titre | 243 | 64.865 |  | ADCD | 619 | 60.000 |
| ViPS_IgG1_MFI | 39 | 68.056 |  | ViPS_IgA1_MFI | 240 | 67.568 |  | Vi_BIOT_IgG1_AI | 596 | 60.000 |
| ADNP | 34 | 69.444 |  | Vi_BIOT_IgG1_MFI | 157 | 70.270 |  | FcgR3A_Vi | 559 | 62.857 |
| ViPS_IgG2_MFI | 16 | 68.056 |  | FcgR2B_Vi | 181 | 64.865 |  | FcgR3B_Vi | 476 | 65.714 |
| FcAR_Vi | 8 | 65.278 |  | ADNP | 191 | 64.865 |  | Vi_IgG_Titre | 254 | 65.714 |
| FcgR3B_Vi | 8 | 68.056 |  | Vi_IgG2_Titre | 151 | 67.568 |  | Vi_BIOT_IgA_MFI | 252 | 62.857 |
| FcgR2A_Vi | 6 | 66.667 |  | FcgR3B_Vi | 140 | 67.568 |  | FcgR2A_Vi | 224 | 62.857 |
| Vi_BIOT_IgA_MFI | 5 | 65.278 |  | Vi_IgA_Titre | 109 | 67.568 |  | ViPS_IgG1_MFI | 155 | 65.714 |
| ViPS_IgA1_MFI | 4 | 68.056 |  | FcgR2A_Vi | 92 | 64.865 |  | Vi_IgG1_Titre | 141 | 62.857 |
| Vi_IgG_Titre | 2 | 68.056 |  | FcAR_Vi | 57 | 64.865 |  | FcAR_Vi | 134 | 65.714 |
